# Quantifying Directedness in Chemical Reaction Networks Using Assembly Theory

**DOI:** 10.1021/acscentsci.6c00618

**Published:** 2026-09-30

**Authors:** Michael Jirasek, Abhishek Sharma, Mary Wong, Jennifer Munro, Leroy Cronin

**Affiliations:** School of Chemistry, 3526 The University of Glasgow, University Avenue, Glasgow G12 8QQ, U.K.

## Abstract

There is currently no general experimental framework for determining whether an open-ended reaction network explores chemical space undirectedly or with some level of directedness. Assembly Theory (AT) provides a quantitative framework for mapping experimentally observed molecular ensembles into assembly space. Here we show that directedness in chemical systems can be quantified using two experimentally accessible observables, the exploration ratio (ER) and ensemble assembly (A). ER measures the fraction of the inferred joint assembly space sampled, while A integrates the molecular assembly index with the observed copy number. Together, these quantities distinguish undirected from directed exploration and, when applied to time-resolved data or matched controls, provide a quantitative measure of transitions associated with selection. By analyzing peptide ensembles generated under diverse polymerization conditions, we find that nonspecific activation conditions yield exploration ratios of 0.77–0.96, whereas sequence-selective proteases yield lower ratios of 0.51–0.75 and elevated A. These differences are consistent across multiple environments and amino-acid combinations. The results establish ER and A as experimentally tractable metrics for directedness in chemical reaction networks and provide a route to quantify selection when the relevant temporal or causal context is defined, with implications for reaction-network analysis, combinatorial synthesis, and chemical evolution.

## Graphical abstract

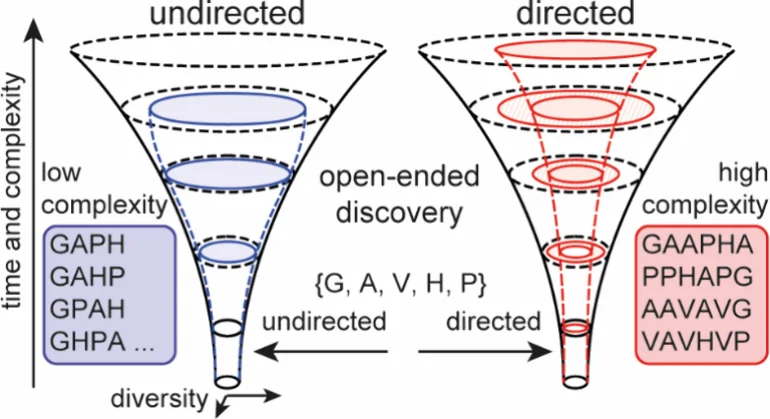

## Introduction

A central question in chemical evolution is how directedness first emerges in chemical systems before replication,^1^ heredity, and Darwinian evolution exist,^2,3^ and when such directedness constitutes selection. Although many models describe how biological evolution proceeds once genomes arise,^4–6^ there is still no universal, experimentally grounded way to determine whether an open-ended chemical system explores chemical space broadly or with directedness. Without such a measure, transitions from chemistry to biology remain difficult to compare experimentally. Assembly Theory (AT)^7^ provides a route to quantify this behavior directly from the distributions of objects produced by a chemical system by defining objects through the minimum number of steps required to construct them, their assembly index, and integrating this with their observed copy numbers through the assembly eq 1,

1 $$A=\overset{N}{\underset{n_{i}}{\sum }}e^{a_{i}}\left(\frac{n_{i}-1}{N_{T}}\right)$$

where *a*_*i*_ is the assembly index of species *i*, defined as the minimum number of join operations required to construct it from monomers, *n*_*i*_ is the copy number of species *i*, *N*_T_ is the total number of objects, and the sum runs over all *N* unique species observed in the experiment. The term *n*_*i*_ – 1 excludes singleton observations from contributing to *A*, consistent with the published formulation of ensemble assembly.^7^

AT predicts that selection should leave a measurable signature in product ensembles through the emergence and persistence of directed exploration. Here directed processes should generate ensembles enriched in high-assembly objects and restricted subsets of their combinatorial space, whereas undirected processes should explore that space broadly^8,9^ (Figure 1). A second quantity, the exploration ratio, captures how completely a system samples its Joint Assembly Space (JAS). The metric reports directedness when interpreted against matched controls or time-resolved data; a single low value does not by itself identify the physical cause or prove selection.

**Figure 1:**
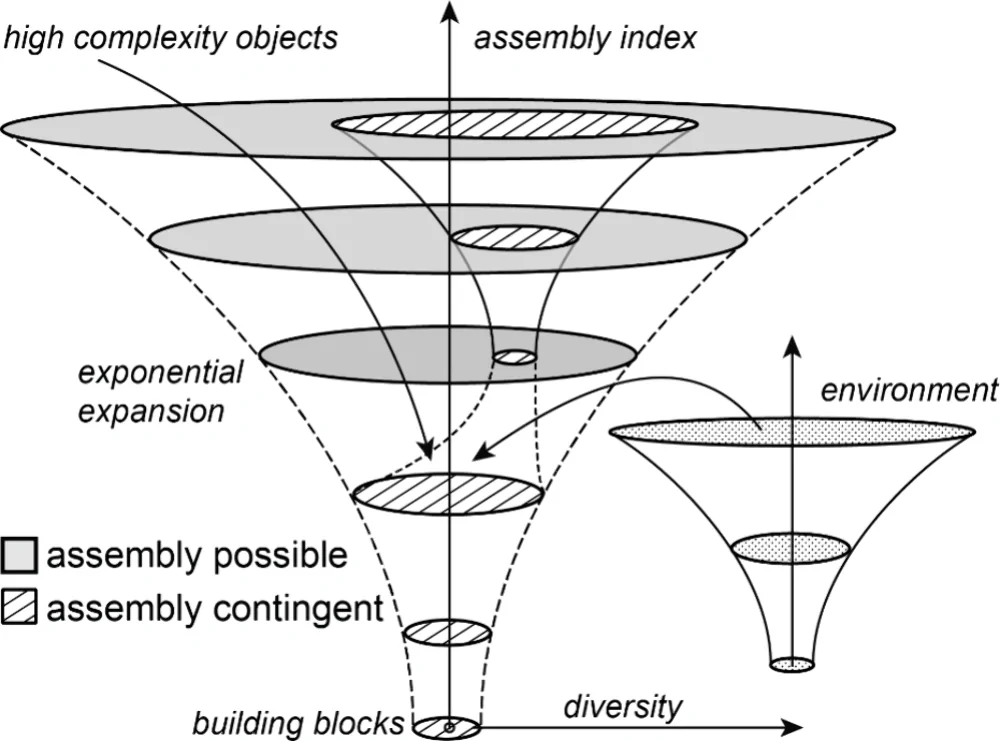
Pictographic representation of an expanding assembly space. The outer boundary represents assembly possible, while undirected exploration samples this space broadly. A physical, chemical, or environmental constraint can bias production toward a restricted assembly contingent region, producing directed exploration. When such a constraint emerges, persists, or is propagated by the system, the change can form part of a selection process. The vertical axis represents increasing assembly depth of the objects and does not require the chronological reaction pathway to increase complexity monotonically.

Throughout this work, constraint denotes a physical, chemical, or environmental influence that changes accessible pathways, rates, or product distributions. Directedness denotes an observable bias toward a restricted subset of assembly space relative to an undirected baseline. Selective catalysis is one mechanism that can impose directedness by accelerating some product-forming pathways more than others. Selection denotes a temporal transition from a less directed to a more directed regime in which particular objects or constraints persist or influence subsequent exploration. The metrics introduced here measure directedness in an observed ensemble; identifying selection additionally requires time-resolved evidence, a matched baseline, or evidence that the constraint is generated or propagated by the system.

A central question is how novel, functional catalysts could arise spontaneously through environmental interactions. Although theoretical studies have examined the time scales and dynamics by which functional molecules might emerge from chemical “seeds”. Such processes remain difficult to recognize experimentally without quantitative measures of directedness and temporal change.^10–16^ More generally, an end point bias alone cannot distinguish a fixed energetic preference from selection. Here we test whether ER and A detect controlled changes in directedness. Using peptide formation as a model open-ended chemical system,^8^ we show that nonspecific activation produces exploration ratios approaching unity, whereas evolved proteases used as externally supplied sequence-selective catalysts produce lower ratios and elevated A. The proteases provide calibrated constraints because their substrate preferences are independently characterized. The end point experiments are not taken alone as proof that the peptide network undergoes endogenous selection; they validate the metrics as measures of directed exploration under matched analytical conditions.

To validate metrics intended to quantify directedness associated with selection, they should be tested against an independently characterized selective constraint. Enzymes provide such a reference because they are products of biological evolution with well-characterized sequence specificity. Introducing them into an otherwise abiotic peptide-forming system therefore imposes a known selection-derived constraint. A valid measure of directedness should detect the resulting redistribution of the ensemble relative to nonspecific activation controls. This tests the response of the metrics to a known selective constraint without assuming that the peptide network itself undergoes endogenous selection.

## AT Computational Model Applied to Peptide Sequences

In this section, we apply the principles of AT to a forward process similar to open-ended exploration of peptide sequence space. As previously mentioned, in AT, the minimal number of steps required to construct an object from defined building blocks, the assembly index, defines its intrinsic construction complexity. For ensembles of objects, the JAS represents the minimal set of recursive steps required to construct all observed members. For small molecules this can be computed directly; for larger ensembles, such as peptide mixtures, we approximate the JAS, that is the joint assembly paths of peptides, as the graph union of individual minimal construction paths. For amino acids, assembly indices are derived from their minimal molecular construction pathways. Peptide sequences are then treated modularly by considering amino acids as building blocks connected through amide bonds. This allows the assembly index of a peptide to be approximated as *a*_P_ = *a*_JAS_ + *a*_S_, where *a*_JAS_ is the overall assembly index of the JAS of the amino acids present in the peptide chain and *a*_S_ is the assembly index of the peptide sequence represented by a string (Figure 2).

**Figure 2:**
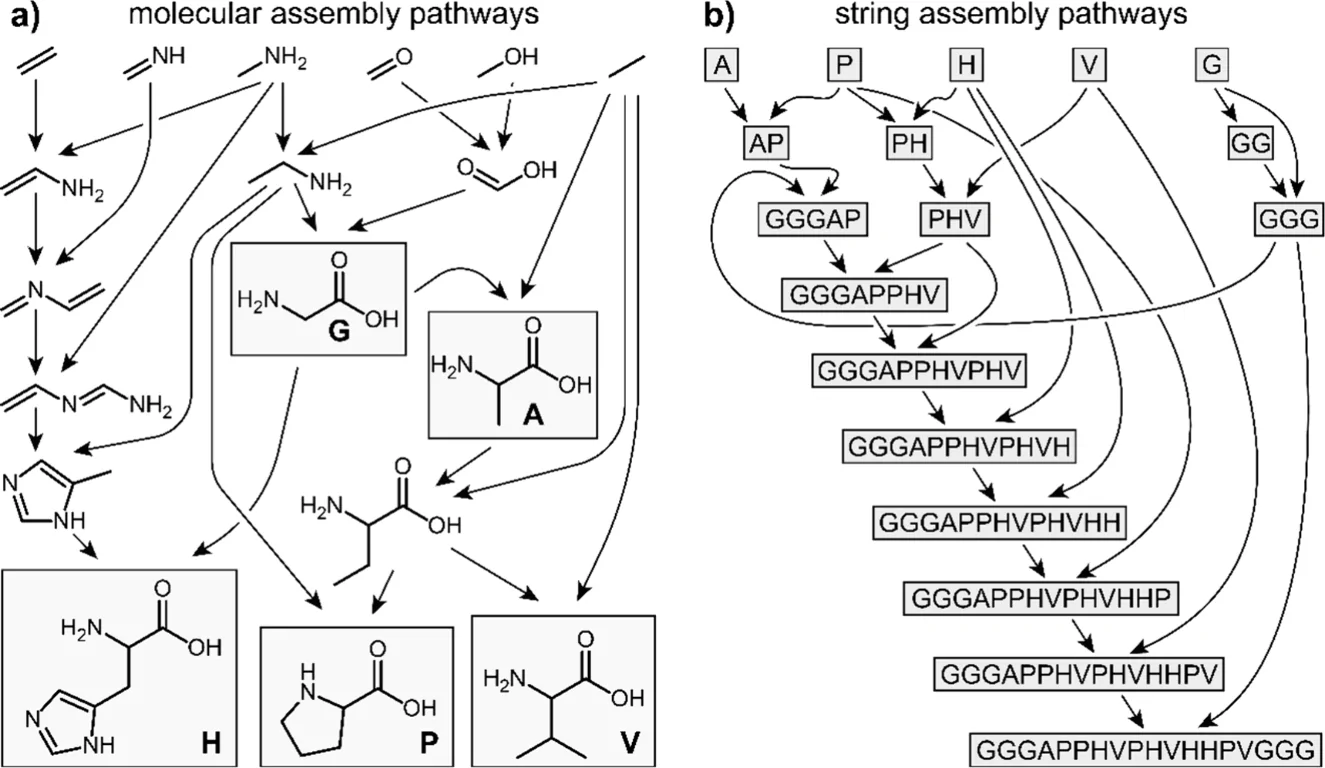
(a) JAS of five amino acids, glycine, alanine, proline, valine, and histidine, at molecular scale using chemical bonds as building blocks. (b) Assembly pathway of the peptide sequence GGGAPPHVPHVHHPVGGG using amino-acid characters as building blocks. The shortest construction pathway defines the assembly index. A balanced binary tree can organize joins into approximately logarithmic parallel depth, but the assembly index counts the total number of distinct joining operations and allows previously constructed subobjects to be reused. For a homogeneous string, repeated self-doubling can approach ceil­[log2­(*n*)]. For GGGAPPHVPHVHHPVGGG, the repeated GGG and PHV substructures can be constructed once and reused; the assembly index algorithm gives a minimum pathway of 13 joins, as depicted.

AT partitions combinatorial space into the assembly universe, containing all abstract possibilities; assembly possible, containing chemically realizable sequences; assembly contingent, containing objects reached under a particular history and set of constraints; and assembly observed, containing the experimentally detected objects. For a given observed ensemble, the inferred JAS is the union of the shortest construction pathways of all detected objects. Every node is then classified by the experiment as observed or contingent: observed objects are detected in the ensemble, whereas contingent objects are not detected but are required on at least one shortest pathway. These are disjoint subsets of the JAS. The number of contingent nodes depends on substructure reuse and on how completely the ensemble samples the nodes implied by its shortest pathways. The exploration ratio is the number of observed objects divided by the total number of observed plus contingent objects in the inferred JAS.

2 $$\mathrm{ER}=\frac{N_{\mathrm{obs}}}{N_{\mathrm{obs}}+N_{\mathrm{cont}}}$$

The exploration ratio is a comparative measure of sampling density. It should be interpreted between matched systems with the same building blocks, detection rules, and assembly conventions. Broad exploration gives a higher ratio because many pathway nodes are detected; directed exploration gives a lower ratio because production is concentrated on a restricted set of objects and more implied nodes remain contingent. A low ratio alone does not identify the cause of sparse exploration or establish selection. Ensemble assembly A integrates assembly index and copy number. Together, ER and A provide an experimental route for comparing undirected and directed peptide-forming systems.

To explore these ideas computationally, we consider five different monomers {a, b, c, d, e}, and combine them stochastically so that once a sequence is formed, it can be used recursively. Figure 3a shows the undirected exploration of sequence space, where the construction pathway (not the assembly pathway) of a long sequence is highlighted. This emerging combinatorial space of sequences therefore represents an undirected exploration without any restriction on copy number. This means that once an object is created, it can be used recursively to create new objects indefinitely, leading to extremely fast exploration of the combinatorial space. The complete combinatorial space grows faster than exponentially with respect to assembly index. As an example, to demonstrate the scale of expansion, Figure 3b shows fully enumerated combinatorial space for two, three, and four monomers (defined as *n*) up to length 16 (for *n* = 2 and 3) and 15 (for *n* = 4) without considering any symmetry constraints. For assembly index *a*, the complete enumeration requires considering every combination up to length 2^*a*^, which means that, with respect to assembly index for *n* = 2 and 3, the combinatorial space is completely enumerated up to *a* = 4.

**Figure 3:**
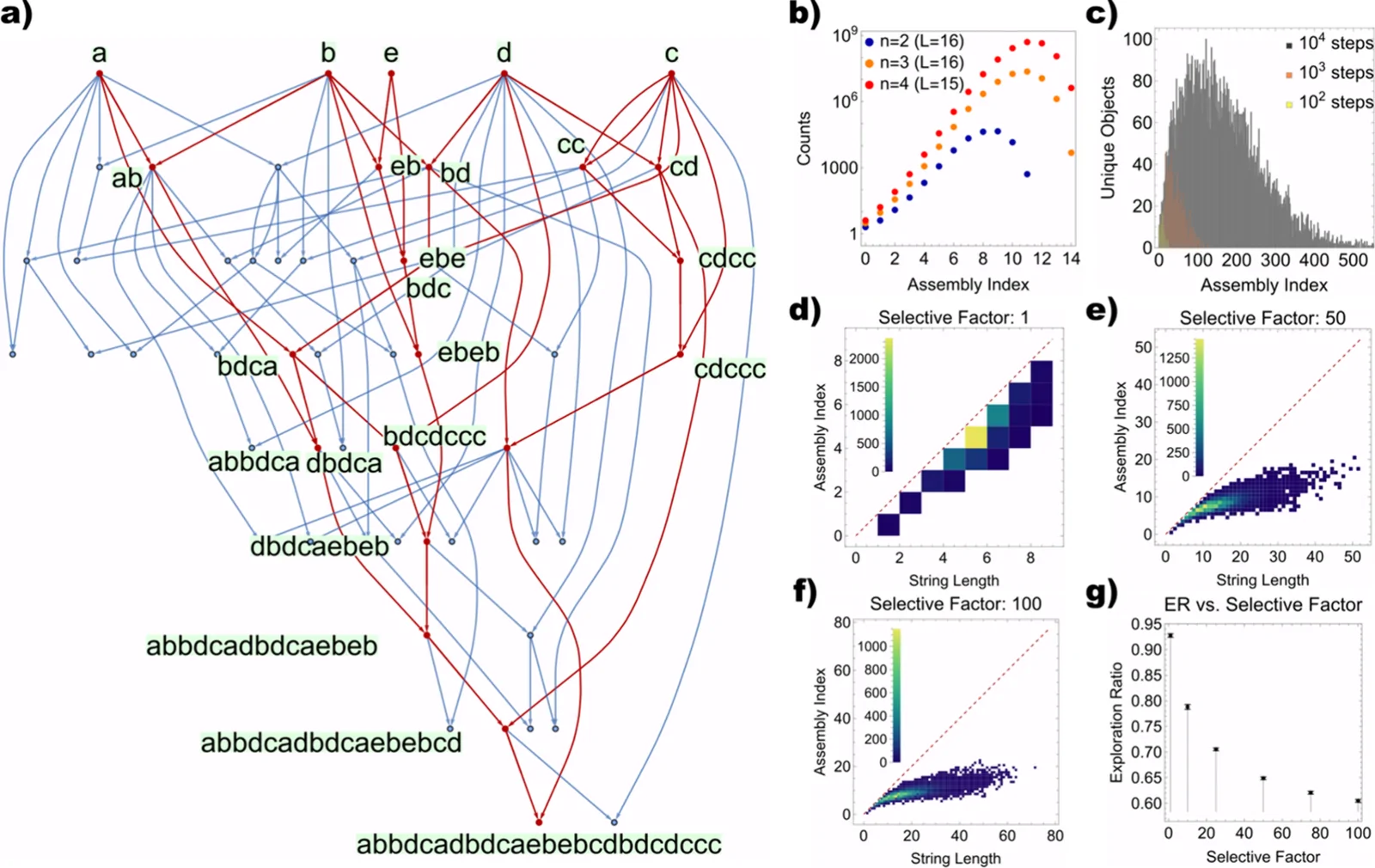
Computational experiments on expanding combinatorial sequence space. (a) Undirected recursive combination of sequences over 50 steps; one construction pathway is highlighted in red. (b) Enumeration of sequence combinations for different monomer counts up to the stated maximum lengths. (c) Assembly index distribution at different timesteps of unique objects only without a copy-number limit; assembly index calculations used a 600 s timeout. (d–f) Representative single-run density histograms of assembly index versus string length at selectivity factors *w* = 1, 50, and 100. (g) Exploration ratio across six values of *w*, shown as the mean and variation over 16 independent simulations at each value.

For higher assembly indices, due to incomplete enumeration, the number falls. In the forward dynamics, without any restriction on copy number, a finite set of stochastic events along the recursive pathways could substantially change the structure of the emerging assembly space, and objects with high assembly indices emerge (Figure 3c). However, combinatorial expansion in the chemical systems is often limited by mass transfer kinetics, and copy number plays a key role. Here, we represent the unexplored assembly space by a stochastic reaction network in which objects are generated recursively through combinations with or without an imposed bias. Previously, rule-based chemical space exploration has been used to define chemistry as a recursive expanding reaction network generated by iterative application of fixed reaction rules.^17,18^

As a phenomenological model inspired by the experimental design, we begin with five monomers at a fixed initial copy number. At each combination step, sequences are selected in proportion to their current copy numbers, limiting the otherwise unrestricted combinatorial expansion. Directedness is imposed through a selectivity factor *w* that favors sequences beginning or ending in b. The unbiased condition is *w* = 1, while larger values increase the preference for b-terminated sequences. We reran the model at six values of *w*, spanning *w* = 1–100, with 16 independent repeats at each value. Parts d–f of Figure 3 show representative single-run assembly index versus string-length distributions for *w* = 1, 50, and 100; Figure 3g reports the exploration ratio across all six values, averaged over the 16 repeats. In the unbiased regime, the population contains many heterogeneous strings with little reusable substructure, so their assembly indices lie near the linear upper bound. As *w* increases, the model produces longer, b-rich and increasingly periodic strings. Repeated motifs can be constructed once and reused, reducing the number of distinct joins and shifting the scaling toward the lower, approximately logarithmic-like regime. Longer strings increase ensemble complexity, while periodicity partly offsets that increase. Across the parameter sweep, the exploration ratio decreases systematically with increasing *w*. Full model definitions, parameters, replicate statistics, and distributions are provided in Supporting Information Section 1.

## Experimental AT Applied to Reactions of Amino Acids

To quantify directedness experimentally, we explored the combinatorial peptide sequence space generated from amino-acid monomers under different constraints and activation modes. The system provides operationally undirected reference conditions and conditions in which sequence-selective proteases impose directedness. Enzymatic catalysis was chosen because the substrate preferences of the proteases are independently characterized, providing a controlled selective constraint rather than an arbitrary source of bias.

The experiments therefore test whether ER and A detect a known redistribution of the product ensemble; they do not assume that every enzyme-mediated end point distribution is itself proof of an endogenous selection process. The degree of directedness can then be compared using the exploration ratio and ensemble assembly A. By default, we use five amino acids, glycine (G), l-alanine (A), l-valine (V), l-histidine (H), and l-proline (P), as unprotected amino acids for heat-driven and carbonyldiimidazole (CDI)-activated polymerization, and the corresponding methyl esters for enzyme-facilitated polymerization. The amino acids were selected to limit oligomer mass, retain aqueous solubility, and provide distinct exact masses suitable for mass-spectrometric analysis. Broader screens used l-phenylalanine (F), l-glutamic acid (E), l-aspartic acid (D), l-cysteine (C), l-serine (S), l-ornithine (O), and L-tryptophan (W).

To realize undirected exploration under different environmental constraints, we polymerized amino acids using three activation modes: heat-driven wet–dry cycling, CDI-mediated coupling in aqueous solution, and CDI-mediated coupling in the solid state. Wet–dry cycling was performed robotically (Opentrons) using XDL^19^ procedures, with 50 mM amino acid mixtures heated at 130 °C for 4 h, resuspended, sampled (15%), replenished with fresh monomers, and reannealed. Solution-phase CDI activation involved mixing amino acids (0.2 M) with CDI (0.5 M) at 40 °C for 1 h, followed by sampling and replenishment. Solid-state CDI coupling was performed in a ball mill (ZrO_2_ media) at 25 Hz for 90 min, with CDI readded between cycles. All undirected conditions produced steadily expanding peptide ensembles. To impose directed exploration, we used sequence-selective proteases: papain, bromelain, trypsin, and chymotrypsin, to catalyze peptide formation from amino acid methyl esters in the solid state. Following literature protocols,^20,21^ amino acid esters were milled with enzyme (10 wt %), l-cysteine for cysteine proteases (5 wt %), and sodium carbonate (1 equiv), with samples taken after each milling cycle.

## Reaction Mixture Analysis

Samples were resuspended in water, filtered, and analyzed by HPLC–MS/MS. Data-dependent acquisition selected the 20 most intense MS1 features for HCD fragmentation at multiple collision energies. MS/MS data were processed using the OLIGOSS software,^22^ which assigns peptide sequences by matching parent masses to all plausible formulas, validating them through exhaustive b/y fragment searches.^23^ Sequences were accepted only when ≥70% of expected fragments were detected, ensuring confident annotation of peptides present at sufficient abundance (Figure 4).

**Figure 4:**
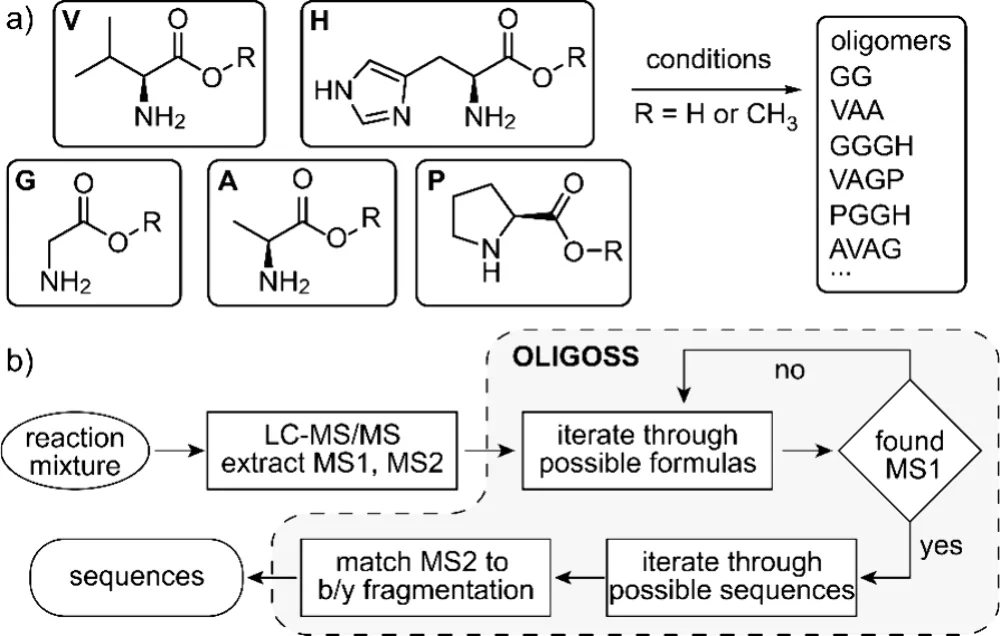
Experimental pipeline for generating and quantifying peptides in an open-ended process. (a) Combinatorial expansion is initiated by heat, a coupling agent, or sequence-selective enzymatic catalysis. (b) Samples are analyzed by HPLC-MS/MS, and OLIGOSS^22^ uses the parent and fragment-ion data to annotate observed peptide sequences.

All sequences detected above the 70% fragment-coverage threshold were assigned unit copy number, making the experimental analysis a presence-or-absence measurement. This choice avoids treating raw MS1 intensities as relative abundance proxies because electrospray ionization (ESI) efficiency depends strongly on peptide composition and length. A bounded sensitivity analysis recalculated ensemble assembly under opposing monotonic abundance gradients from 0.5-fold to 5-fold across the assembly index range. All four reaction families remain separated under both scenarios, showing that the classification is not an artifact of equal weighting (Figure S55).

To compute the ensemble assembly A, we estimated the polymer assembly of each sequence defined as a string with amino acids as building blocks and assigned copy numbers. Because ESI efficiencies differ widely among peptides and intensities are not quantitatively comparable, we treated all detected species as having equal effective copy number, consistent with their being above the detection threshold.^24,25^ For each sequence, the shortest assembly pathway was generated,^26^ and the assembly path was stored for further analysis. For each ensemble, the JAS path was approximated as the union of the individual paths. Using the generated JAS, the exploration ratio, approximated ensemble complexity, and diversity (measured as the number of unique objects observed) were calculated using Python code, for further information see Code Availability section below.

## Analysis and Discussion

To evaluate exploration behavior across recursive cycles, we carried out polymerization experiments under varied conditions, building-block sets, and activation modes. Exploration ratio and ensemble assembly A were calculated for each run using eqs 1 and 2; key experiments were replicated and blanks were analyzed to exclude artifacts. Across the nonspecific activation conditions, solution-phase CDI, wet–dry cycling, and solid-state CDI, exploration ratios remained high, typically ranging from 0.77 to 0.96, while peptide diversity increased. Protease-mediated polymerization produced a distinct pattern. Papain and bromelain began with lower ratios that increased with the cycle number, while trypsin and chymotrypsin maintained intermediate ratios near 0.7. Ensemble assembly further separated the regimes: protease reactions showed larger A per unique object than the nonspecific activation conditions. Overall, the matched experimental systems distinguish broadly exploring reference regimes from protease-imposed directed regimes. The lower ER and higher A are not generic signatures of catalysis, because catalysis can alter rate without changing the relative product distribution. Here they report a reproducible redistribution associated with the independently characterized sequence selectivity of the proteases (Figure 5).

**Figure 5:**
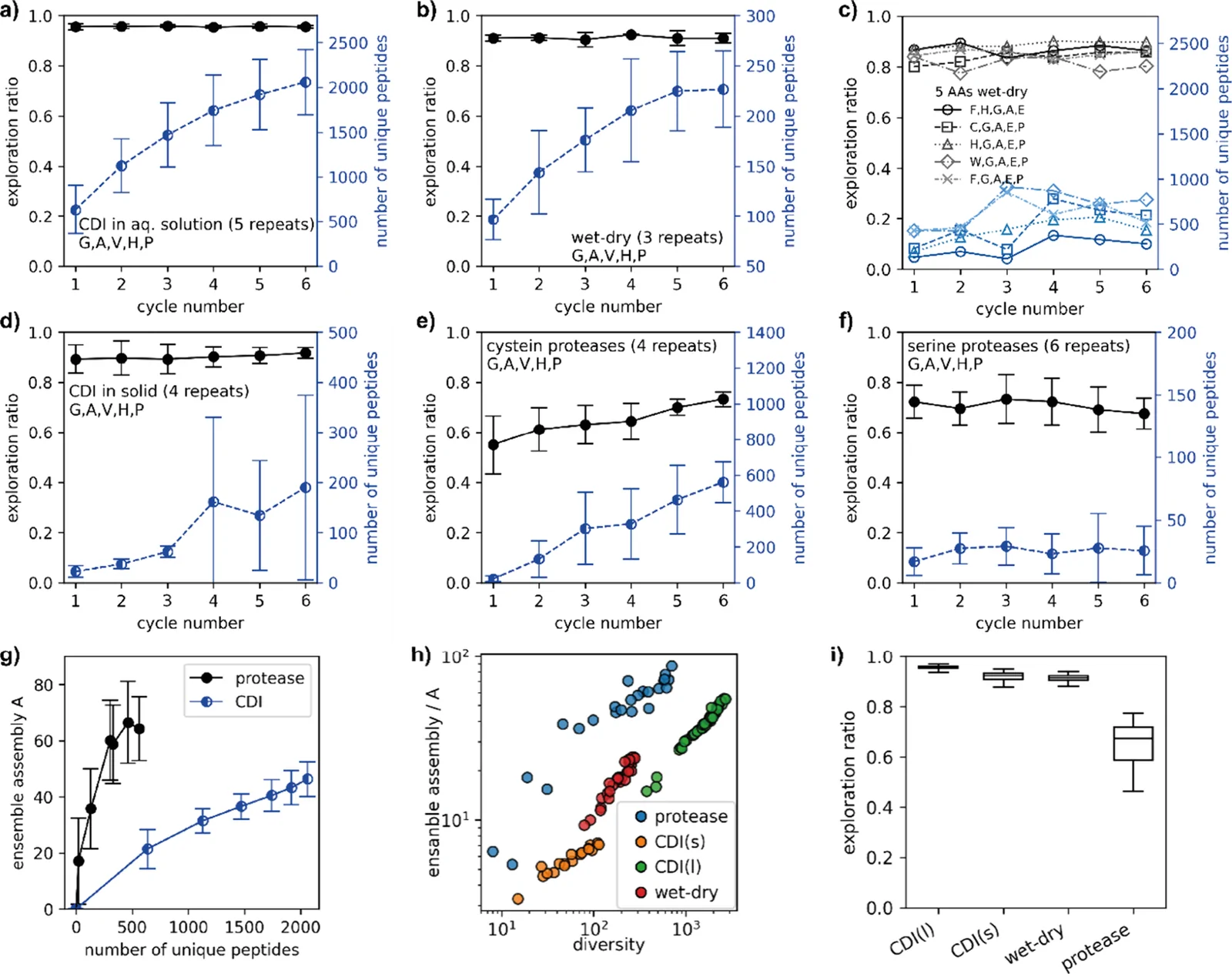
Comparison of metrics for operationally undirected and protease-directed peptide-forming systems. Exploration-ratio trajectories over six polymerization cycles, with error bars showing the standard deviation across independent replicate experiments: (a) solution-phase CDI; (b) wet–dry cycling; (c) wet–dry cycling with different building-block sets; (d) solid-state CDI; (e) cysteine proteases; (f) serine proteases. The protease conditions show lower exploration ratios than the nonspecific activation references. (g) Ensemble assembly A as a function of the number of unique products for cysteine-protease and solution-phase CDI conditions. (h) Characteristic ensemble assembly plotted against diversity for a subset of ensembles with G, A, V, H and P building blocks: cysteine-protease, solution-phase CDI, solid-state CDI and wet–dry conditions (based on selected experiments from (a–f)). (i) Exploration-ratio distributions for the same ensembles as in (h). ER is interpreted comparatively and is not an absolute indicator of selection from a single ensemble.

According to peptide database MEROPS,^27^ papain exhibits strong and distinctive substrate preferences across its active-site subsites, conventionally numbered P1, P2, ... and P1′, P2′, ... respectively for amino acid residue positions N-terminal and C-terminal to the cleaved/formed peptide bond. At P2, it favors valine, alanine, and proline, indicating good accommodation of branched aliphatic residues. At P1, glycine is strongly preferred and proline is poorly tolerated, reflecting the need for small, flexible residues at the scissile bond. The P1′ position similarly favors alanine and glycine, with proline completely blocking reactivity. P2′ is more permissive, tolerating glycine and proline well and showing moderate preference for histidine. Such selectivity is intrinsically sequence-specific and not reducible to a simple terminal amino acid reactivity. These patterns explain the selective sequence enrichment observed in papain-mediated polymerization.

To understand the structural basis of the different exploration ratios, we compare JAS representations from the G, A, V, H, and P amino-acid space using ensembles with similar numbers of unique sequences. One data set is an undirected reference generated using CDI, and the other is a directed reference generated using papain (Figure 6). In the CDI ensemble, the distribution of polymer assembly (PA) indices is concentrated at lower values and decreases rapidly at higher PA. In the papain ensemble, the distribution is shifted toward higher PA and the inferred JAS contains more contingent nodes. Papain recognizes extended sequence context across several positions around the reacting bond, so its effects cannot be reduced to the reactivity of a single terminal amino acid.

**Figure 6:**
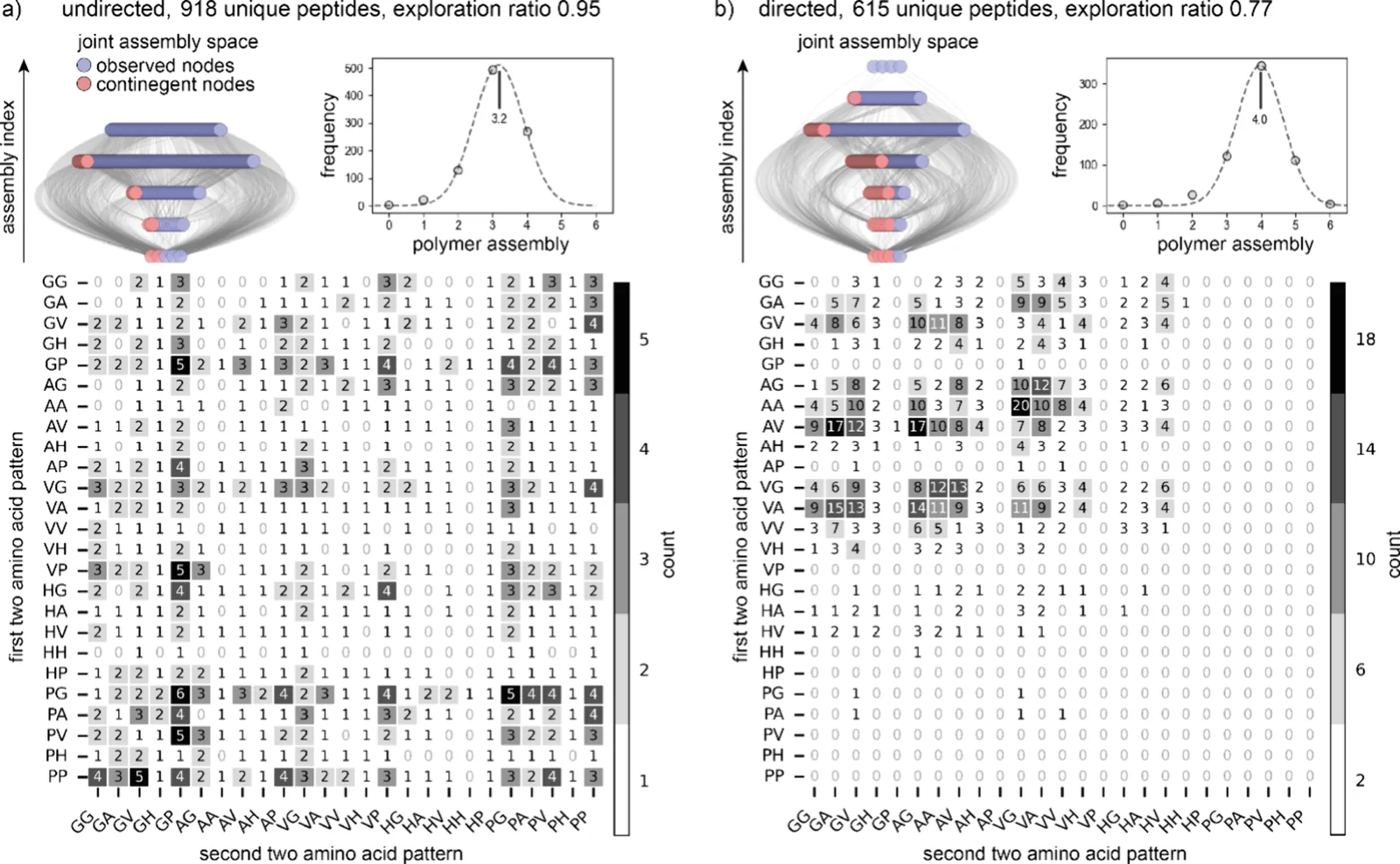
Comparison of two ensembles generated in the G, A, V, H, and P sequence space. (a) Operationally undirected reference generated by CDI-mediated coupling. (b) Directed reference generated by papain in the solid state. The papain-directed JAS contains more contingent, unobserved nodes, shown in red, and its observed products are shifted toward higher polymer assembly. The bigram matrices show occurrences of four-residue patterns. The CDI ensemble is more evenly distributed, whereas the papain ensemble contains concentrated patterns consistent with the enzyme’s extended substrate selectivity.

A more detailed overview of various experiments is summarized in Table 1.

**Table 1:** Survey of Representative Experiments and Summary Values for Diversity and Exploration Ratio (ER)^a^

type	composition/amino acids	diversity	ER(first)	ER(last)	cycles
CDI (aq.)	G, A, V, H, P	383–1846	0.96 ± 0.00	0.95 ± 0.00	8
CDI (aq.)	G, A, V, H, P [31:17:18:33:1]	137–1809	0.78 ± 0.01	0.80 ± 0.02	11
CDI (aq.)	G, A, V, H, P [32:16:15:31:6]	187–1591	0.79 ± 0.04	0.77 ± 0.01	11
CDI (aq.)	G, A, V, H, P [23:39:4:21:13]	191–1639	0.78 ± 0.02	0.78 ± 0.03	11
CDI (s.)	G, A, V, H, P	31–114	0.93 ± 0.02	0.93 ± 0.01	6
wet–dry	H, G, A, E, P	192–574	0.88 ± 0.01	0.90 ± 0.00	6
wet–dry	F, G, A, E, P	427–859	0.86 ± 0.01	0.85 ± 0.01	6
wet–dry	F, H, G, A, E	116–374	0.88 ± 0.01	0.87 ± 0.01	6
wet–dry	C, G, A, E, P	218–785	0.81 ± 0.01	0.86 ± 0.00	6
wet–dry	W, G, A, E, P	421–916	0.81 ± 0.03	0.79 ± 0.01	6
wet–dry	V, O, D	81–149	0.93 ± 0.01	0.92 ± 0.02	10
wet–dry	G, D, H, S	501–896	0.96 ± 0.00	0.94 ± 0.01	7
protease	trypsin, G, A, V, H, P	36–55	0.71 ± 0.06	0.69 ± 0.05	6
protease	chymotrypsin, G, A, V, H, P	19–76	0.74 ± 0.06	0.75 ± 0.03	6
protease	papain, G, V, A, H, P	8–701	0.51 ± 0.01	0.73 ± 0.01	6
protease	bromelain, G, V, A, H, P	13–587	0.64 ± 0.08	0.69 ± 0.02	6

a Each row represents a single experimental trajectory rather than an average across replicate experiments. CDI denotes CDI-mediated coupling; aq. indicates aqueous solution, and s. indicates solid state. Diversity is the range between the minimum and maximum numbers of unique sequences observed over the trajectory. ER­(first) and ER­(last) are the mean ± standard deviation of the first two and last two cycles, respectively, rounded to two decimal places. The ranges illustrate changes or stability in ER during exploration. Further analysis is provided in Supporting Information Section 3.

To test whether the separation depended on the default amino-acid set, we examined additional compositions. We also visualized the JAS of matched ensembles, where red nodes represent inferred but unobserved contingent peptides and blue nodes represent experimentally observed peptides. The protease-directed ensemble contains more contingent nodes and a product distribution shifted toward higher polymer assembly. We then performed bigram transition analysis, which records how often one amino-acid pair is followed by another within four-residue segments.

Each matrix shows how often one two-amino-acid pattern is followed by another within longer peptide sequences, mapping the four-residue compositional landscape generated under each condition. Papain-generated sequences show a selective pattern that reflects the enzyme’s known substrate preferences, including enrichment of alanine- and valine-containing combinations, moderate glycine representation, and depletion of proline-containing sequences. The pattern demonstrates coordinated selectivity across several residues rather than a simple preference for one reactive group.

In contrast, CDI-mediated coupling gives a more even distribution across amino-acid combinations and a higher representation of proline-containing sequences. The reciprocal pattern, high alanine and valine with low proline under papain, and a more balanced composition under CDI, supports the interpretation that papain acts as a molecular filter. It enriches combinations compatible with its extended substrate-recognition profile and depletes disfavored sequences.

The comparison of undirected and directed exploration is relevant beyond prebiotic chemistry. ER provides a quantitative way to compare how open reaction networks sample chemical space. Any chemical condition can bias reaction rates, but a fixed bias is not by itself selection. The end point experiments reported here establish directedness relative to matched nonspecific activation controls. Selection additionally requires a temporal transition or evidence that a constraint is generated, amplified, or propagated by the system. The computational parameter sweep provides an explicit time-dependent model of increasing imposed bias, while the experimental proteases provide independently characterized selective constraints. More generally, the framework could be used to analyze complex mixtures and test how environmental constraints shape chemical evolution. Application to biosignature detection will require matched controls, temporal information, or mechanistic evidence so that sparse exploration is not confused with a fixed thermodynamic sink or analytical undersampling.

The peptide system was chosen as a controlled proof of concept because it combines unambiguous sequence assignment by MS/MS, a defined combinatorial space, and independent substrate-preference data for the proteases.^27^ The metrics are not intrinsically restricted to linear polymers. They are defined over the assembly space of observed products rather than the chronological laboratory pathway, so protection, deprotection, cyclization, fragmentation, branching, and complexity-decreasing transformations can in principle be represented under stated building blocks and joining rules. Interpretation in nonlinear networks requires care: a low ER can arise from selective pathway control, a thermodynamic sink, restricted observability, or another fixed constraint. Matched controls, time-resolved data, or mechanistic information are therefore required. Extending the framework to these systems is a direction for future work.

## Conclusions

AT provides a quantitative framework for comparing undirected and directed exploration in chemical systems. In peptide-forming reactions, exploration ratio and ensemble assembly distinguish nonspecific activation conditions from sequence-selective protease conditions. Undirected reference systems typically yield ER values from 0.77 to 0.96, whereas protease-directed systems typically yield values from 0.51 to 0.75 and higher A per unique product. Bigram analysis confirms that the proteases impose coordinated multiresidue selectivity consistent with their substrate preferences. These results establish experimentally accessible comparative observables for directedness in open chemical systems. When combined with time-resolved evidence or information about how a constraint arises and persists, the same framework can be used to analyze transitions associated with selection. The approach should be useful for reaction-network analysis, complex-mixture chemistry, and the guided discovery of functional molecules in large chemical spaces.

## Supplementary Material



## Data Availability

Source data, including the experimental and simulated data sets used for the main-text and Supporting Information figures, are available on Zenodo and at https://github.com/croningp/peptide_selection. The tandem mass spectra are available from the corresponding author on request because of their size. The code for calculating assembly indices and pathways for experimentally observed sequences was published previously^26^ and is available at https://github.com/croningp/assembly_go. Simulated sequences were analyzed using the assembly pathway implementation described in ref 28, available at https://github.com/croningp/assemblycpp-v5. Code developed for this study is available at https://github.com/croningp/peptide_selection under the MIT license. It generates JAS representations, calculates ER, ensemble assembly, and diversity, and produces the figures. Analyses used Python 3.12 with NetworkX 2.8.8; the theoretical simulations used Wolfram Mathematica 14.

## Notes

Additional experimental details; assembly calculations; computational-model definitions, parameters, replicate statistics, and sensitivity analyses; reaction protocols and XDL examples; statistical analyses and visualizations for all experimental conditions; HPLC and MS/MS acquisition and processing methods; the copy-number sensitivity analysis (PDF)
