# Supplementary material for: Quantifying Directedness in Chemical Reaction Networks Using Assembly Theory
Source: ACS Cent Sci. 2026 Sep 30;12(10):1601–9. doi: 10.1021/acscentsci.6c00618 (PMC13720923; doi:10.1021/acscentsci.6c00618)
Supplement: Supplementary file 1 [file oc6c00618_si_001.pdf]

Supporting Information for

Quantifying Directedness in Chemical Reaction Networks Using Assembly Theory

Michael Jirasek, Abhishek Sharma, Mary Wong, Jennifer Munro, Leroy Cronin*

*lee.cronin@glasgow.ac.uk

School of Chemistry, The University of Glasgow, University Avenue, Glasgow G12 8QQ, UK.

Contents

1 Assembly calculations. 3
1.1 Joint Assembly Space calculations 3
1.2 Computational Model of selection-driven sequential assembly 4
2 Experimental Details – Reactions 7
2.1 General Information 7
2.2 Reactions in Solid Phase. 7
2.2.1 Directed exploration facilitated by proteases 7
2.2.2 Undirected exploration via solid-solid reaction with CDI 8
2.3 CDI in aqueous conditions 8
2.3.1 XDL examples 9
2.4 Wet-Dry Cycling 12
3 Statistical analysis of observed objects and visualisations. 17
3.1 Wet-dry cycling 18
3.1.1 Amino acids G, A, V, H, P 18
3.1.2 Amino acids C, G, A, E, P 22
3.1.3 Amino acids F, G, A, E, P 24
3.1.4 Amino acids F, H, G, A, E 26
3.1.5 Amino acids H, G, A, E, P 28
3.1.6 Amino acids W, G, A, E, P 30
3.1.7 Amino acids G, D, H, S 32
3.2 CDI in solution 35
3.3 CDI in solid state 39
3.4 Catalysis with proteases 41
3.4.1 Cystein proteases 41

3.4.2 Serine proteases 44
3.5 CDI in solution of uneven ratios 46
3.5.1 [G, A, V, H, P] of ratio [31:17:18:33:1] 46
3.5.2 [G, A, V, H, P] of ratio [32:16:15:31:6] 50
3.5.3 [G, A, V, H, P] of ratio [23:39:4:21:13] 54
3.5.4 Exploring various ratios of amino acids 58
4 Experimental Details – Analytical Techniques (HPLC-MS/MS) 59
4.1 Chromatography 59
4.2 MS/MS method 59
4.3 MS/MS Processing 60
4.4 Copy number sensitivity analysis 61
5 References 63

1 Assembly calculations.

Assembly-theoretic calculations were performed for both the experimentally observed peptide sequences and the sequences generated in the computational model. Strings were treated as assembly objects, with individual characters representing the basic building blocks. For the experimental datasets, shortest assembly pathways and molecular assembly indices were calculated using *AssemblyGo*, which is published implementation of a search algorithm for shortest assembly path.[2] For some computational datasets, which contained substantially larger numbers of sequences and much longer strings, assembly indices were calculated using a fast C++ implementation optimised for long-sequence analysis (to be published separately in future, with code avaible on request). The resulting pathways were then combined to approximate the Joint Assembly Space and calculate the corresponding assembly metrics.

1.1 Joint Assembly Space calculations

The Joint Assembly Space (JAS) is a concept in Assembly Theory that represents the combined shortest construction pathways of multiple objects.[1] The JAS calculation was implemented using Python with several key libraries: *NetworkX* for graph representation and manipulation of assembly pathways, *NumPy* for numerical operations and array manipulations, *Pandas* for managing data structures and *iGraph* for additional graph analysis capabilities. The source code can be found in https://github.com/croningp/peptide_selection. The calculation was managed through the *StringToPaths* class with methods to handle timeout conditions and parallel processing using 64 workers by default. The output from *AssemblyGo* was parsed using the *split_pathway_to_nodes_and_edge* method. For each sequence, this generated node lists and edge lists representing the construction pathway. The molecular assembly (MA) index was extracted, representing the minimum number of steps required to construct the sequence.

The JAS was approximated as the union of individual pathways using a *compose_all* method to create a unified directed graph representing an approximation of the JAS. This process was implemented in the *get_graph* static method in the *pathway_helper.py* file. From the unified graph, several metrics were calculated: *Complexity* measured as the number of edges in the graph, *Diversity* as the number of unique sequences, Ensemble assembly *A* as the mean of exponential of assembly indices, *Maximum MA* as the maximum assembly index in the dataset, and *Exploration Ratio* as the key metric defined as the ratio of observed nodes to all nodes in the JAS. The JAS calculation creates a directed graph where nodes represent intermediate constructs and final sequences, edges represent construction steps, and the graph structure captures all possible assembly pathways. The exploration ratio, calculated as the number of observed nodes divided by the number of all nodes, served as the primary metric for quantifying the degree of selection in the system. For each experimental condition and timepoint, the process involved calculating assembly paths for all observed peptide sequences, combining these paths into a unified

graph structure, calculating metrics including the exploration ratio, and comparing these metrics across different experimental conditions to quantify selection. This methodology allowed us to distinguish between directed exploration (with proteases) and undirected exploration (with CDI or heat), with the exploration ratio serving as the quantitative measure of selection.

1.2 Computational Model of selection-driven sequential assembly

In this section, inspired by experimental methodology, we create a simple phenomenological model to represent open-ended exploration in an undirected and directed manner. We simulate a closed, well-mixed population of objects represented by finite strings (sequences) built from a small alphabet of monomers that grow by repeated pairwise ligation (concatenation) under a selective pressure favouring objects that carry a specified terminal motif. The dynamics is a stochastic, mass-conserving kinetic Monte Carlo scheme: at each step two objects are chosen with probabilities proportional to their abundance and selective weight, removed from the pool, and their concatenation is returned to it. Each run yields the complete list of distinct objects that appear over the run; these object lists are the input to the subsequent assembly-theoretic analysis.

The alphabet is the set of five monomers $\{a, b, c, d, e\}$. All the object forms are finite ordered strings, and the system state is the collection of distinct objects currently present together with their non-negative integer copy numbers (populations). Each simulation run is initialised with the five monomers only, each at copy number $P_0 = 10^6$, giving an initial total of $5P_0$ individuals (equivalently, 5×10^6 monomer units). Once an object first appears, it is retained in the record list for the rest of the run, even if its population later returns to zero.

Every object i carries a selection weight

$$w(i) = n(i)\beta(i),\ \beta(i) = s \text{ if object } s \text{ starts or ends with “b”, otherwise } 1,$$

where $n(i)$ is the current population of object i and s is the selective factor. The undirected case is $s = 1$ (all objects equally reactive); $s > 1$ preferentially selects b-terminated objects. The boost depends only on the two terminal monomers of an object and is therefore fixed for that object’s lifetime.

Only objects with positive population participate. A single step proceeds as follows:

- Draw the first reactant a with probability $w(a)/\sum w$, i.e. proportional to abundance × boost.
- Draw the second reactant b proportionally to the same weights, but with one copy of a withheld: its weight reduced to $(n(a) - 1)\beta(a)$ so the two reactants are distinct individuals. b may still be the same object type as a when a has two or more copies.
- Form the product $c = a \oplus b$ by concatenation.

- Update populations: consume one copy of each reactant, $n(a) \rightarrow n(a) - 1$ and $n(b) \rightarrow n(b) - 1$ (if an object selected as both reactants therefore loses two copies), and add one copy of the product, $n(c) \rightarrow n(c) + 1$, creating c if it did not previously exist.

The step is repeated for $N = 10^6$ iterations, and each simulation was repeated 16 times.

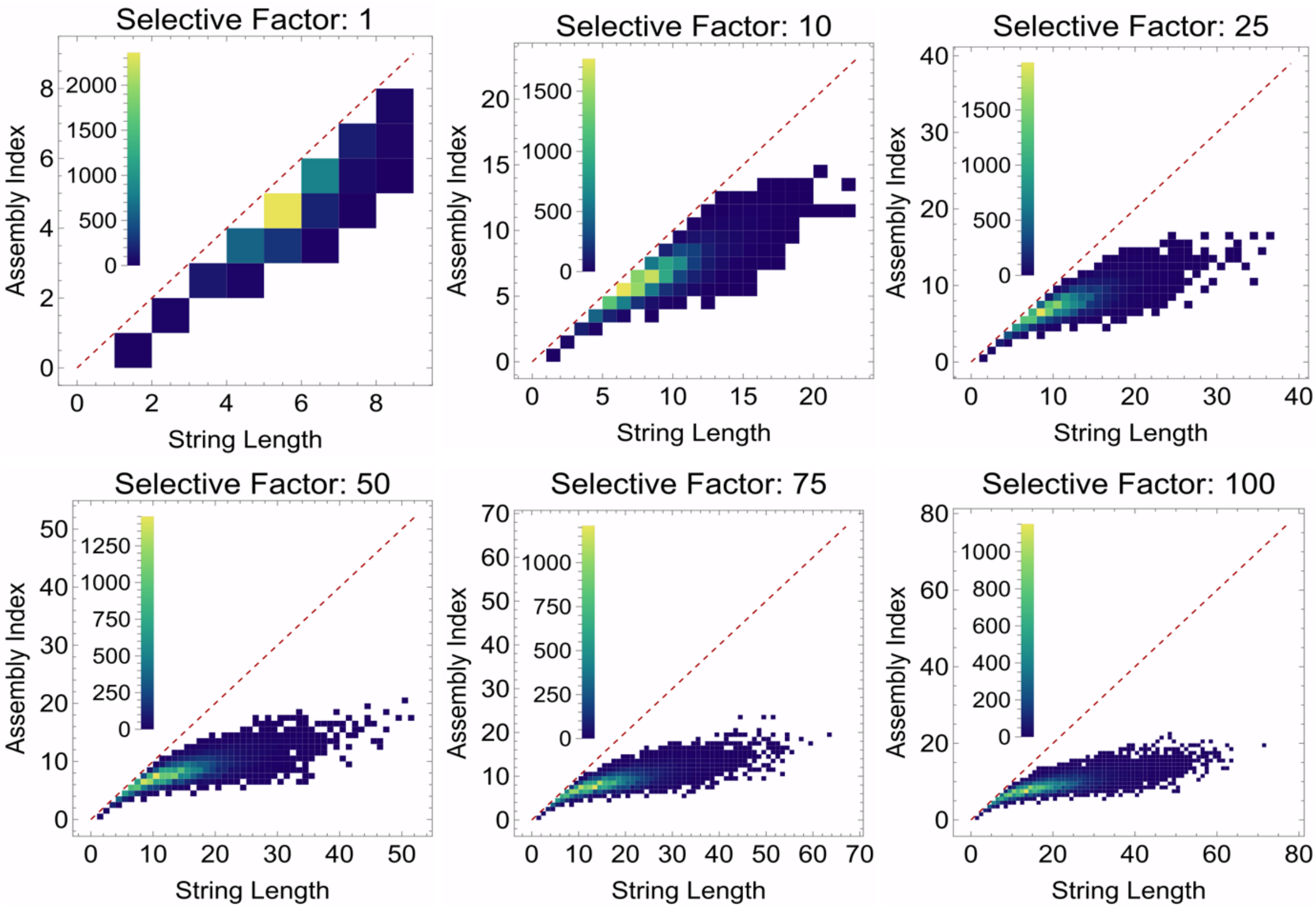

Figure S1. Density histograms of assembly index vs. string length of the observed sequences in a single simulation at different selective factors $s = \{1, 10, 25, 50, 75, 100\}$.

The simulations were performed in Mathematica (Wolfram Ltd) at six different selective factors $s = \{1, 10, 25, 50, 75, 100\}$. In principle, it is possible to expand beyond selective factor 100, which will lead to the formation of very long strings. **Figure S1** shows the density histograms at different selective factors. Additionally, we estimated the similarity in the observed sequences between multiple runs by estimating the average of intersection-over-union across all pairwise combinations. The potential space is nearly completely explored among all independent runs at lower assembly indices, and similarity falls with increasing assembly indices. This is due to an increase in the large combinatorial space of potential sequences with assembly index, where the occurrence of similar sequences among multiple runs falls, see **Figure S2**.

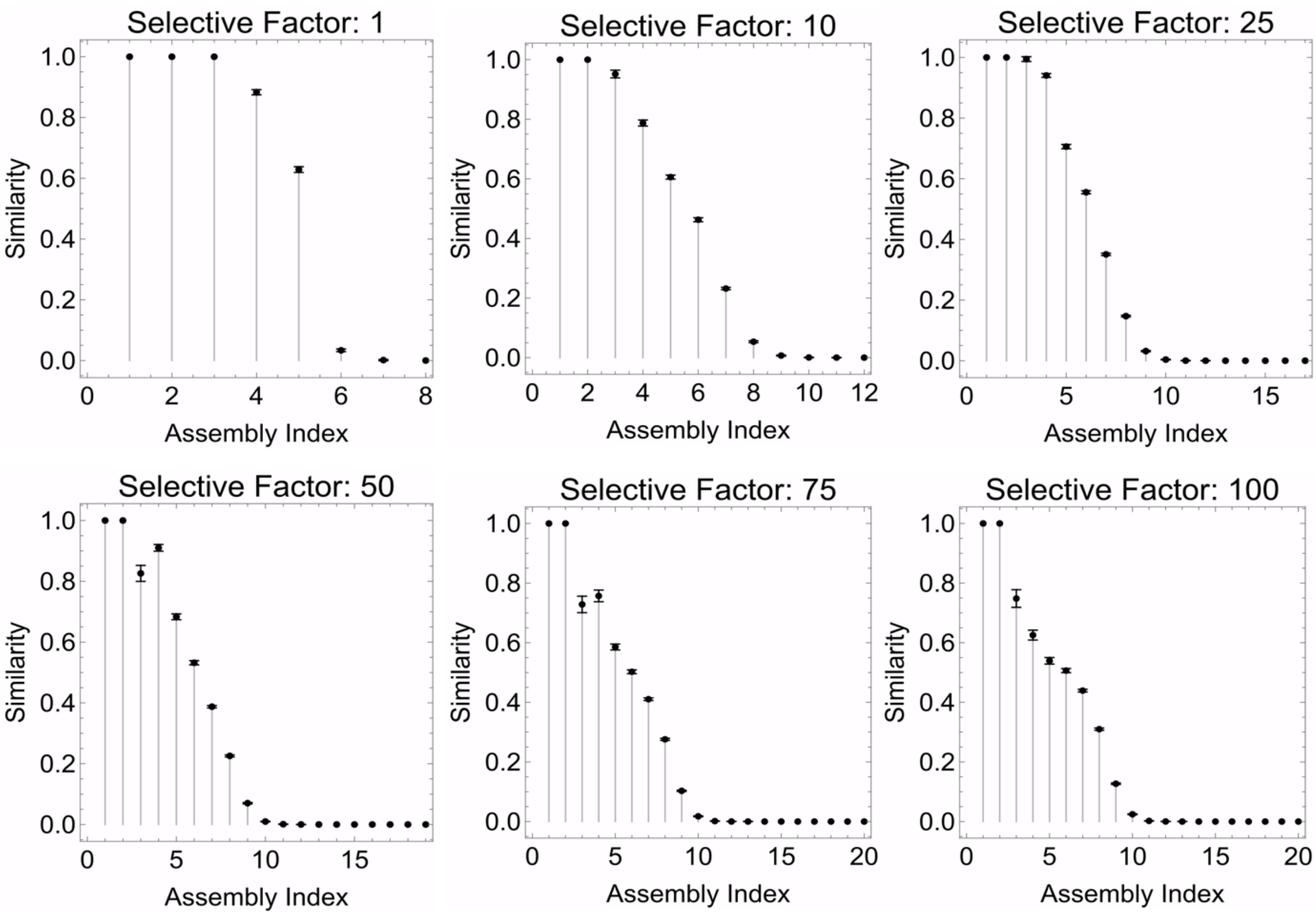

Figure S2. Similarity distributions among the observed sequences at different assembly indices at different selective factors $s = \{1, 10, 25, 50, 75, 100\}$ over 16 runs.

2 Experimental Details – Reactions

2.1 General Information

Procedure to facilitate amino acid polymerisation involved combining amino acids and providing conditions for the polymerisation such as heat or coupling agent.

Reagents. CDI was purchased from Merck in reagent grade quality. L-Valine, L-ornithine monohydrochloride, L-aspartic acid, glycine, L-alanine, L-cysteine, L-valine, L-histidine and L-proline were purchased from Merck with highest grade purity (>99%). Proteases chymotrypsin from bovine pancreas with declared activity 350U/mg; papain from *carica papaya*, with activity 320000 USP-U/mg; bromelain from pineapple stem with >3 units/mg protein; trypsin from porcine pancreas with activity 1000-2000 BAEE units/mg were all purchased from Merck.

2.2 Reactions in Solid Phase.

Solid-state reactions were conducted using a RETSCH MM 200 Model Mixer Mill equipped with two ZrO_2 grinding balls. The mill operated at a frequency of 25 Hz using two jars at the same.

2.2.1 Directed exploration facilitated by proteases

Typical protocol for performing mechanochemical polymerisation of amino acid methyl esters facilitated by proteases were adapted from literature.[3–5] For a typical experiment involving mechanochemical polymerization of amino acid methyl esters with proteases, equimolar amounts of amino acid methyl esters is weighed. When using papain or bromelain, 10 percent by weight of the enzyme relative to the amino acids is added, along with 10 percent L-cysteine (which maintains the catalytic activity of these cysteine proteases) and 10 percent Na_2CO_3. The components are transferred to a reaction vessel, a zirconium oxide milling jar with 2 balls, and agitated at a frequency of 20 Hz for 30 minutes. This mechanical energy facilitates enzymatic peptide bond formation in a solvent-free environment, creating a concentrated reaction medium that promotes polymerization. After the initial milling cycle, approximately 5 milligrams of sample is collected for analysis by HPLC and mass spectrometry. The remaining mixture undergoes additional agitation cycles under identical conditions, with samples taken between cycles to monitor monomer consumption and peptide exploration.

Concrete typical example of procure follows: in the left milling jar, equimolar quantities (0.375 mmol) of amino acid methyl esters were combined: L-alanine methyl ester (52 mg), glycine methyl ester (47 mg), L-histidine methyl ester (91 mg), L-valine methyl ester (63 mg), and L-proline methyl ester (62 mg). The reaction mixture was supplemented with bromelain (325 mg) as the catalyst, Na_2CO_3 (199 mg) as the base, and L-cysteine as a cofactor. Two ZrO_2 balls were added to the jar before sealing. To ensure

balanced operation of the ball mill, the right milling jar was loaded with twice the amount of all reagents. The reaction mixture was mechanically agitated for 30 minutes.

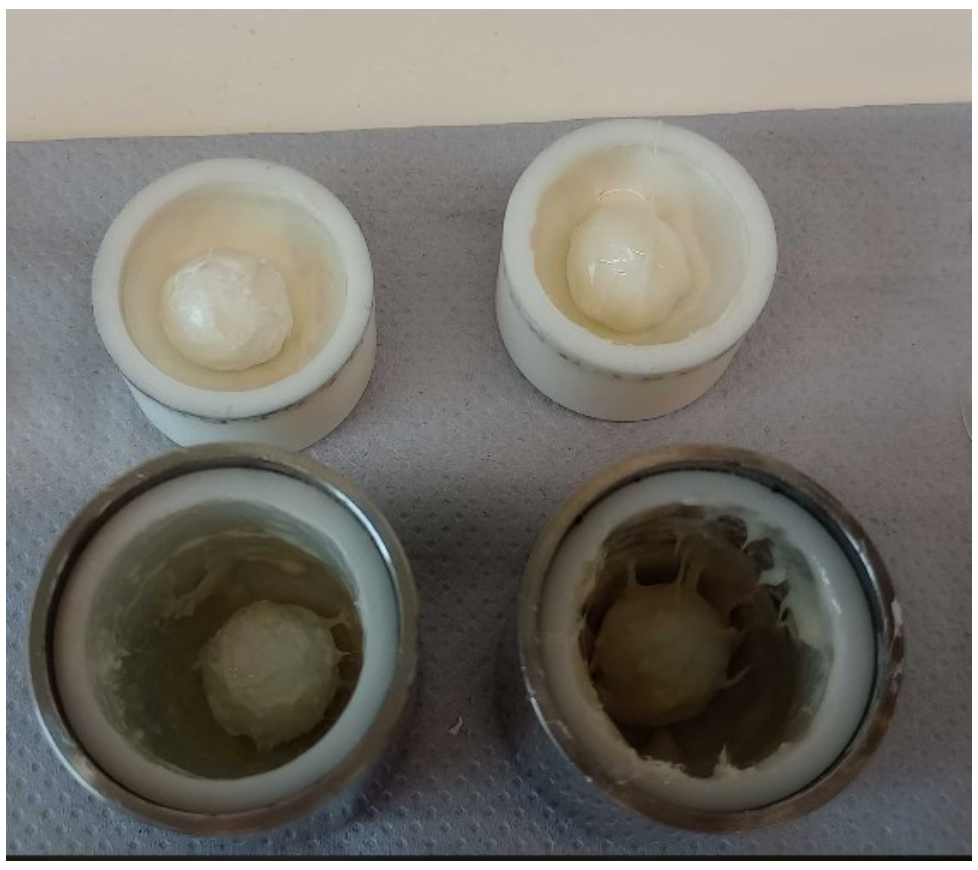

Figure S3. Typical state of the solids in ball mill upon polymerisation facilitated by proteases.

2.2.2 Undirected exploration via solid-solid reaction with CDI

For typical experiment, 1 mmol of each amino acid was weighed in. 0.125 equivalents of solid CDI (20 mg) was added each time. The whole capsule was agitated with frequency 20 Hz for 90 min. Then, sample (~ 5 mg) was taken for analysis, the CDI was readded to repeat the cycle.

2.3 CDI in aqueous conditions

A liquid-handling robot equipped with shaker from QInstruments Bioshake 3000 was used for the reaction. For the three amino acids experiment, stock solutions of L-ornithine **O** (0.05 M), L-valine **V** (0.1 M) and L-aspartic acid **D** (0.05 M) were prepared in deionised water prior the experiment. The equal volumes of the amino acid stocks solutions (1 mL) were transferred to the reaction vial in a heated manifold via pipetting robot Opentrons OT2. Reaction mixture was mixed and heated at 160 °C for 45 minutes. The dried mixture was resuspended by water (2 mL), 50% portion was transferred to storage and mixture was replenished with portions of amino acids. And the cycle was repeated 9 times.
Stock solution of CDI (0.5 M in acetonitrile) was prepared on the same day prior the experiment. For the five amino acids experiments, stock solutions of glycine **G** (0.2 M), L-alanine **A** (0.2 M), L-valine **V** (0.2 M), L-histidine **H** (0.2 M) and L-proline P (0.2 M) were prepared fresh on the day of the experiments. Equal amounts (1 mL) of amino acid stock solutions were transferred to reactor vial via pipetting robot Opentrons OT2. The vials were shaken for 10 seconds, and a portion of CDI stock solution was added (0.7 mL). The mixture was allowed to react at elevated temperature 55 °C for 2 h, after that allow to cool to room temperature over 1.5 h. Portion of 15 % of the reaction mixture was transferred to storage vial and the volume was replenished with amino acids and portion of the CDI (0.7 mL) was added. This cycle has been repeated 10 times.

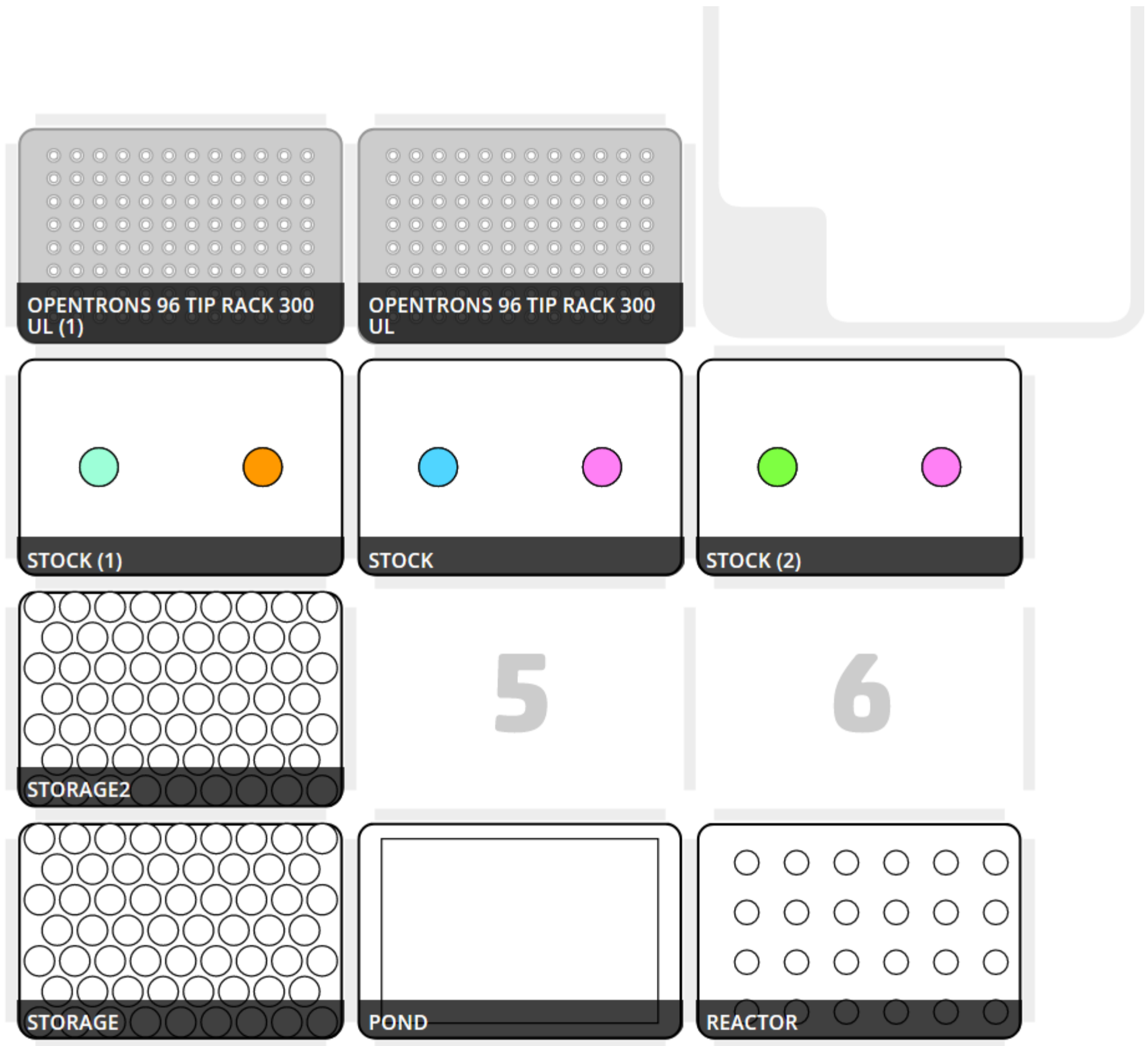

Figure S4. Typical graph layout of Opentrons OT2 for the amino acid polymerisation in liquids.

2.3.1 XDL examples

A snippet of a typical XDL representing the amino acid reaction polymerisation with CDI solution followed up by heating at 40°C. The XDL files are available in the project folder on GitHub.

```
<XDL>
    <Synthesis>
        <Hardware>
            <Component id="pond_A1" type="storage" />
            <Component id="reactor_A1" type="reactor" />
            ...
            <Component id="reactor_D6" type="reactor" />

            <Component id="storage_A1" type="storage" />
            ...
            <Component id="storage_G9" type="storage" />
            <Component id="storage2_A1" type="storage" />
            ...
            <Component id="storage2_A2" type="storage" />
            ...
            <Component id="storage2_G9" type="storage" />
        </Hardware>

        <Reagents>
            <Reagent name="G" comment="c = 200 mM" role="reagent" />
            <Reagent name="A" comment="c = 200 mM" role="reagent" />
            <Reagent name="V" comment="c = 200 mM" role="reagent" />
            <Reagent name="H" comment="c = 200 mM" role="reagent" />
            <Reagent name="P" comment="c = 200 mM" role="reagent" />
            <Reagent name="CDI" comment="c = 500 mM" />
        </Reagents>

        <Procedure>
            <Add reagent="G" vessel="reactor_A1" volume="1.0 mL" />
            <Add reagent="G" vessel="reactor_A2" volume="1.0 mL" />
            <Add reagent="G" vessel="reactor_A3" volume="1.0 mL" />
            <Add reagent="G" vessel="reactor_A4" volume="1.0 mL" />
            <Add reagent="A" vessel="reactor_A1" volume="1.0 mL" />
            <Add reagent="A" vessel="reactor_A2" volume="1.0 mL" />
            ...
            <Add reagent="P" vessel="reactor_A3" volume="1.0 mL" />
            <Add reagent="P" vessel="reactor_A4" volume="1.0 mL" />

            <Stir vessel="reactor_A1" time="10 s" stir_speed="800" />

            <Add reagent="CDI" vessel="reactor_A1" volume="0.7 mL" />
            <Add reagent="CDI" vessel="reactor_A2" volume="0.7 mL" />
            <Add reagent="CDI" vessel="reactor_A3" volume="0.7 mL" />
            <Add reagent="CDI" vessel="reactor_A4" volume="0.7 mL" />
            <Stir vessel="reactor_A1" time="10 s" stir_speed="800" />
            <ODropTip pipette="left" location="None" home_after="False" />

            <HeatChill vessel="reactor_A1" temp="55" time="120 min"
                upper_temp_tolerance="4.0" lower_temp_tolerance="4.0"
                stir="True" stir_speed="800" />
            <Wait time="90 min" />
```
Figure S5. Snippet of XDL code for CDI-facilitated polymerisation experiments (part 1/2).

```
        <Transfer from_vessel="reactor_A1" to_vessel="storage_A1" volume="0.727 mL" />
        <Transfer from_vessel="reactor_A2" to_vessel="storage_A2" volume="0.727 mL" />
        <Transfer from_vessel="reactor_A3" to_vessel="storage_A3" volume="0.727 mL" />
        <Transfer from_vessel="reactor_A4" to_vessel="storage_A4" volume="0.727 mL" />

        <Add reagent="G" vessel="reactor_A1" volume="0.15 mL" />
        <Add reagent="G" vessel="reactor_A2" volume="0.15 mL" />
        <Add reagent="G" vessel="reactor_A3" volume="0.15 mL" />
        <Add reagent="G" vessel="reactor_A4" volume="0.15 mL" />
        <Add reagent="A" vessel="reactor_A1" volume="0.15 mL" />
        <Add reagent="A" vessel="reactor_A2" volume="0.15 mL" />
        <Add reagent="A" vessel="reactor_A3" volume="0.15 mL" />
        <Add reagent="A" vessel="reactor_A4" volume="0.15 mL" />
        <Add reagent="V" vessel="reactor_A1" volume="0.15 mL" />
        <Add reagent="V" vessel="reactor_A2" volume="0.15 mL" />
        <Add reagent="V" vessel="reactor_A3" volume="0.15 mL" />
        <Add reagent="V" vessel="reactor_A4" volume="0.15 mL" />
        <Add reagent="H" vessel="reactor_A1" volume="0.15 mL" />
        <Add reagent="H" vessel="reactor_A2" volume="0.15 mL" />
        <Add reagent="H" vessel="reactor_A3" volume="0.15 mL" />
        <Add reagent="H" vessel="reactor_A4" volume="0.15 mL" />
        <Add reagent="P" vessel="reactor_A1" volume="0.15 mL" />
        <Add reagent="P" vessel="reactor_A2" volume="0.15 mL" />
        <Add reagent="P" vessel="reactor_A3" volume="0.15 mL" />
        <Add reagent="P" vessel="reactor_A4" volume="0.15 mL" />

        <Add reagent="CDI" vessel="reactor_A1" volume="0.7 mL" />
        <Add reagent="CDI" vessel="reactor_A2" volume="0.7 mL" />
        <Add reagent="CDI" vessel="reactor_A3" volume="0.7 mL" />
        <Add reagent="CDI" vessel="reactor_A4" volume="0.7 mL" />
        <Stir vessel="reactor_A1" time="10 s" stir_speed="800" />
        <ODropTip pipette="left" location="None" home_after="False" />

        <HeatChill vessel="reactor_A1" temp="55" time="120 min"
            upper_temp_tolerance="4.0" lower_temp_tolerance="4.0"
            stir="True" stir_speed="800" />
        <Wait time="90 min" />
```

```
            <Transfer from_vessel="reactor_A1" to_vessel="storage_E7" volume="0.669 mL" />
            <Transfer from_vessel="reactor_A2" to_vessel="storage_E8" volume="0.669 mL" />
            <Transfer from_vessel="reactor_A3" to_vessel="storage_E9" volume="0.669 mL" />
            <Transfer from_vessel="reactor_A4" to_vessel="storage_F1" volume="0.669 mL" />

        </Procedure>
    </Synthesis>
</XDL>
```

Figure S6. Snippet of XDL code for CDI-facilitated polymerisation experiments (part 2/2).

2.4 Wet-Dry Cycling

To facilitate polymerisation with heat, we used liquid-handling robot with shaker from QInstruments Bioshake 3000 with a custom heating block setup to reach high temperatures. The heater part was built on top of the Bioshake shaker, made from customised CNC machined block hosting 7 mL vials, two heating rods and a thermocouple for temperature control. The heating and temperature control was facilitated by Arduino MEGA microcontroller (**Figure S7**).

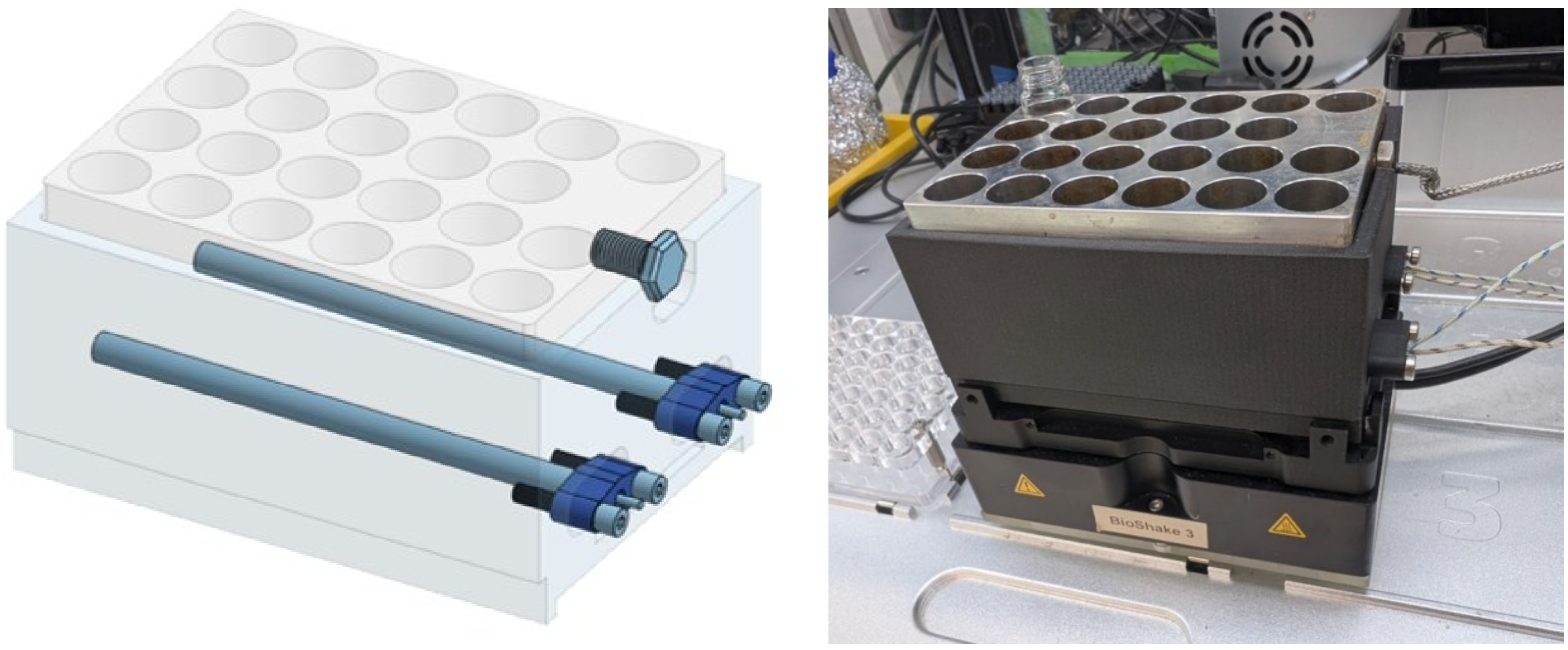

Figure S7. (left) CAD design of aluminium heater block with two heating rods and thermocouple screw. (right) Photo of the heater/shaker prototype.

The reaction was carried out by combining amino acid stock solutions. Heating up to high temperature, typically in most cases 130 degrees, the heater was heating for 4 hours. The heating was turned off and waiting period of 40 min was applied. Then rehydrating them with water, removing a portion of the solution depending on the set recursive amount (typically 50 percent), using this for HPLC analysis. The remaining part was replenished with amino acid solutions. The experiment was repeated (this is one cycle). Typical layout of experiment is depicted in **Figure S8**.

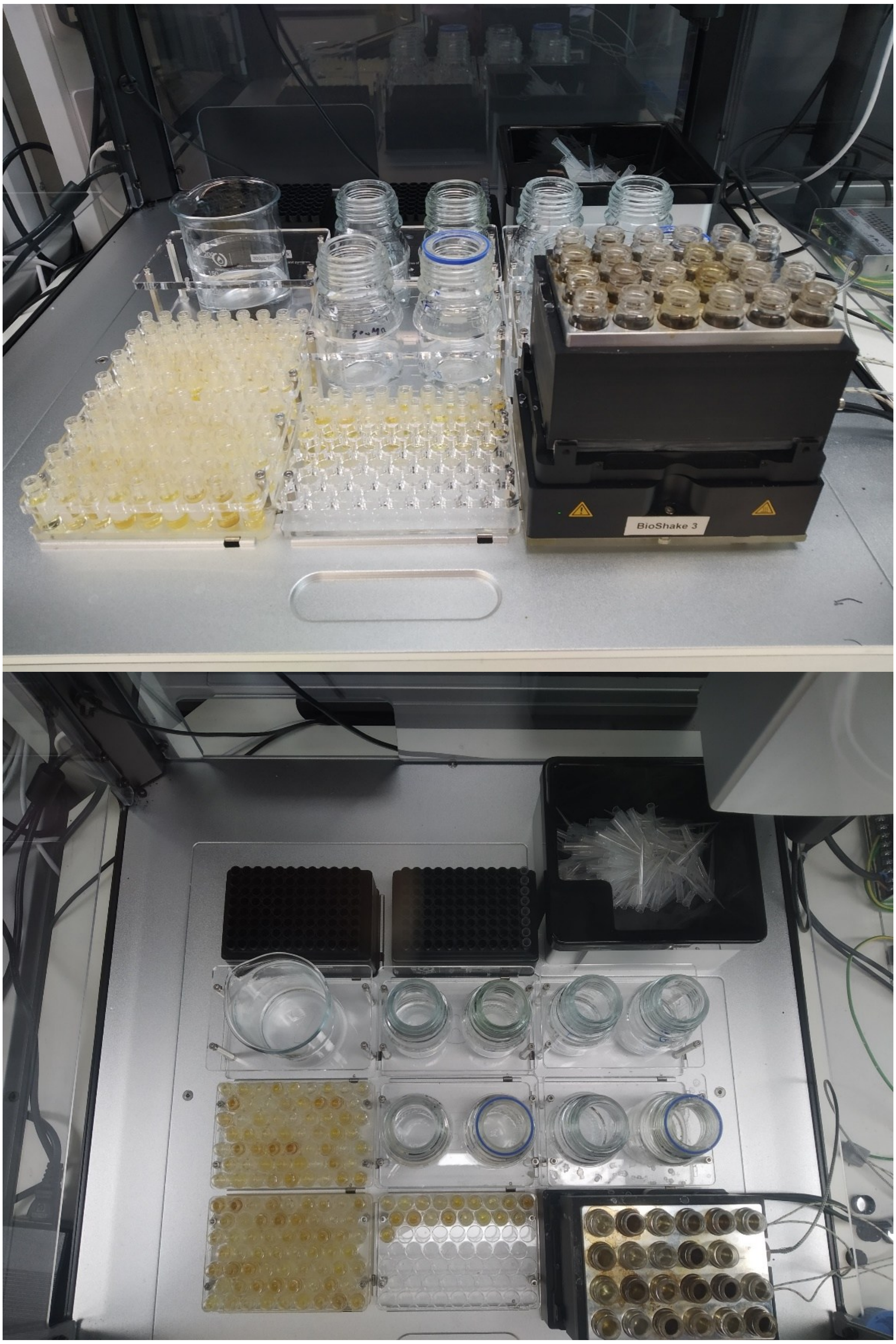

Figure S8. Photo of layout of labware for wet dry cycling experiments. Stocks of amino acids are stored in large 100 mL Duran bottles, reaction performed in customised heater shaker with 7 mL vials and samples stored each cycle in the HPLC vials.

A snippet of a typical XDL representing the wet-dry cycling consisting of 4 hours of heat at 130°C, followed by water addition, sample resuspension, taking some portion away as sample or to waste (pond) and running the experiment again. The XDL files are available in the project folder on GitHub.

```
<XDL>
    <Synthesis>
        <Hardware>
            <Component id="reactor_A1" type="reactor" />
            <Component id="reactor_A2" type="reactor" />
            <Component id="reactor_A3" type="reactor" />
            <Component id="pond_A1" type="reactor" />

            <Component id="STORAGE1_A1" type="storage" />
            <Component id="STORAGE1_A2" type="storage" />
            <Component id="STORAGE1_A3" type="storage" />
            ...
            <Component id="STORAGE1_G9" type="storage" />
        </Hardware>

        <Reagents>
            <Reagent name="G" comment="c = 200 mM" role="reagent" />
            <Reagent name="A" comment="c = 200 mM" role="reagent" />
            <Reagent name="V" comment="c = 200 mM" role="reagent" />
            <Reagent name="H" comment="c = 200 mM" role="reagent" />
            <Reagent name="P" comment="c = 200 mM" role="reagent" />
            <Reagent name="Water" role="reagent" />
        </Reagents>

        <Procedure>
            <Add reagent="G" vessel="reactor_A1" volume="0.8 mL" />
            <Add reagent="G" vessel="reactor_A2" volume="0.8 mL" />
            <Add reagent="G" vessel="reactor_A3" volume="0.8 mL" />
            <Add reagent="A" vessel="reactor_A1" volume="0.8 mL" />
            <Add reagent="A" vessel="reactor_A2" volume="0.8 mL" />
            <Add reagent="A" vessel="reactor_A3" volume="0.8 mL" />
            <Add reagent="V" vessel="reactor_A1" volume="0.8 mL" />
            <Add reagent="V" vessel="reactor_A2" volume="0.8 mL" />
            <Add reagent="V" vessel="reactor_A3" volume="0.8 mL" />
            <Add reagent="H" vessel="reactor_A1" volume="0.8 mL" />
            <Add reagent="H" vessel="reactor_A2" volume="0.8 mL" />
            <Add reagent="H" vessel="reactor_A3" volume="0.8 mL" />
            <Add reagent="P" vessel="reactor_A1" volume="0.8 mL" />
            <Add reagent="P" vessel="reactor_A2" volume="0.8 mL" />
            <Add reagent="P" vessel="reactor_A3" volume="0.8 mL" />

            <HeatChill temp="130" vessel="reactor_A1" time="240 min" stir_speed="400" />
            <Wait time="40 min" />
```

Figure S9. Snippet of XDL code for wet-dry cycling experiments (part 1/3).

```
<Add reagent="Water" vessel="reactor_A1" volume="4.0 mL" />
<Add reagent="Water" vessel="reactor_A2" volume="4.0 mL" />
<Add reagent="Water" vessel="reactor_A3" volume="4.0 mL" />
<Stir vessel="reactor_A1" time="5 min" stir_speed="400" />

<Transfer from_vessel="reactor_A1" to_vessel="pond_A1" volume="1.6 mL" />
<Transfer from_vessel="reactor_A1" to_vessel="STORAGE1_A1" volume="1.8 mL" />
<ODropTip pipette="left" location="None" home_after="False" />
<Transfer from_vessel="reactor_A2" to_vessel="pond_A1" volume="0.2 mL" />
<Transfer from_vessel="reactor_A2" to_vessel="STORAGE1_A2" volume="1.8 mL" />
<ODropTip pipette="left" location="None" home_after="False" />
<Transfer from_vessel="reactor_A3" to_vessel="STORAGE1_A3" volume="0.6 mL" />
<ODropTip pipette="left" location="None" home_after="False" />

<Add reagent="G" vessel="reactor_A1" volume="0.68 mL" />
<Add reagent="G" vessel="reactor_A2" volume="0.4 mL" />
<Add reagent="G" vessel="reactor_A3" volume="0.12 mL" />
<Add reagent="A" vessel="reactor_A1" volume="0.68 mL" />
<Add reagent="A" vessel="reactor_A2" volume="0.4 mL" />
<Add reagent="A" vessel="reactor_A3" volume="0.12 mL" />
<Add reagent="V" vessel="reactor_A1" volume="0.68 mL" />
<Add reagent="V" vessel="reactor_A2" volume="0.4 mL" />
<Add reagent="V" vessel="reactor_A3" volume="0.12 mL" />
<Add reagent="H" vessel="reactor_A1" volume="0.68 mL" />
<Add reagent="H" vessel="reactor_A2" volume="0.4 mL" />
<Add reagent="H" vessel="reactor_A3" volume="0.12 mL" />
<Add reagent="P" vessel="reactor_A1" volume="0.68 mL" />
<Add reagent="P" vessel="reactor_A2" volume="0.4 mL" />
<Add reagent="P" vessel="reactor_A3" volume="0.12 mL" />

<HeatChill temp="130" vessel="reactor_A1" time="240 min" stir_speed="400" />
<Wait time="40 min" />
```

Figure S10. Snippet of XDL code for wet-dry cycling experiments (part 2/3).

```
            <Add reagent="G" vessel="reactor_A1" volume="0.68 mL" />
            <Add reagent="G" vessel="reactor_A2" volume="0.4 mL" />
            <Add reagent="G" vessel="reactor_A3" volume="0.12 mL" />
            <Add reagent="A" vessel="reactor_A1" volume="0.68 mL" />
            <Add reagent="A" vessel="reactor_A2" volume="0.4 mL" />
            <Add reagent="A" vessel="reactor_A3" volume="0.12 mL" />
            <Add reagent="V" vessel="reactor_A1" volume="0.68 mL" />
            <Add reagent="V" vessel="reactor_A2" volume="0.4 mL" />
            <Add reagent="V" vessel="reactor_A3" volume="0.12 mL" />
            <Add reagent="H" vessel="reactor_A1" volume="0.68 mL" />
            <Add reagent="H" vessel="reactor_A2" volume="0.4 mL" />
            <Add reagent="H" vessel="reactor_A3" volume="0.12 mL" />
            <Add reagent="P" vessel="reactor_A1" volume="0.68 mL" />
            <Add reagent="P" vessel="reactor_A2" volume="0.4 mL" />
            <Add reagent="P" vessel="reactor_A3" volume="0.12 mL" />

            <HeatChill vessel="reactor_A1" temp="130" time="240 min" stir_speed="400" />
            <Wait time="40 min" />

            <Add reagent="Water" vessel="reactor_A1" volume="4.0 mL" />
            <Add reagent="Water" vessel="reactor_A2" volume="4.0 mL" />
            <Add reagent="Water" vessel="reactor_A3" volume="4.0 mL" />
            <Stir vessel="reactor_A1" time="5 min" stir_speed="400" />

            <Transfer from_vessel="reactor_A1" to_vessel="pond_A1" volume="1.6 mL" />
            <Transfer from_vessel="reactor_A1" to_vessel="STORAGE1_D2" volume="1.8 mL" />
            <ODropTip pipette="left" location="None" home_after="False" />
            <Transfer from_vessel="reactor_A2" to_vessel="pond_A1" volume="0.2 mL" />
            <Transfer from_vessel="reactor_A2" to_vessel="STORAGE1_D3" volume="1.8 mL" />
            <ODropTip pipette="left" location="None" home_after="False" />
            <Transfer from_vessel="reactor_A3" to_vessel="STORAGE1_D4" volume="0.6 mL" />
            <ODropTip pipette="left" location="None" home_after="False" />

        </Procedure>
    </Synthesis>
</XDL>
```

Figure S11. Snippet of XDL code for wet-dry cycling experiments (part 3/3).

3 Statistical analysis of observed objects and visualisations.

Understanding the selectivity of peptide discovery processes requires analysis beyond individual position preferences, as selectivity may manifest as coordinated preferences across multiple amino acid positions. Such multi-position selectivity could contribute to substrate discrimination and help explain sequence patterns observed in different synthesis approaches, providing insights why exploration ratio serves as an effective metric for characterizing degree of selection.

We performed bigram analysis to examine how amino acid sequences are distributed in enzymatically-generated versus randomly coupled peptides. We applied analysis at two levels of complexity to characterize potential selectivity patterns. For single amino acid analysis, the method examines the frequency with which individual amino acids are followed by other individual amino acids within sequences, capturing direct amino acid-to-amino acid transitions (e.g., G→A, A→V) that may reveal local sequence preferences. For multi-residue analysis, the approach was extended to examine transitions between two-amino-acid patterns, treating longer sequences as series of overlapping two-amino-acid transitions. For a sequence ABCD, the bigram transitions become AB→ CD. If multi-position selectivity occurs, the resulting bigra7m matrices should show patterns where certain transitions are enriched while others are depleted relative to random coupling, potentially providing evidence for coordinated amino acid preferences that complement individual position analysis.

3.1 Wet-dry cycling

Wet-dry cycling experiments involved heating amino acid solutions to 130 °C for 4 hours using a custom aluminium heating block equipped with temperature control, followed by cooling and rehydration with water. After each heating cycle, 50% of the reaction mixture was removed for HPLC-MS/MS analysis and the remaining volume was replenished with fresh amino acid stock solutions to maintain consistent starting concentrations. The high-temperature dehydration promotes peptide bond formation through condensation reactions while the rehydration step allows for product dissolution and sampling. Multiple amino acid combinations were tested including the standard set (G, A, V, H, P) as well as alternative compositions (C, G, A, E, P), (F, G, A, E, P), (F, H, G, A, E), (H, G, A, E, P), (W, G, A, E, P), and (G, D, H, S) to evaluate sequence space exploration across different chemical environments and different number of building blocks. The cycling process was repeated for up to 6-10 iterations per experiment, with samples collected after each cycle to monitor temporal evolution of peptide diversity and molecular assembly indices. This thermal coupling approach eliminates the need for chemical activating agents, relying instead on high-temperature thermodynamics to drive amide bond formation and assess intrinsic amino acid coupling preferences under purely thermal activation conditions.

3.1.1 Amino acids G, A, V, H, P

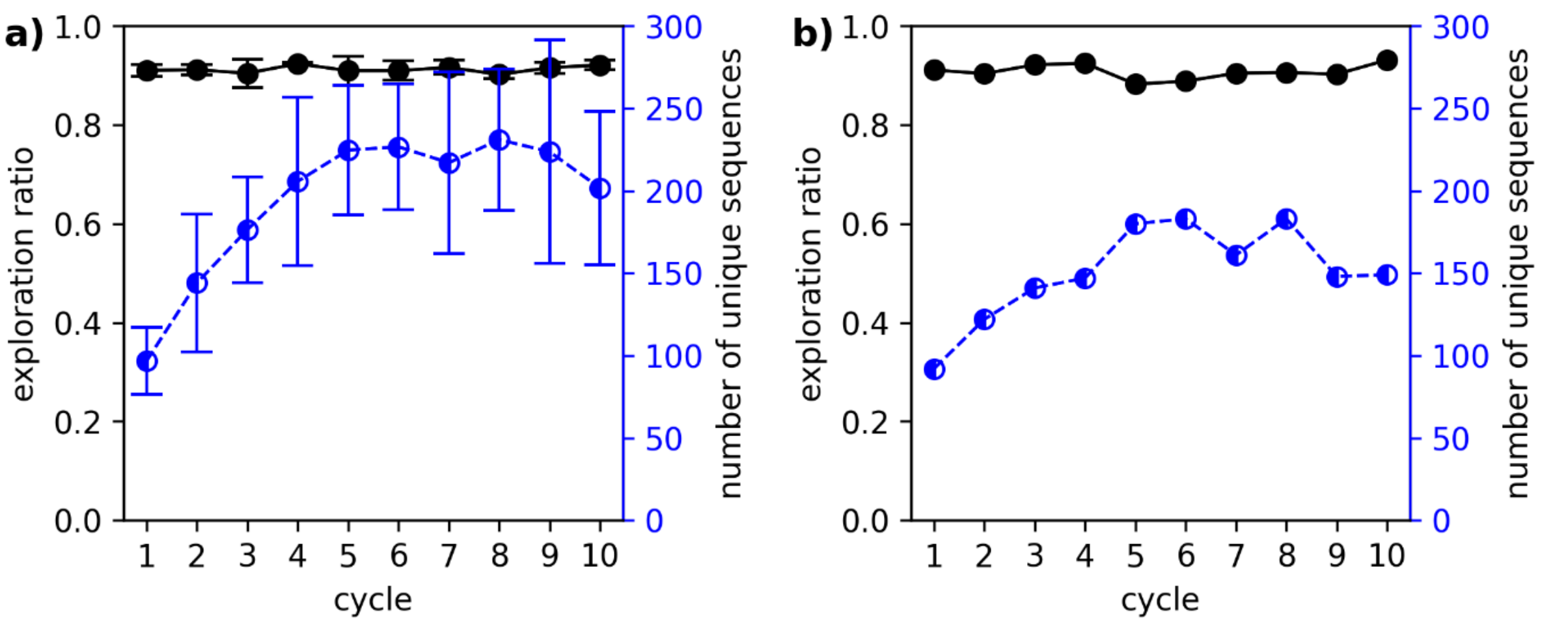

Figure S12. Number of unique peptides (blue) and exploration ratio (black) of peptide ensembles generated by wet-dry cycling of equimolar amounts of G, A, V, H, P at 130 °C for 4h and resuspending again (one cycle). Average values for replicates a) and single trajectory b) used for bigram matrix generations (see bellow).

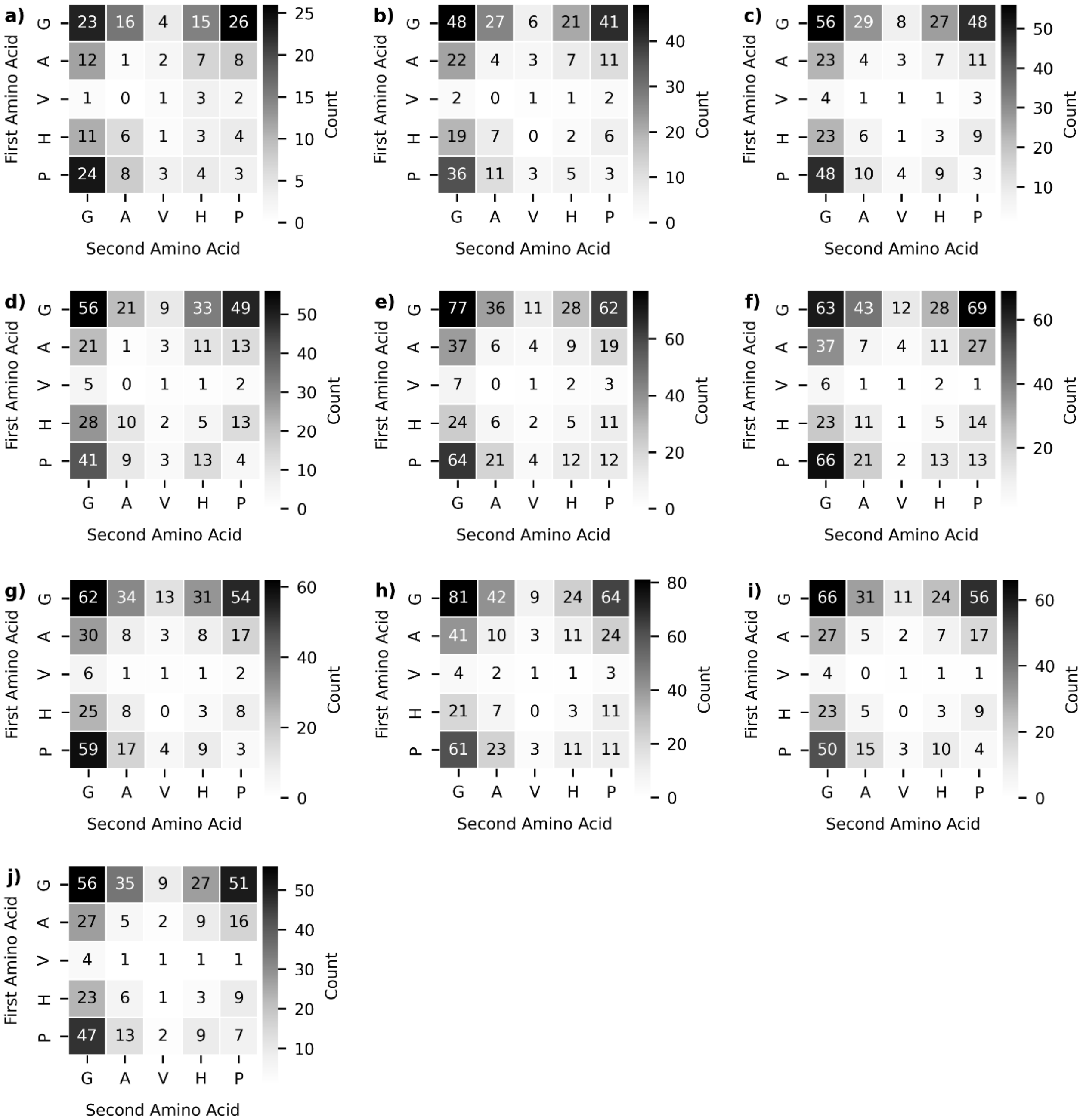

Figure S13. Bigram matrix of 2 amino acid sequence occurrence in observed peptides generated by wet-dry cycling of equimolar amounts of G, A, V, H, P at 130 °C for 4h and resuspending again throughout cycles from a) to j).

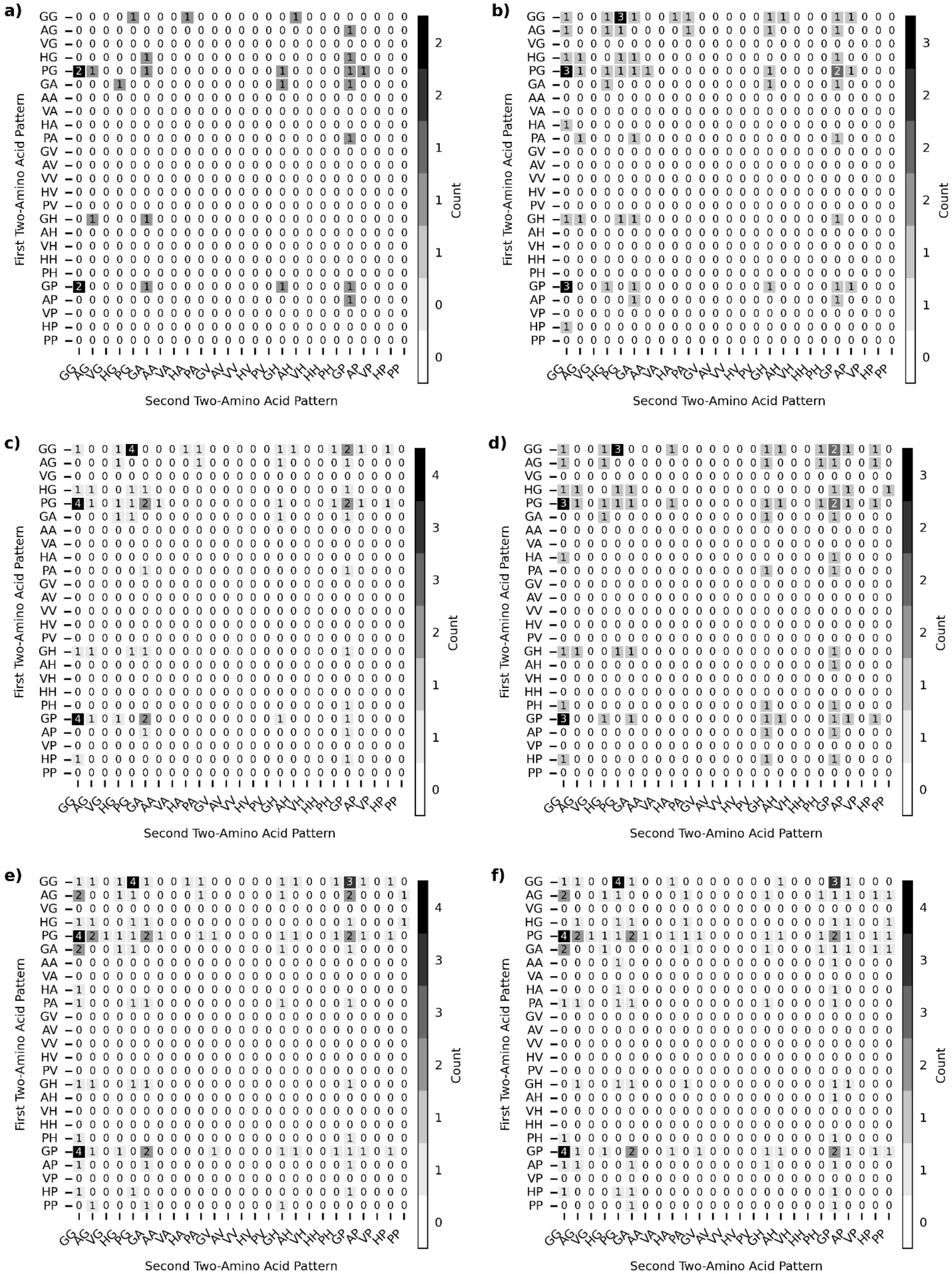
a)
b)
c)
d)
e)
f)
First Two-Amino Acid Pattern
Second Two-Amino Acid Pattern
Count
GG AG VG HG PG GA AA VA HA PA GV AV VV HV PV GH AH VH HH PH GP AP VP HP PP

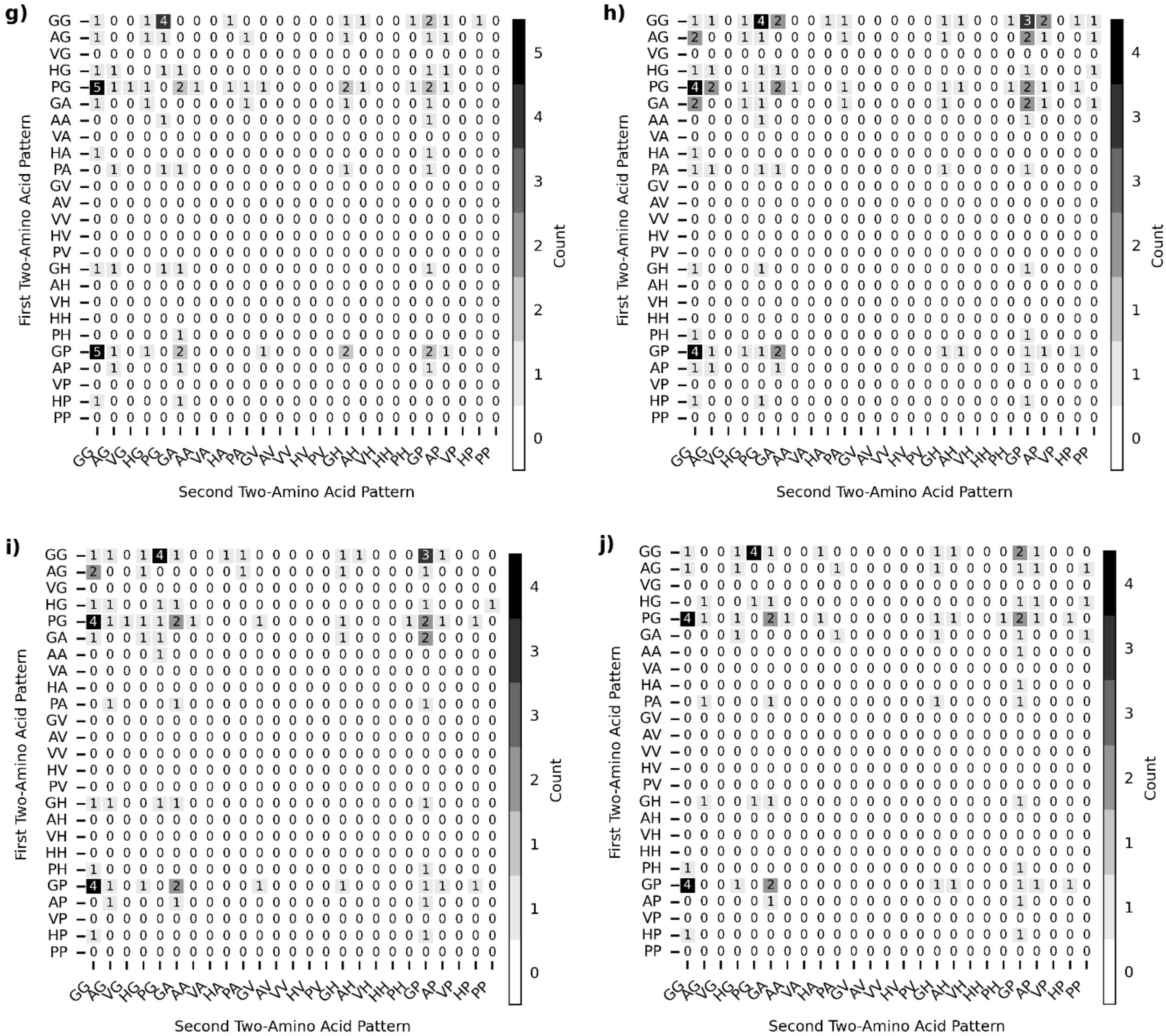

Figure S14 Bigram matrix of 4 amino acid sequence occurrence in observed peptides generated by wet-dry cycling of equimolar amounts of G, A, V, H, P at 130 °C for 4h and resuspending again throughout cycles from a) to j).

3.1.2 Amino acids C, G, A, E, P

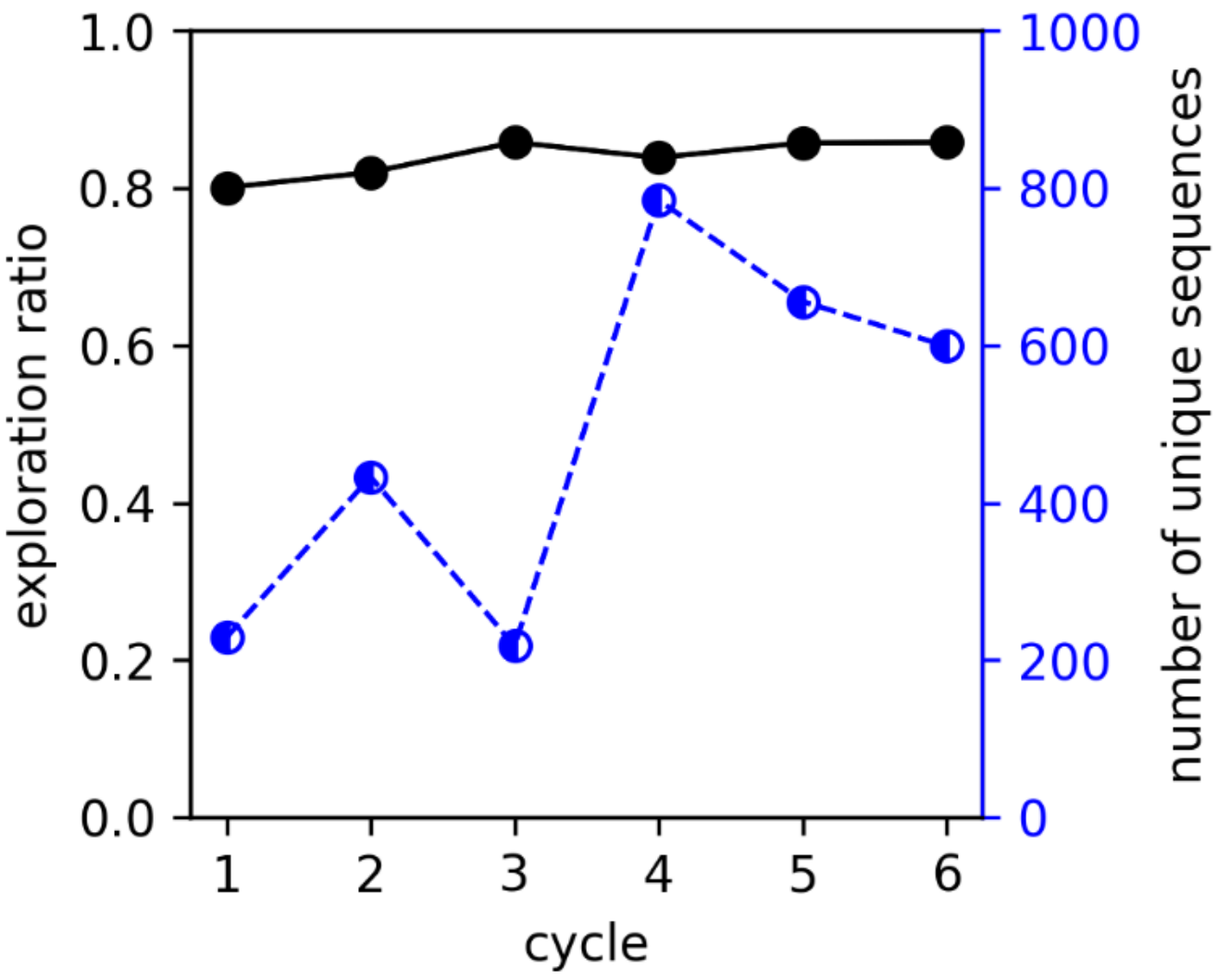

Figure S15. Number of unique peptides (blue) and exploration ratio (black) of peptide ensembles generated by wet-dry cycling of equimolar amounts of C, G, A, E, P at 130 °C for 4h and resuspending again (one cycle).

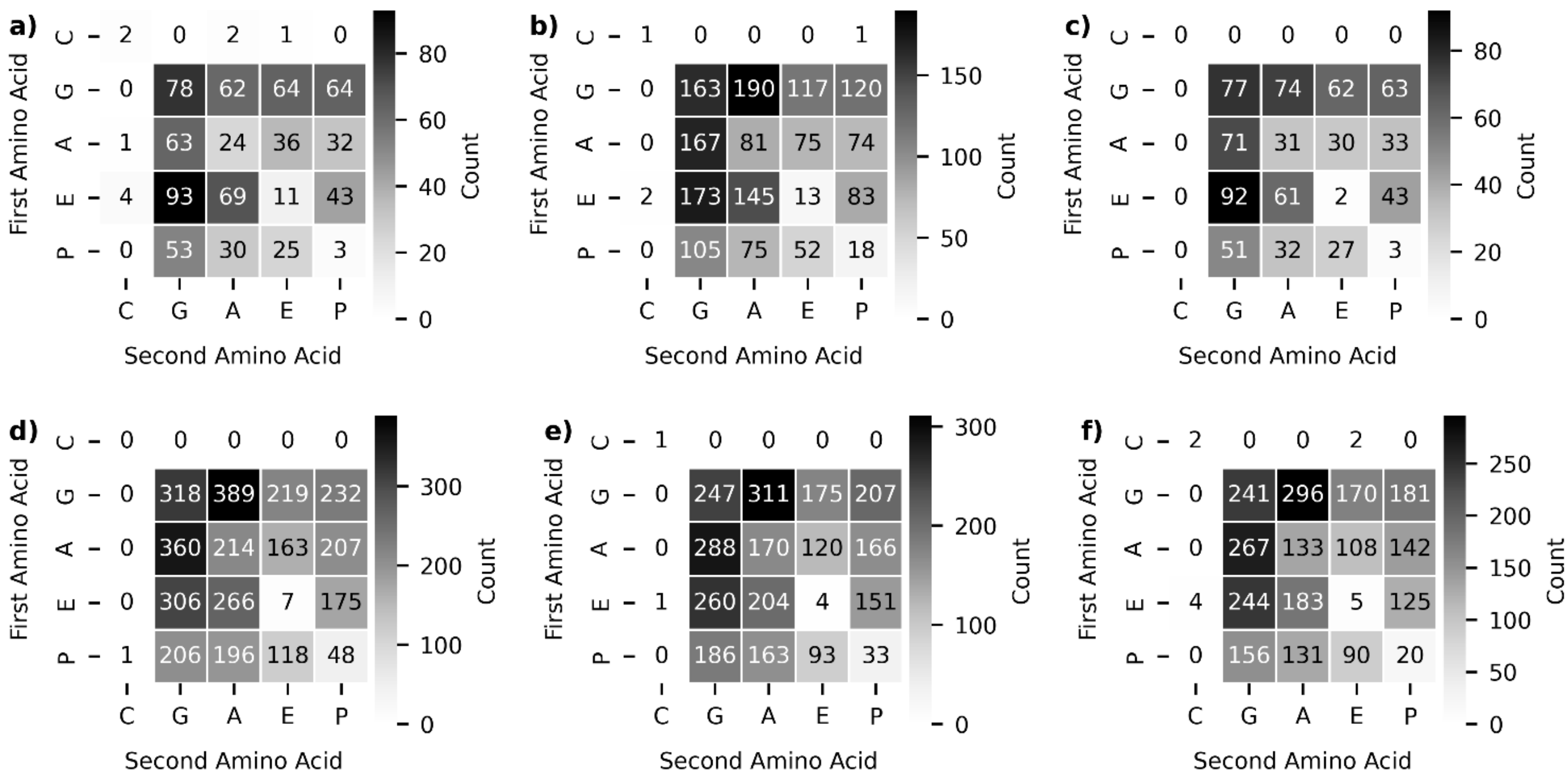

Figure S16. Bigram matrix of 2 amino acid sequence occurrence in observed peptides generated by wet-dry cycling of equimolar amounts of C, G, A, E, P at 130 °C for 4h and resuspending again throughout cycles from a) to f).

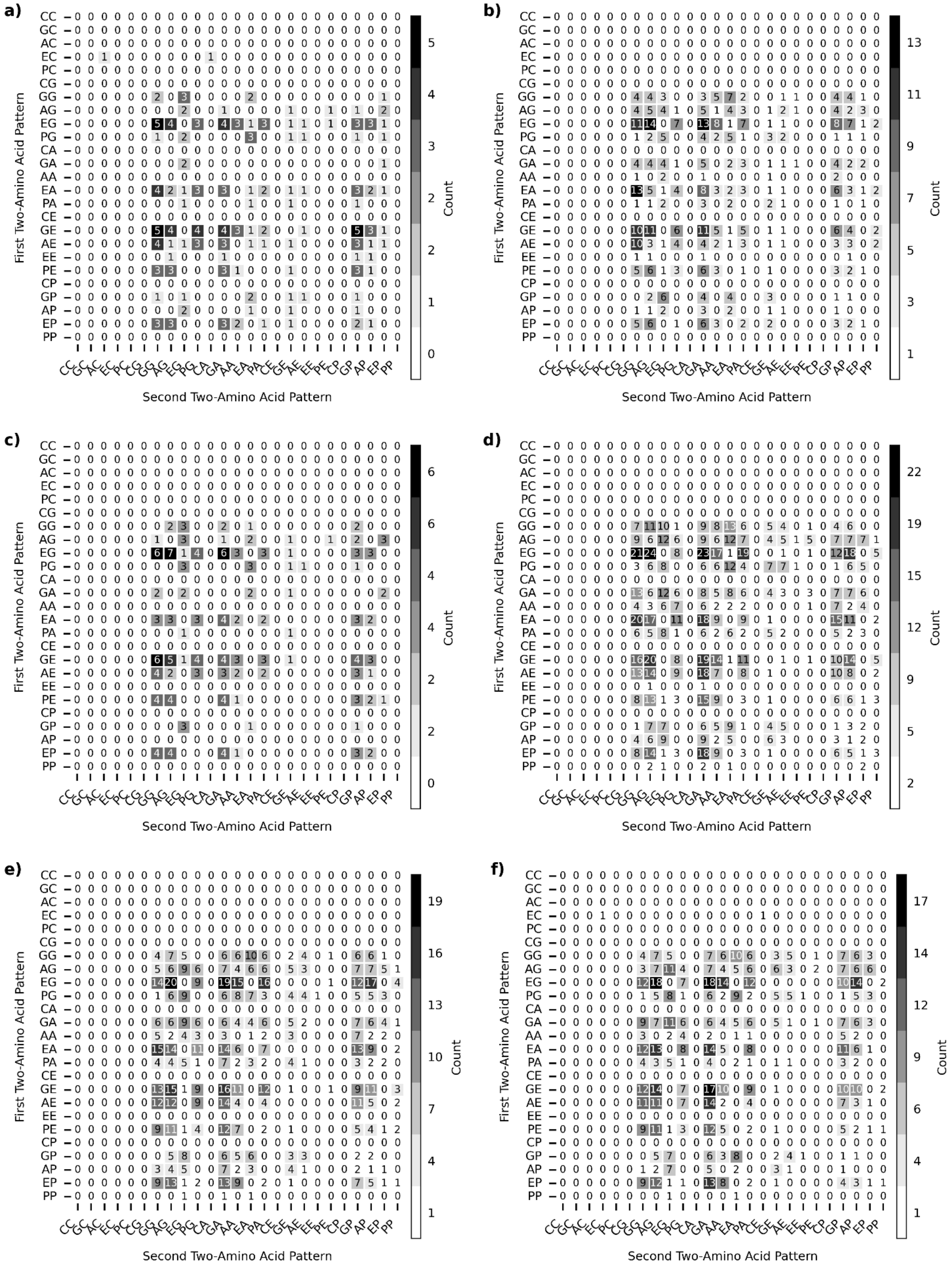

Figure S17 Bigram matrix of 4 amino acid sequence occurrence in observed peptides generated by wet-dry cycling of equimolar amounts of C, G, A, E, P at 130 °C for 4h and resuspending again throughout cycles from a) to f).

3.1.3 Amino acids F, G, A, E, P

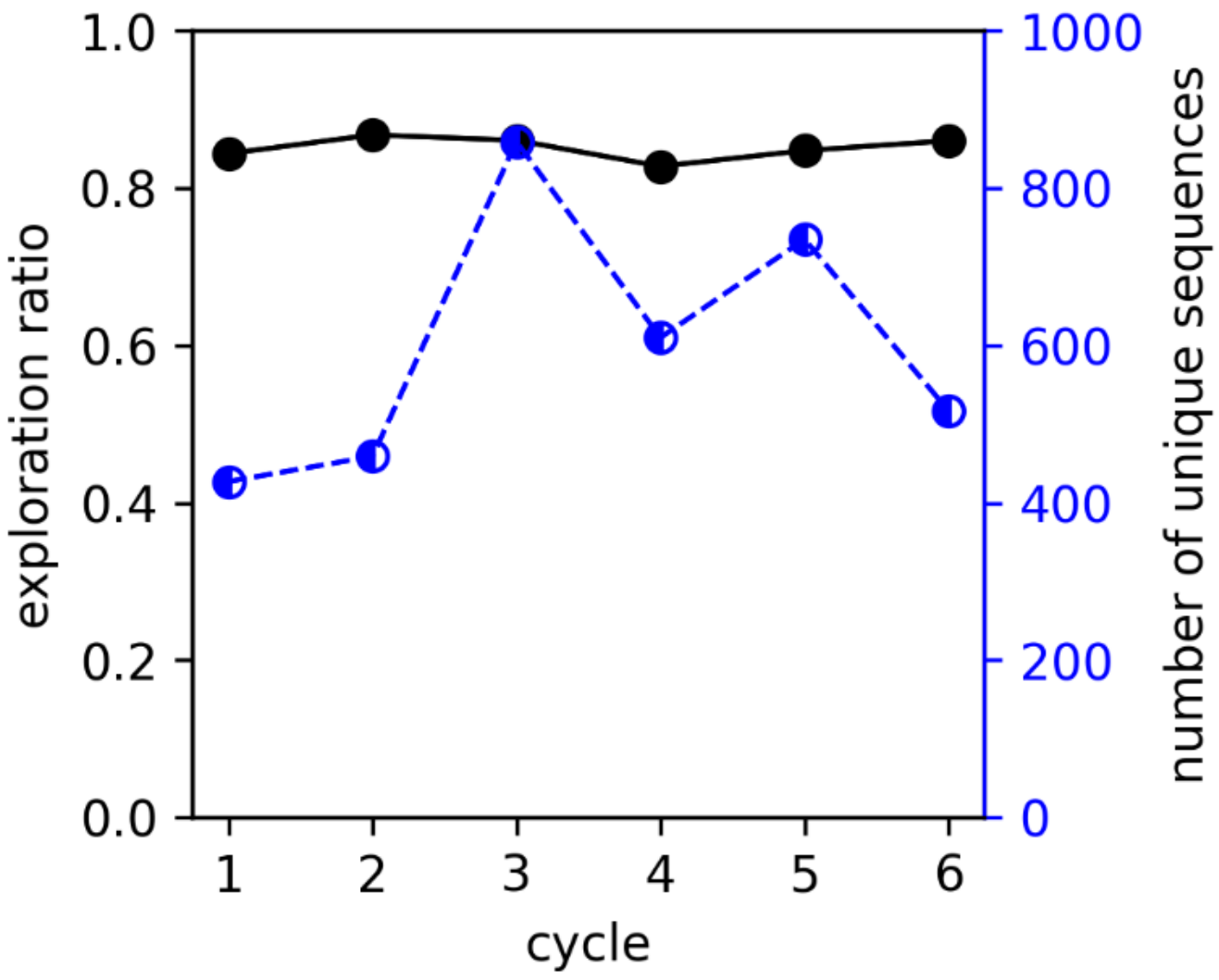

Figure S18. Number of unique peptides (blue) and exploration ratio (black) of peptide ensembles generated by wet-dry cycling of equimolar amounts of F, A, V, H, P at 130 °C for 4h and resuspending again (one cycle).

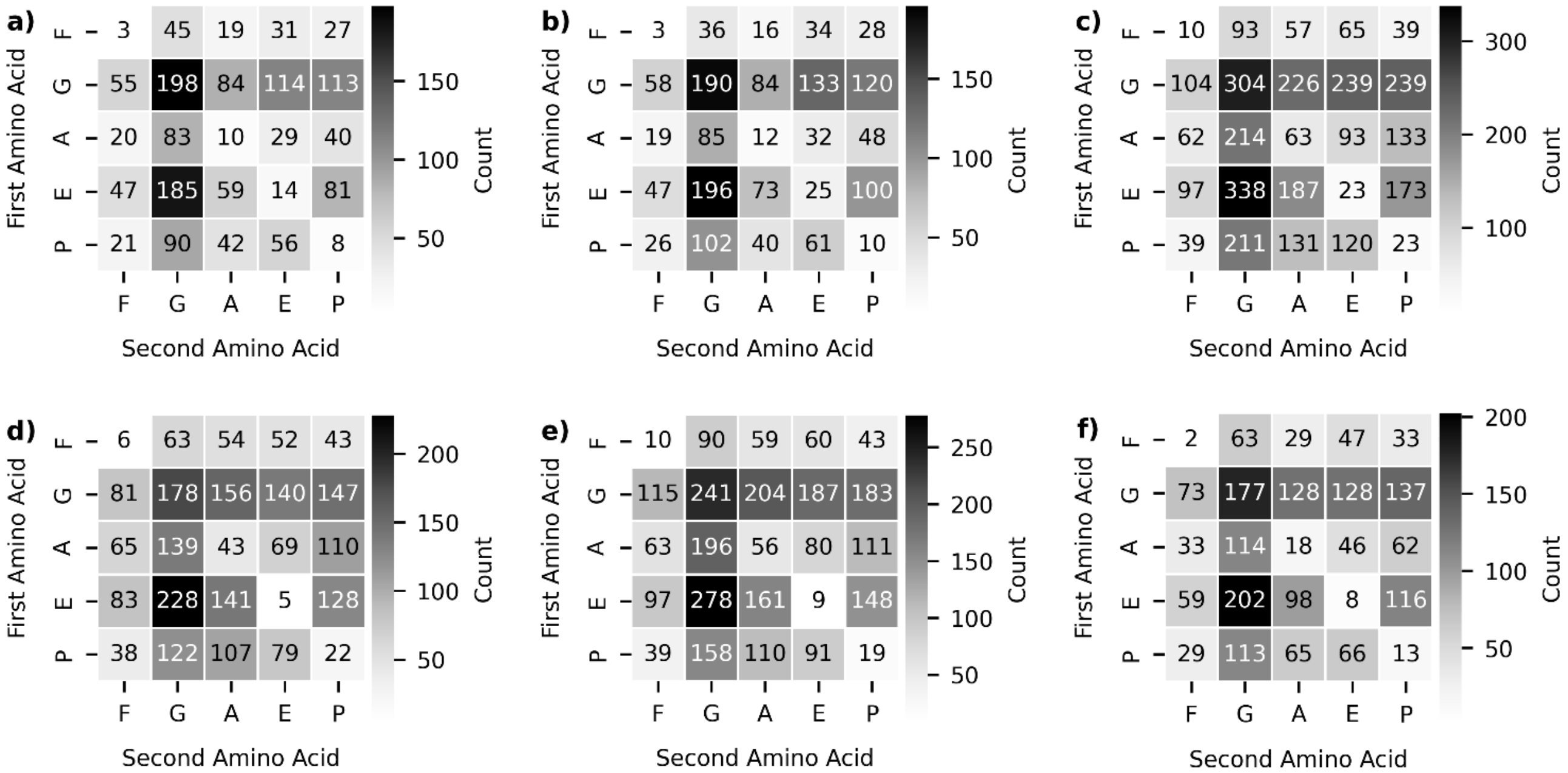

Figure S19. Bigram matrix of 2 amino acid sequence occurrence in observed peptides generated by wet-dry cycling of equimolar amounts of F, A, V, H, P at 130 °C for 4h and resuspending again throughout cycles from a) to f).

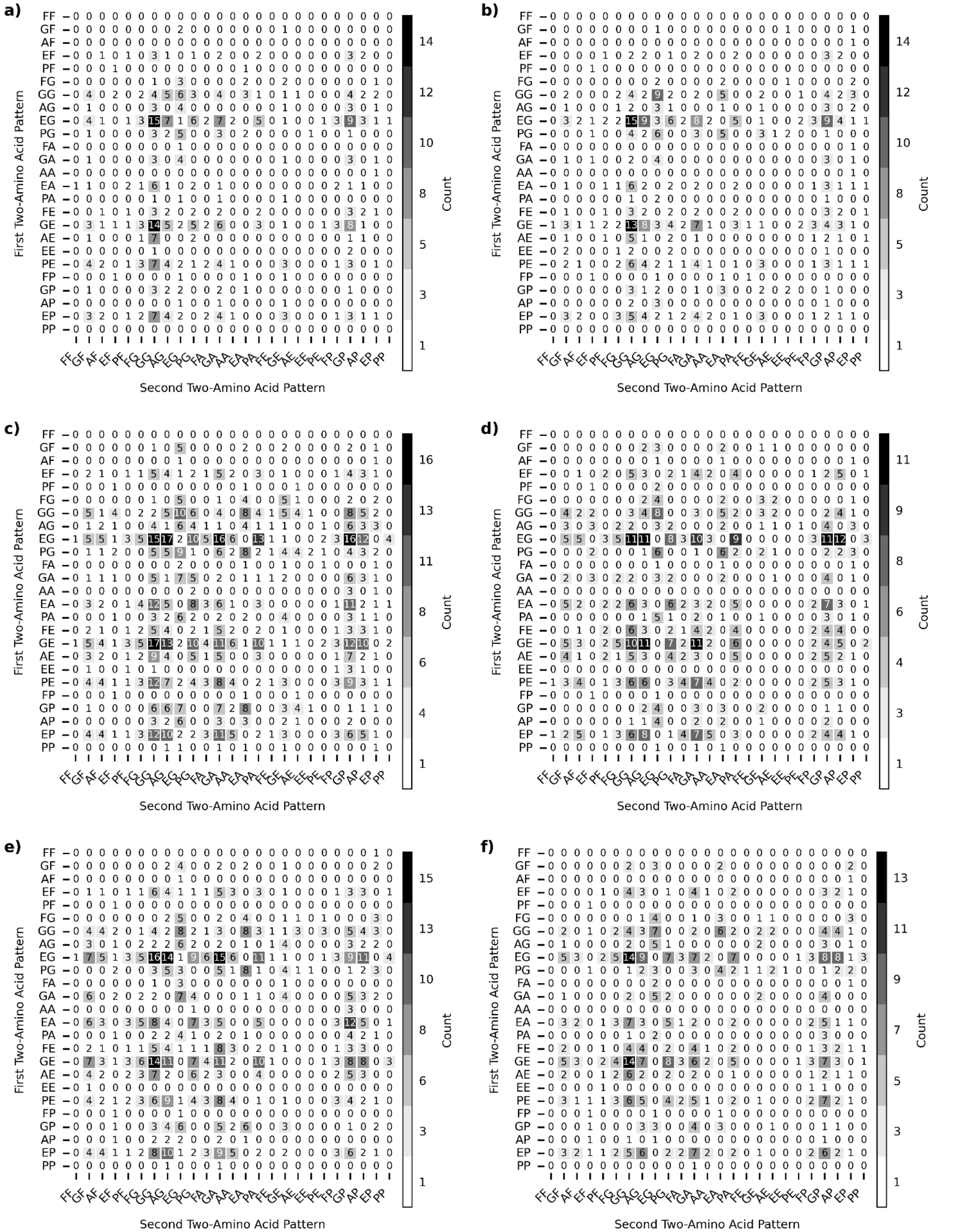

Figure S20 Bigram matrix of 4 amino acid sequence occurrence in observed peptides generated by wet-dry cycling of equimolar amounts of F, A, V, H, P at 130 °C for 4h and resuspending again throughout cycles from a) to f).

3.1.4 Amino acids F, H, G, A, E

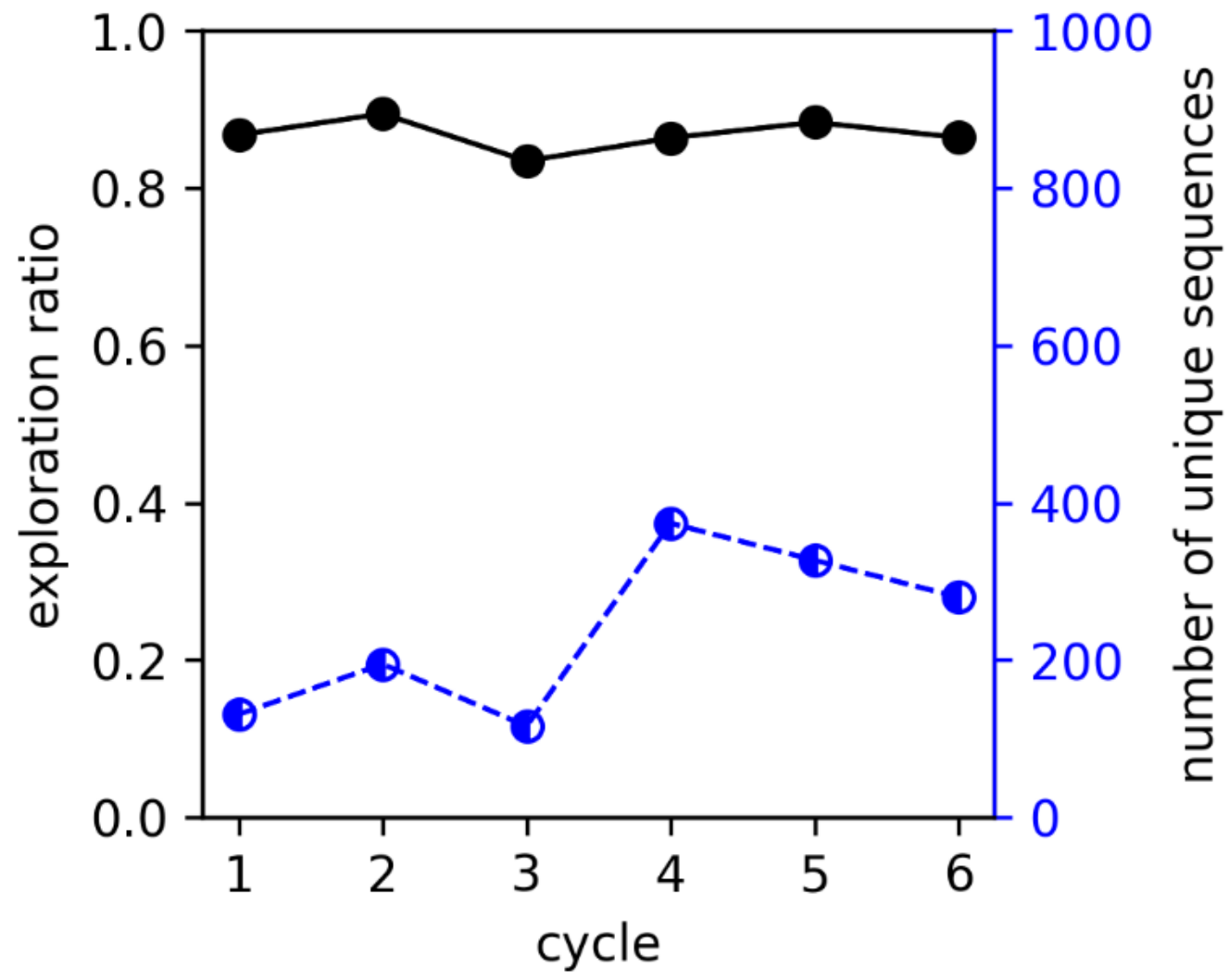

Figure S21. Number of unique peptides (blue) and exploration ratio (black) of peptide ensembles generated by wet-dry cycling of equimolar amounts of F, H, G, A, E at 130 °C for 4 h and resuspending again (one cycle).

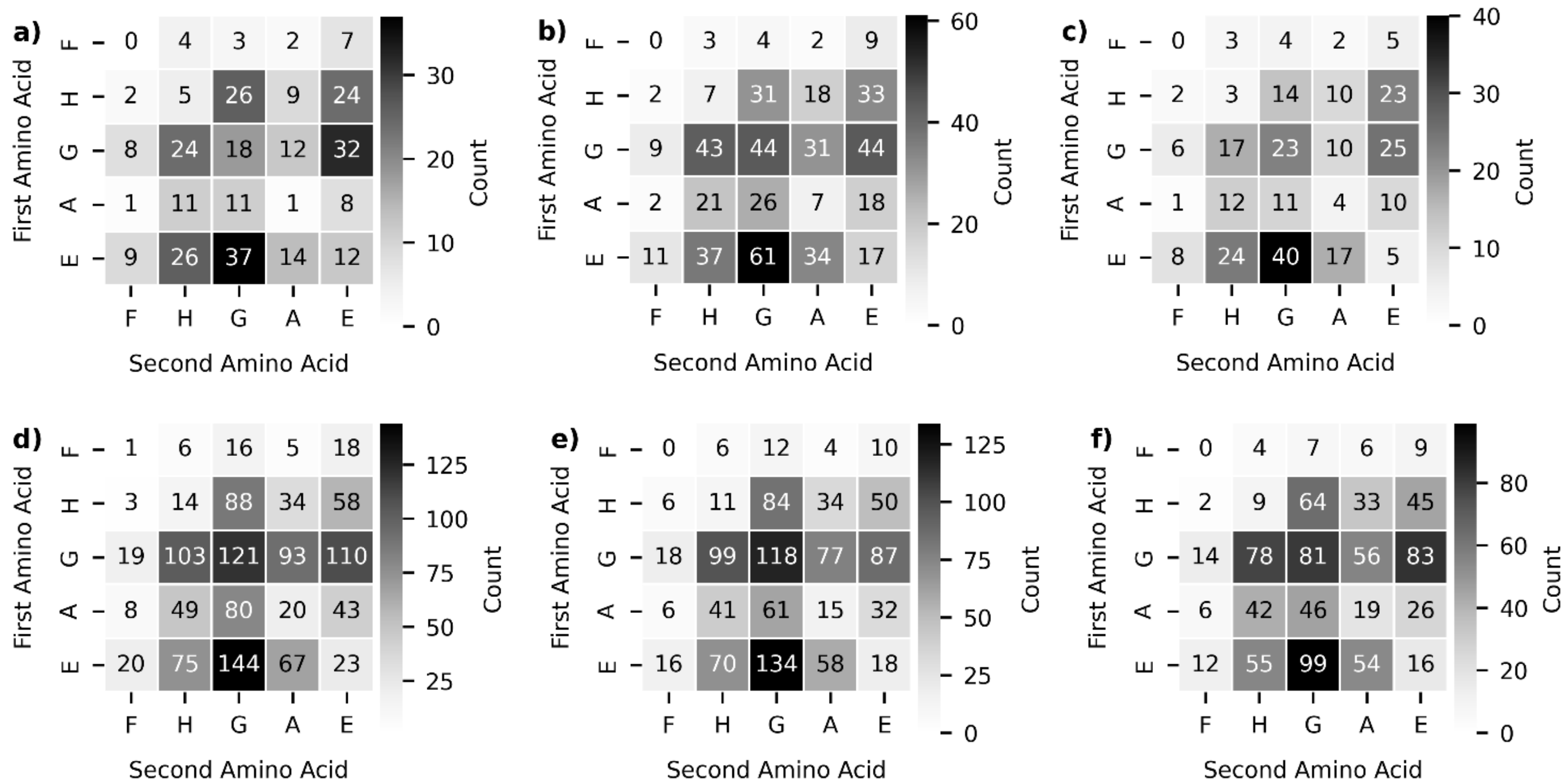

Figure S22. Bigram matrix of 2 amino acid sequence occurrence in observed peptides generated by wet-dry cycling of equimolar amounts of F, H, G, A, E at 130 °C for 4h and resuspending again throughout cycles from a) to f).

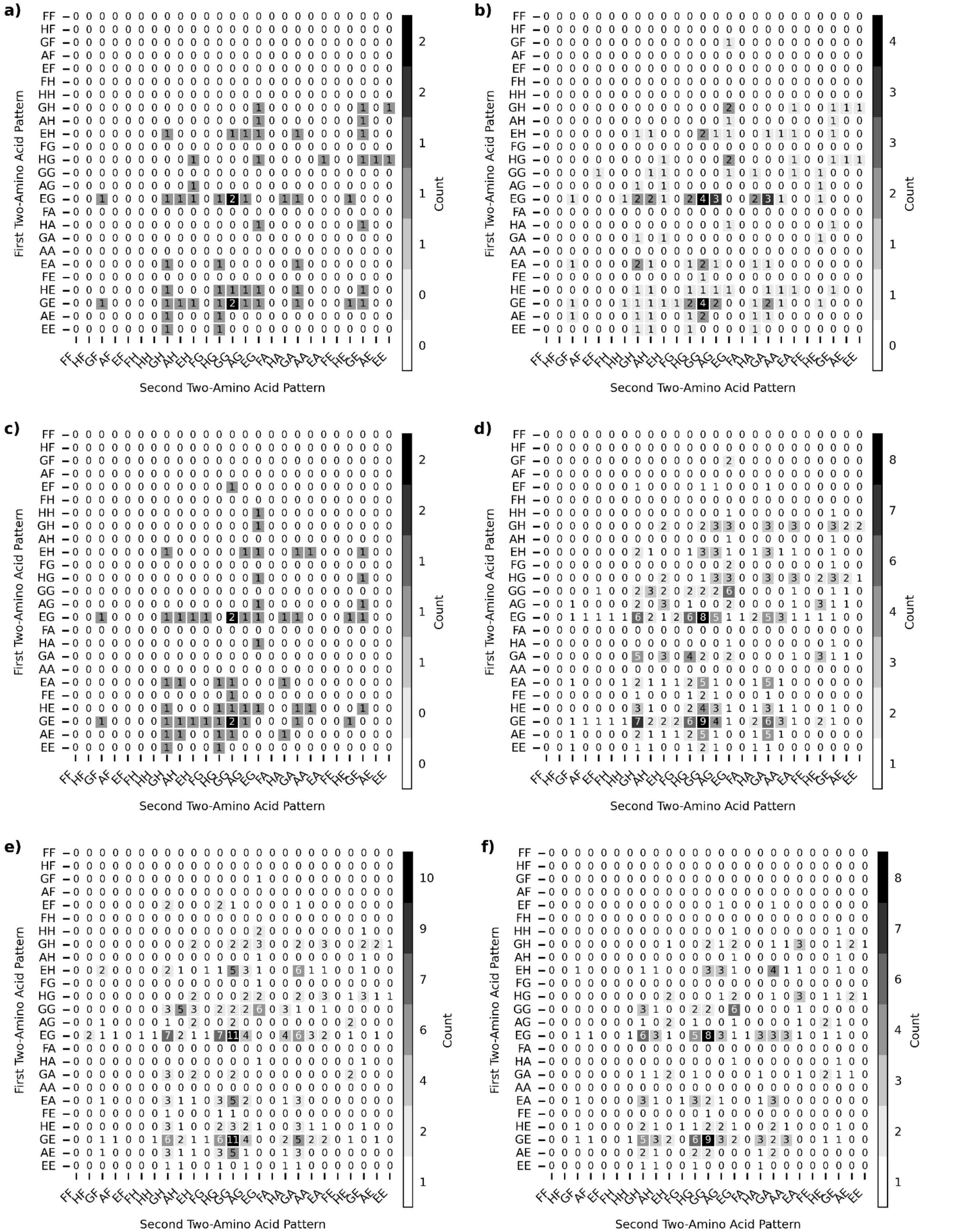

Figure S23 Bigram matrix of 4 amino acid sequence occurrence in observed peptides generated by wet-dry cycling of equimolar amounts of F, H, G, A, E at 130 °C for 4 h and resuspending again throughout cycles from a) to f).

3.1.5 Amino acids H, G, A, E, P

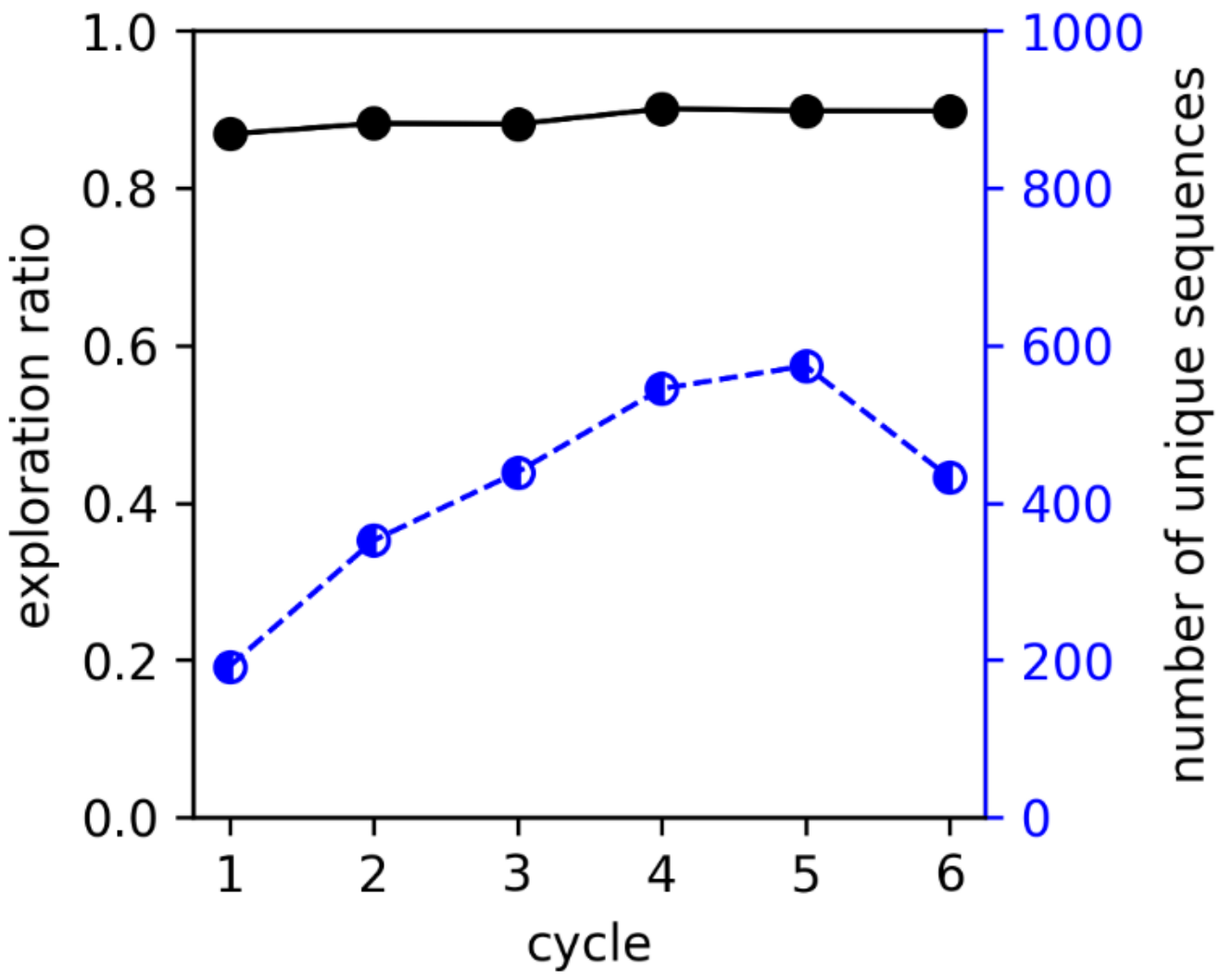

Figure S24. Number of unique peptides (blue) and exploration ratio (black) of peptide ensembles generated by wet-dry cycling of equimolar amounts of H, G, A, E, P at 130 °C for 4h and resuspending again (one cycle).

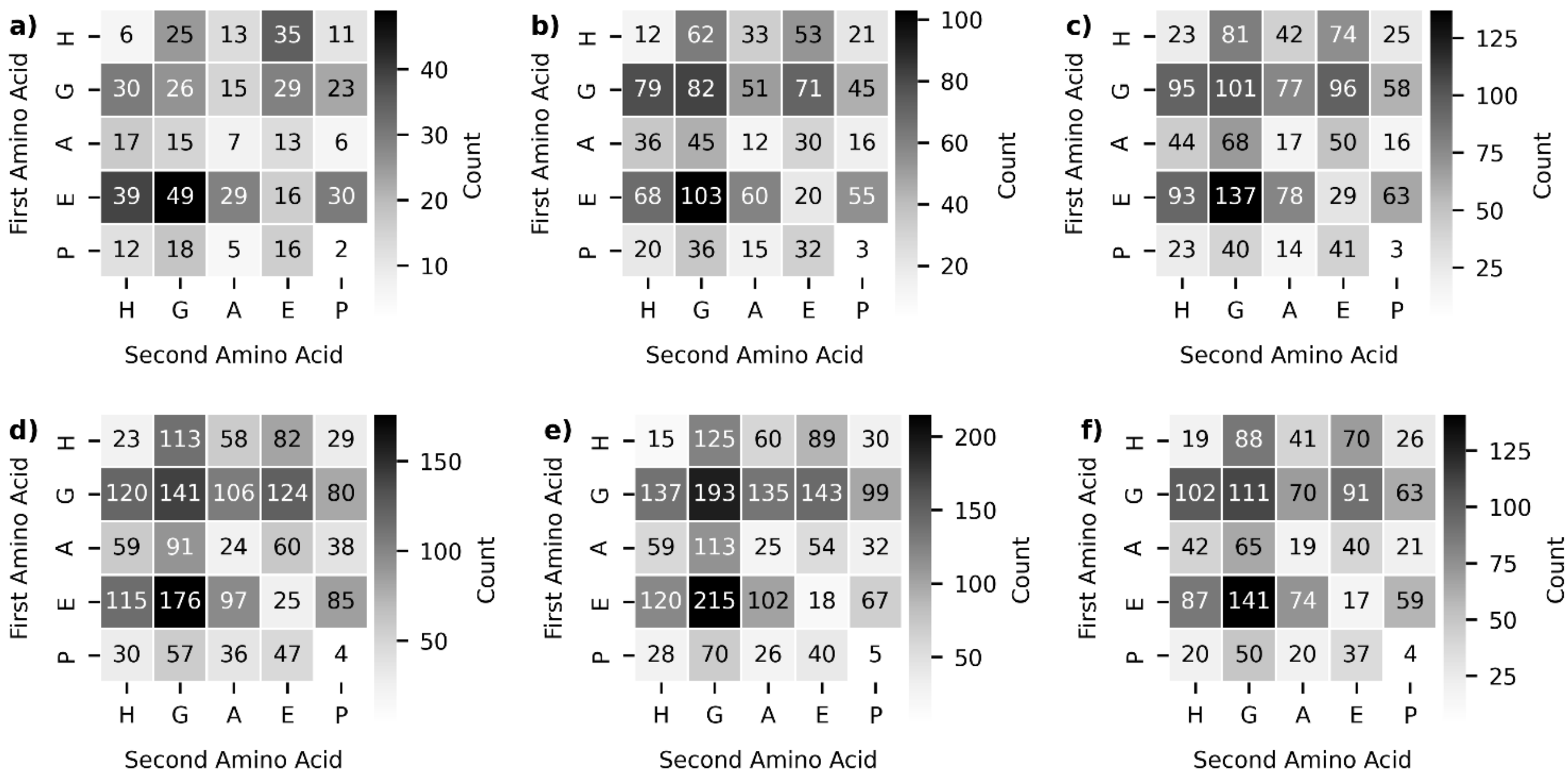

Figure S25. Bigram matrix of 2 amino acid sequence occurrence in observed peptides generated by wet-dry cycling of equimolar amounts of H, G, A, E, P at 130 °C for 4 h and resuspending again throughout cycles from a) to f).

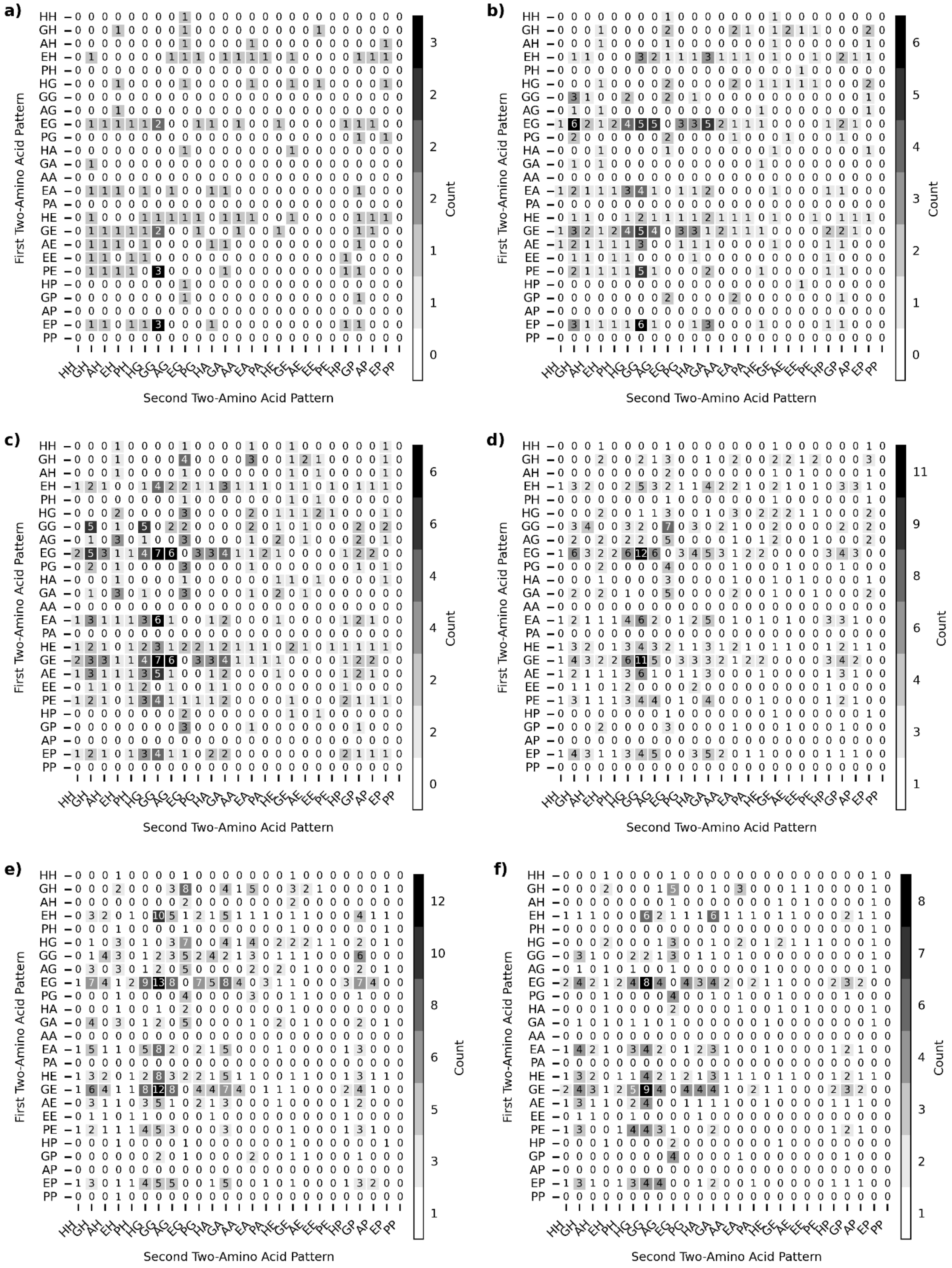

Figure S26 Bigram matrix of 4 amino acid sequence occurrence in observed peptides generated by wet-dry cycling of equimolar amounts of H, G, A, E, P at 130 °C for 4 h and resuspending again throughout cycles from a) to f).

3.1.6 Amino acids W, G, A, E, P

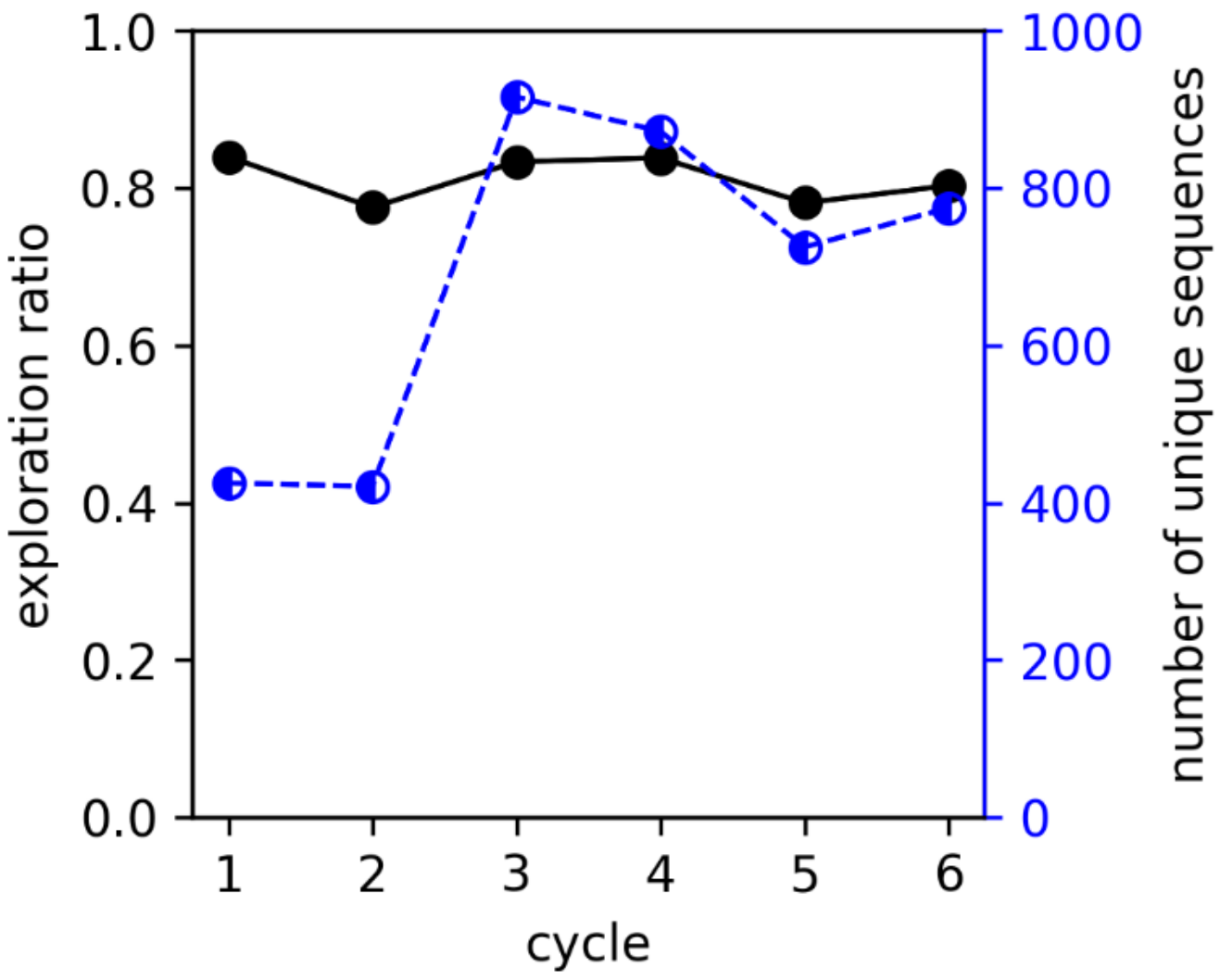

Figure S27. Number of unique peptides (blue) and exploration ratio (black) of peptide ensembles generated by wet-dry cycling of equimolar amounts of W, G, A, E, P at 130 °C for 4h and resuspending again (one cycle).

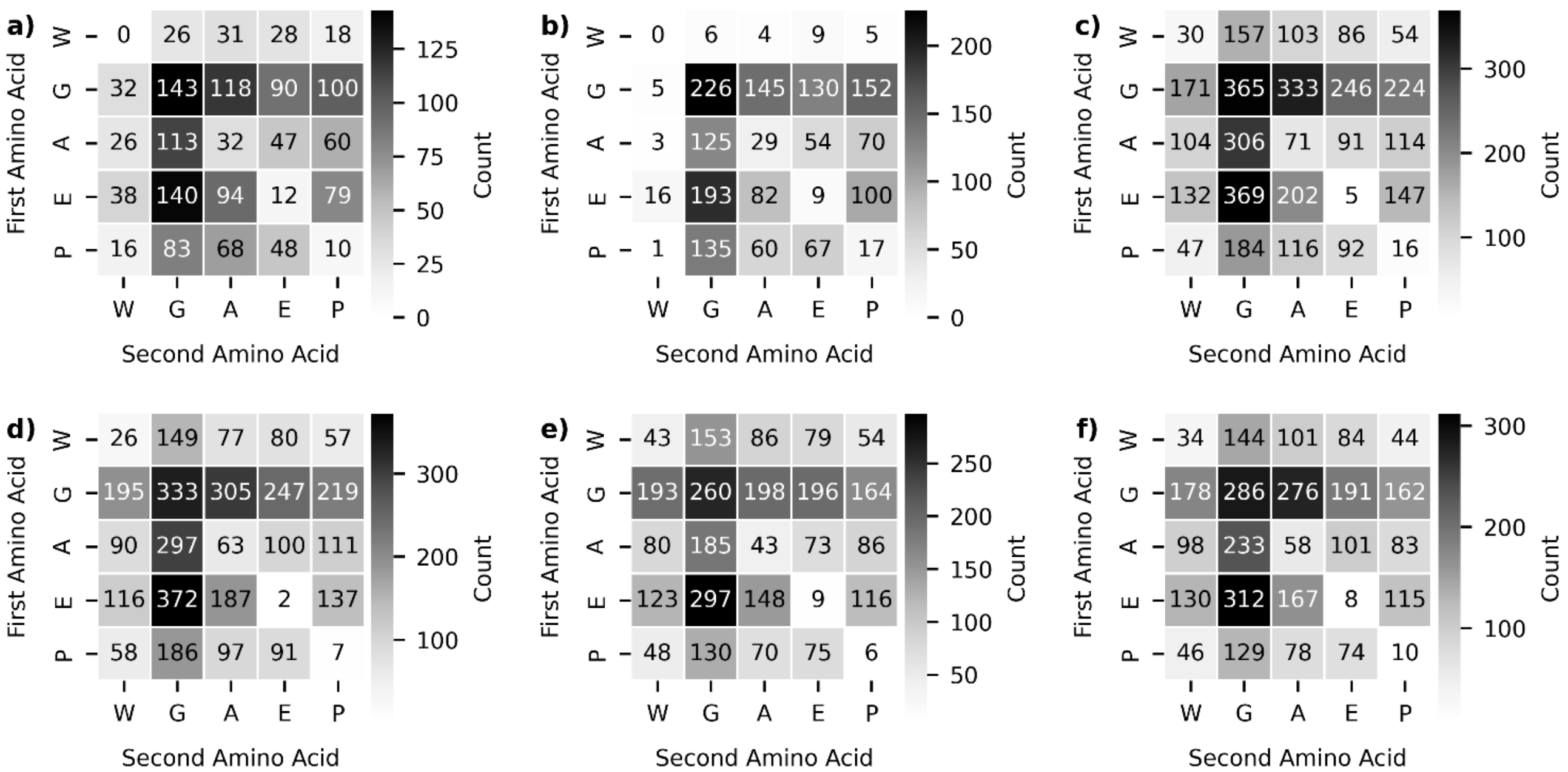

Figure S28. Bigram matrix of 2 amino acid sequence occurrence in observed peptides generated by wet-dry cycling of equimolar amounts of W, G, A, E, P at 130 °C for 4 h and resuspending again throughout cycles from a) to f).

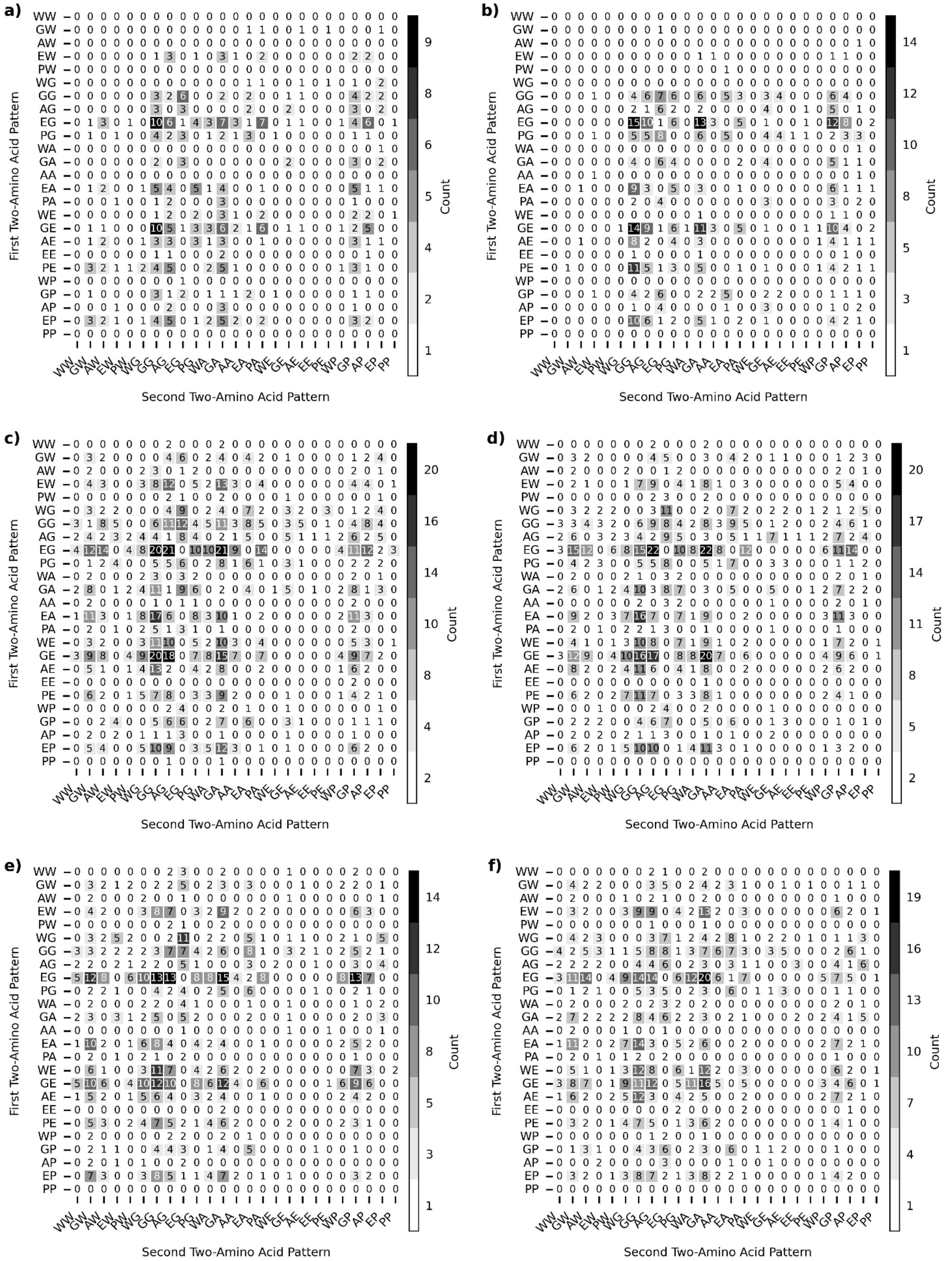

Figure S29 Bigram matrix of 4 amino acid sequence occurrence in observed peptides generated by wet-dry cycling of equimolar amounts of W, G, A, E, P at 130 °C for 4 h and resuspending again throughout cycles from a) to f).

3.1.7 Amino acids G, D, H, S

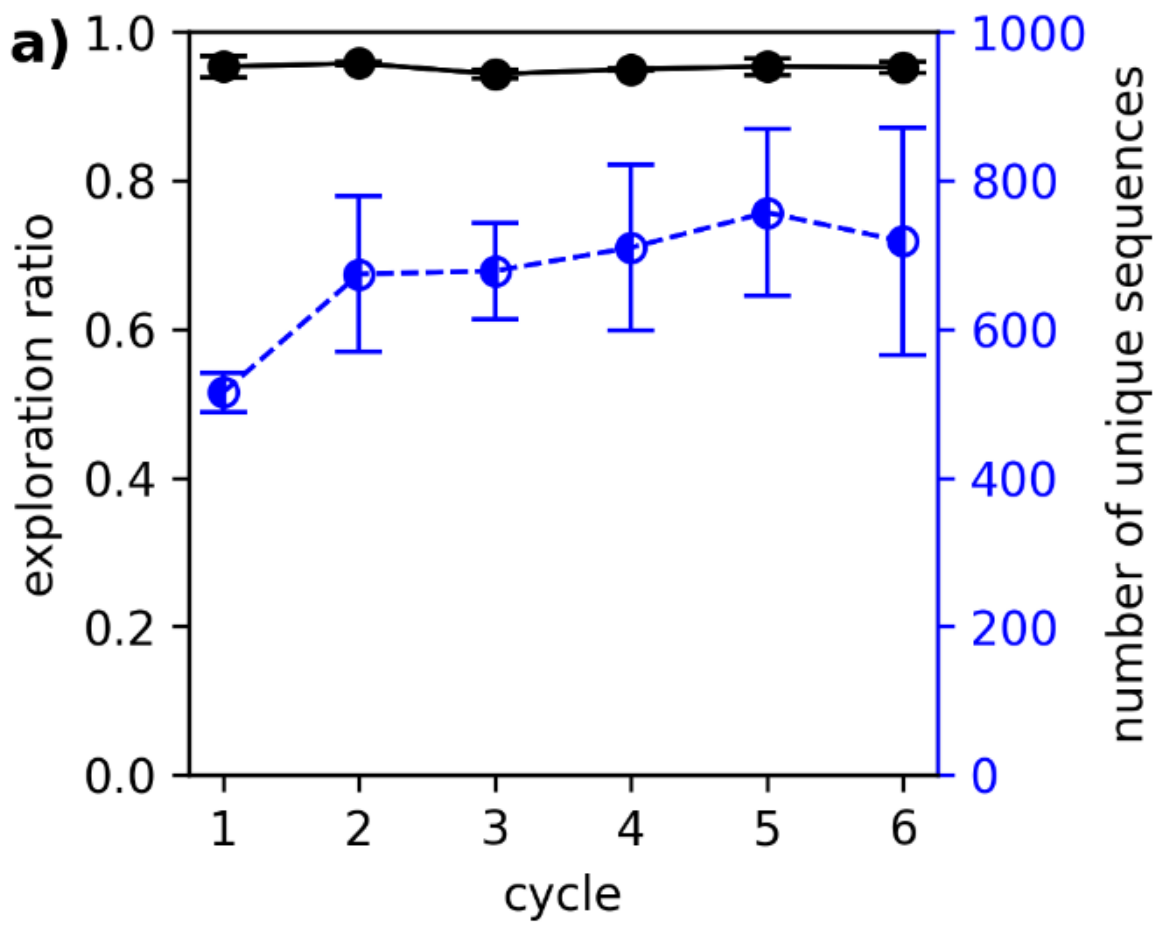

Figure S30. Number of unique peptides (blue) and exploration ratio (black) of peptide ensembles generated by wet-dry cycling of equimolar amounts of G, D, H, S at 130 °C for 4h and resuspending again (one cycle). Average values for replicates a) and single trajectory b) used for bigram matrix generations (see bellow).

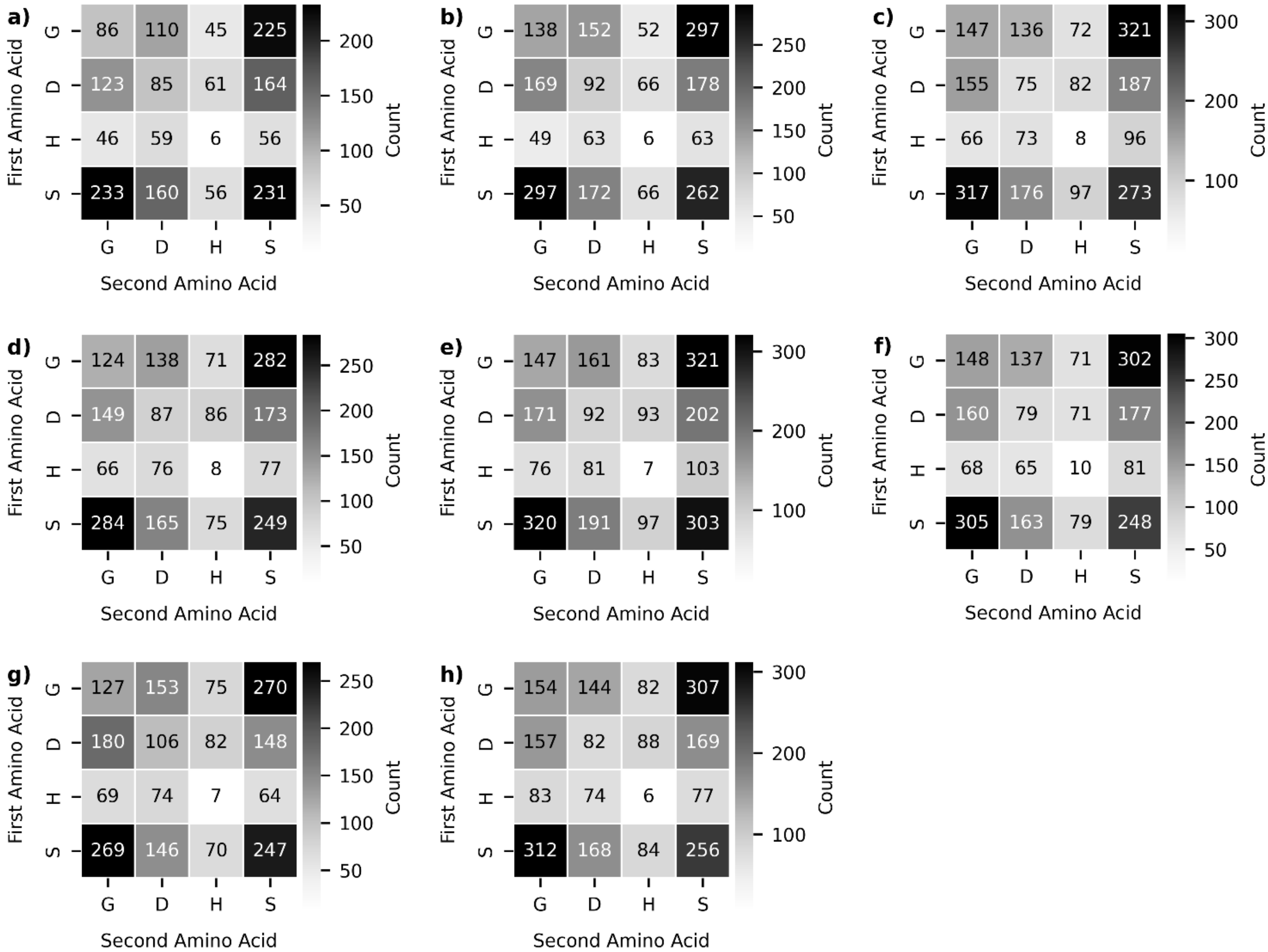

Figure S31. Bigram matrix of 2 amino acid sequence occurrence in observed peptides generated by wet-dry cycling of equimolar amounts of G, D, H, S at 130 °C for 4 h and resuspending again throughout cycles from a) to h).

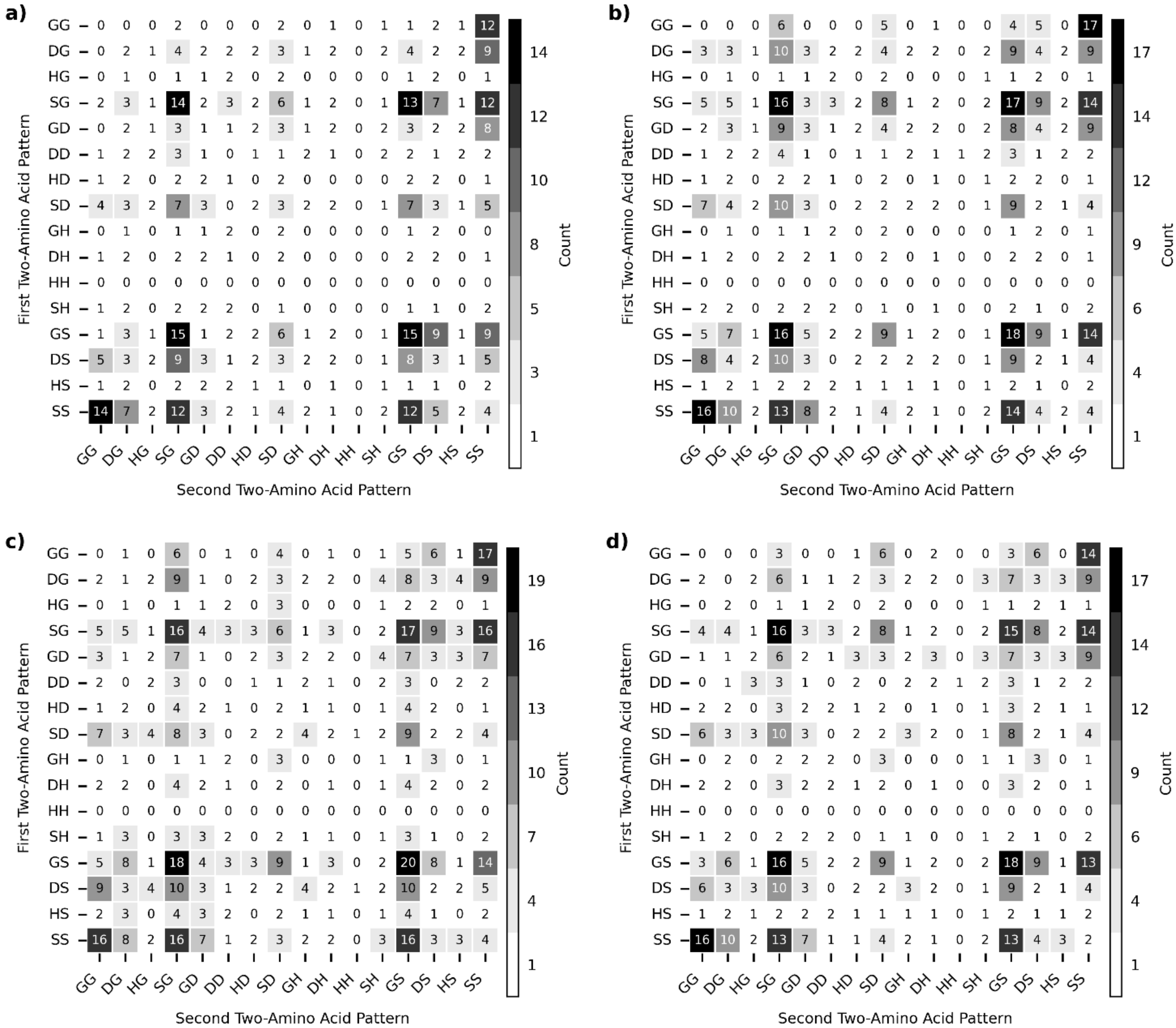

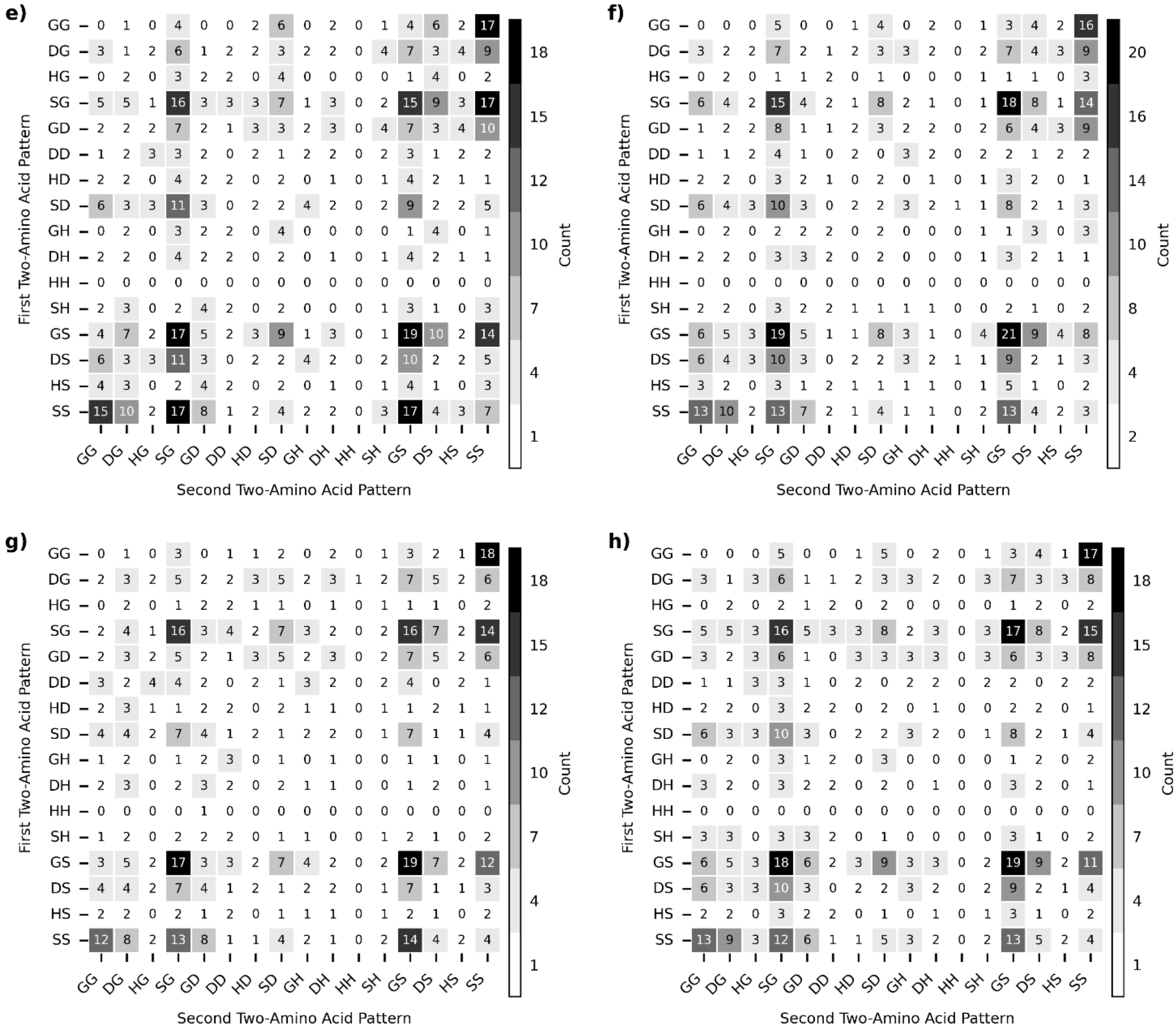

Figure S32 Bigram matrix of 4 amino acid sequence occurrence in observed peptides generated by wet-dry cycling of equimolar amounts of G, D, H, S at 130 °C for 4 h and resuspending again throughout cycles from a) to h).

3.2 CDI in solution

An example of undirected type of open-ended exploration: Addition of CDI solution into aqueous solutions of G, A, V, H, P at equal amounts of amino acids, clearly showing no bias in product distribution. There was a clear steady increase in the number of unique objects. The exploration ratio stayed very close to 1, regardless of the number of unique species.

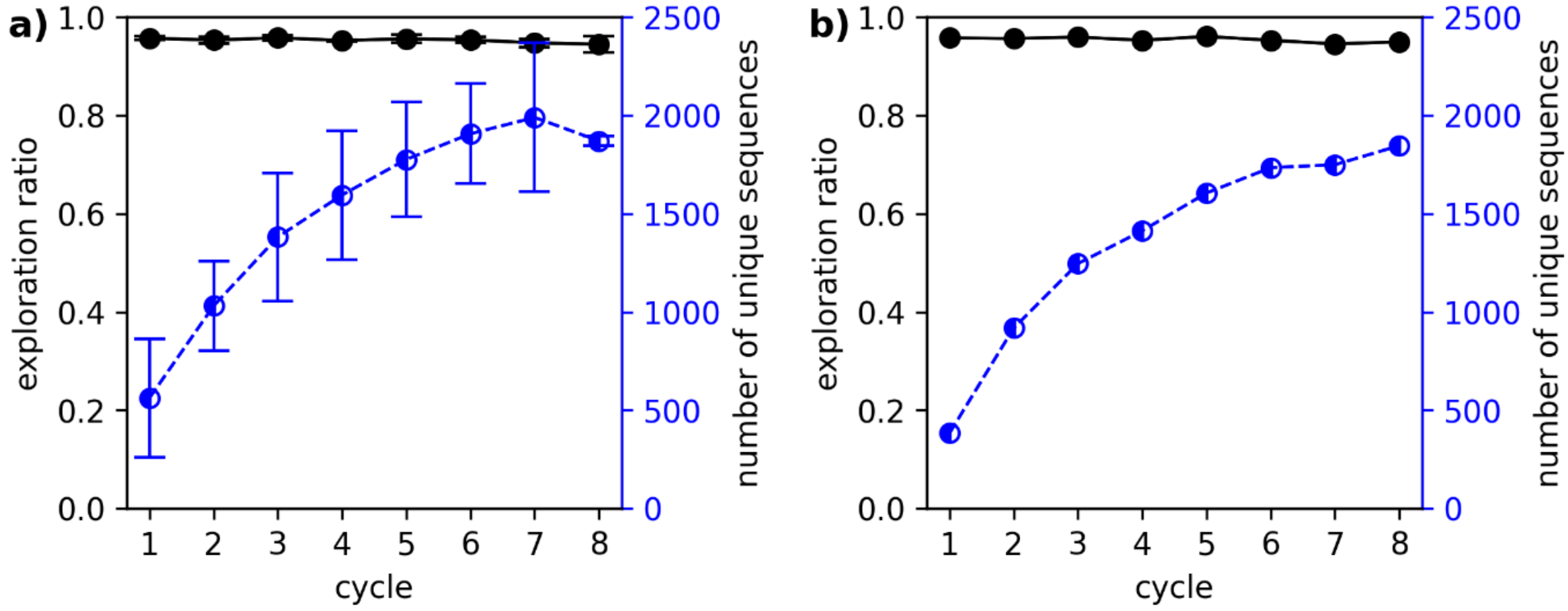

Figure S33. Number of unique peptides (blue) and exploration ratio (black) of peptide ensembles generated by addition of CDI solution to aqueous solutions of amino acids throughout cycles. Average values for replicates a) and single trajectory b) used for bigram matrix generations (see bellow).

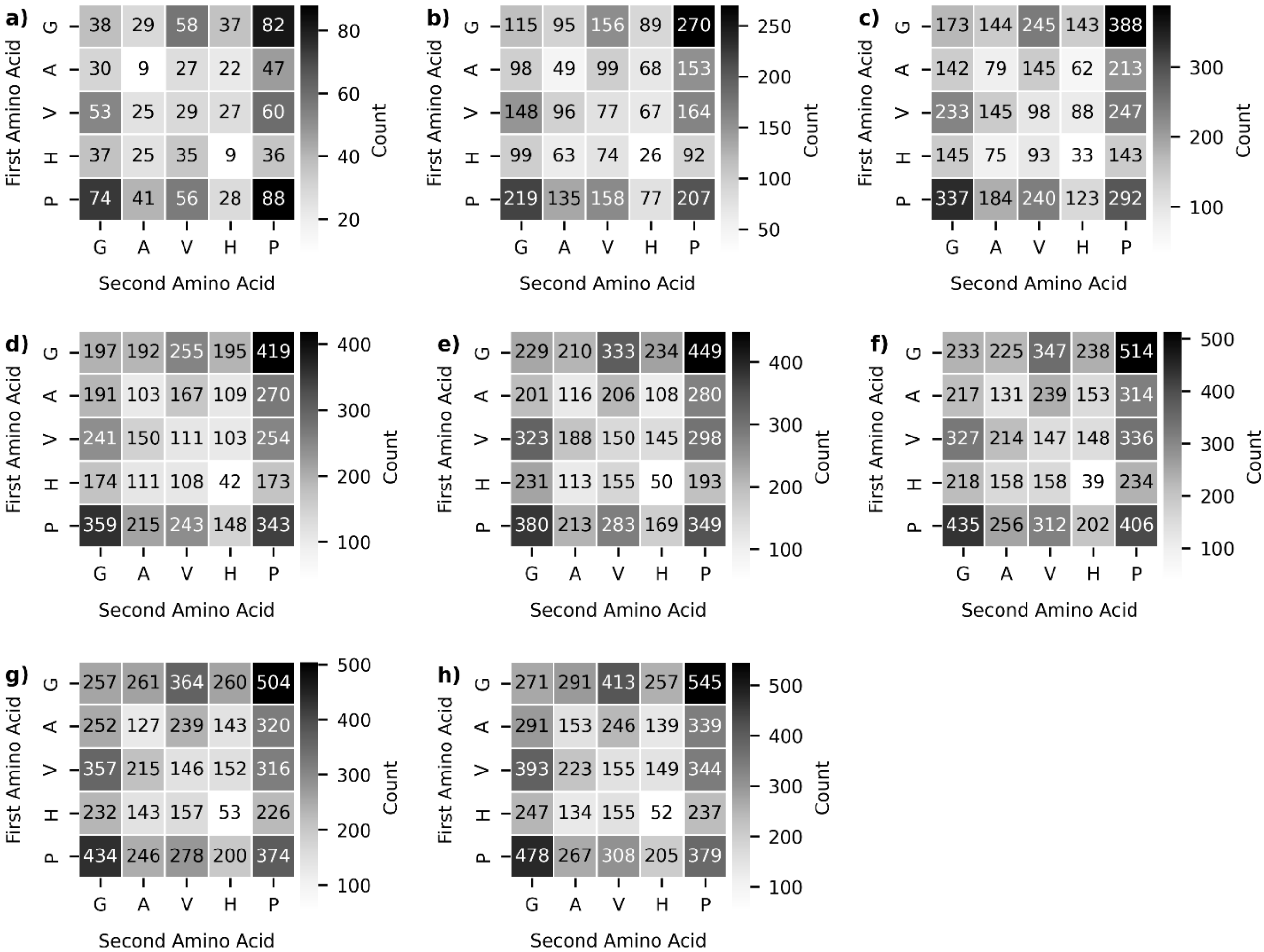

Figure S34. Bigram matrix of 2 amino acid sequence occurrence in observed peptides generated by addition of CDI solution to aqueous solutions of amino acids throughout cycles from a) to h).

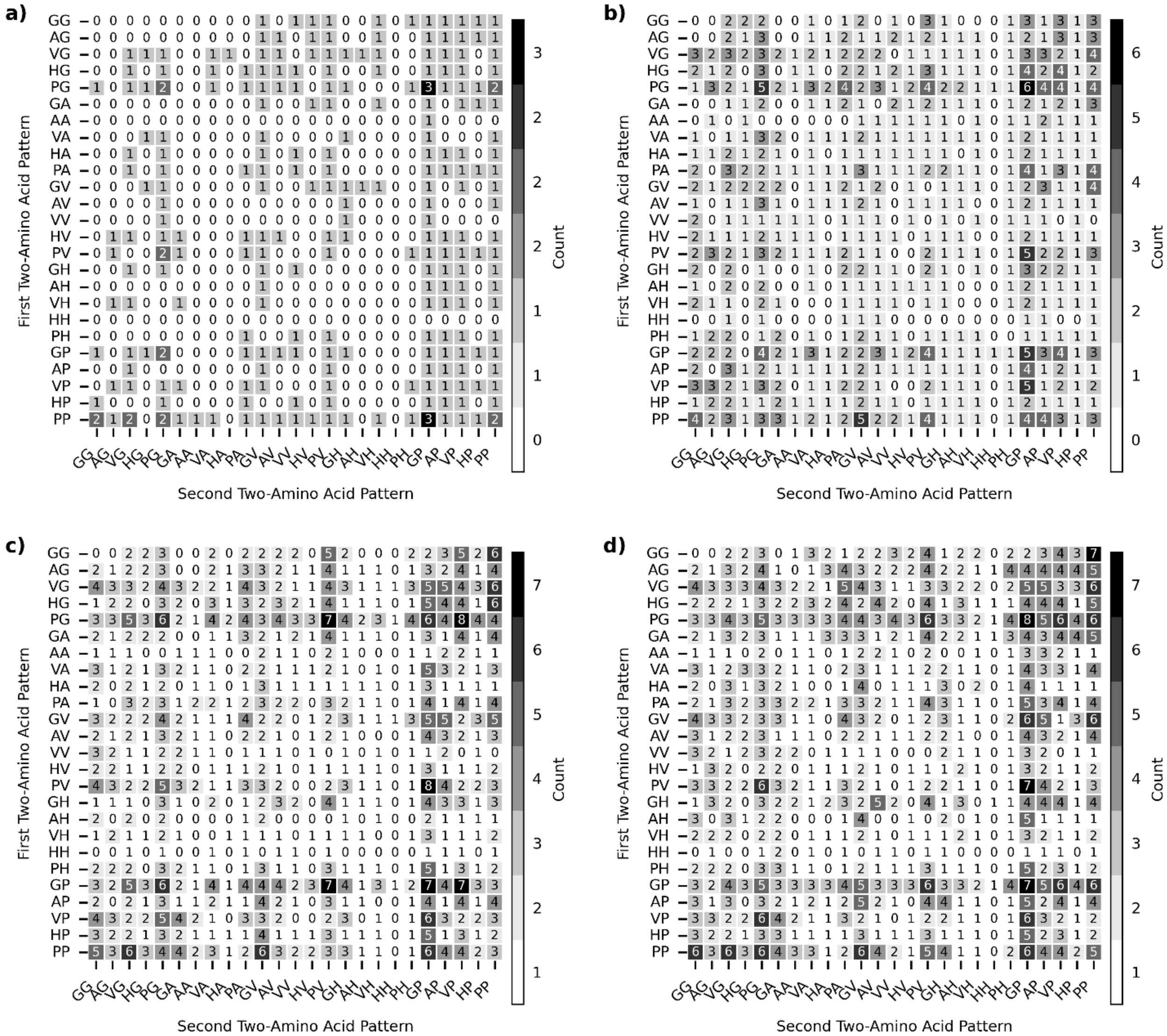
a)
b)
c)
d)
First Two-Amino Acid Pattern
Second Two-Amino Acid Pattern
Count
GG AG VG HG PG GA AA VA HA PA GV AV VV HV PV GH AH VH HH PH GP AP VP HP PP

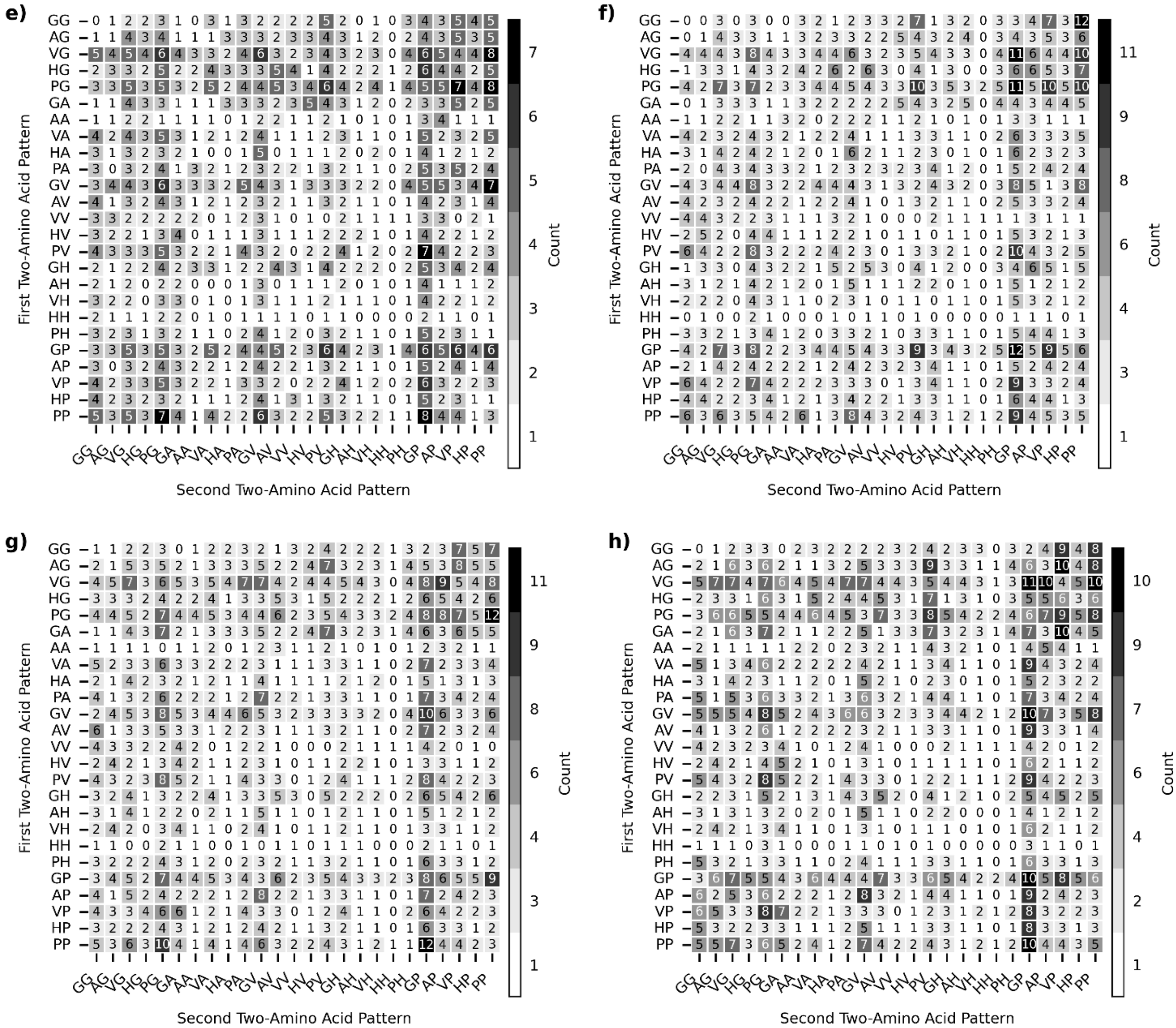

Figure S35. Bigram matrix of 4 amino acid sequence occurrence in observed peptides generated by addition of CDI solution to aqueous solutions of amino acids throughout cycles from a) to h).

3.3 CDI in solid state

To investigate whether the undirected exploration observed in liquid-phase CDI reactions is preserved under mechanochemical conditions, we examined peptide formation using solid amino acids and solid CDI in a ball mill system. This approach eliminates solvation effects and creates a heterogeneous reaction environment where peptide bond formation occurs through direct solid-solid contact under mechanical energy input. The solid-state methodology provides a complementary perspective to solution chemistry by removing differential solubility effects that might influence amino acid availability and accessibility. The solid-state experiments thus serve as an important control for validating the generality of assembly theory metrics across different reaction media and physical constraints.

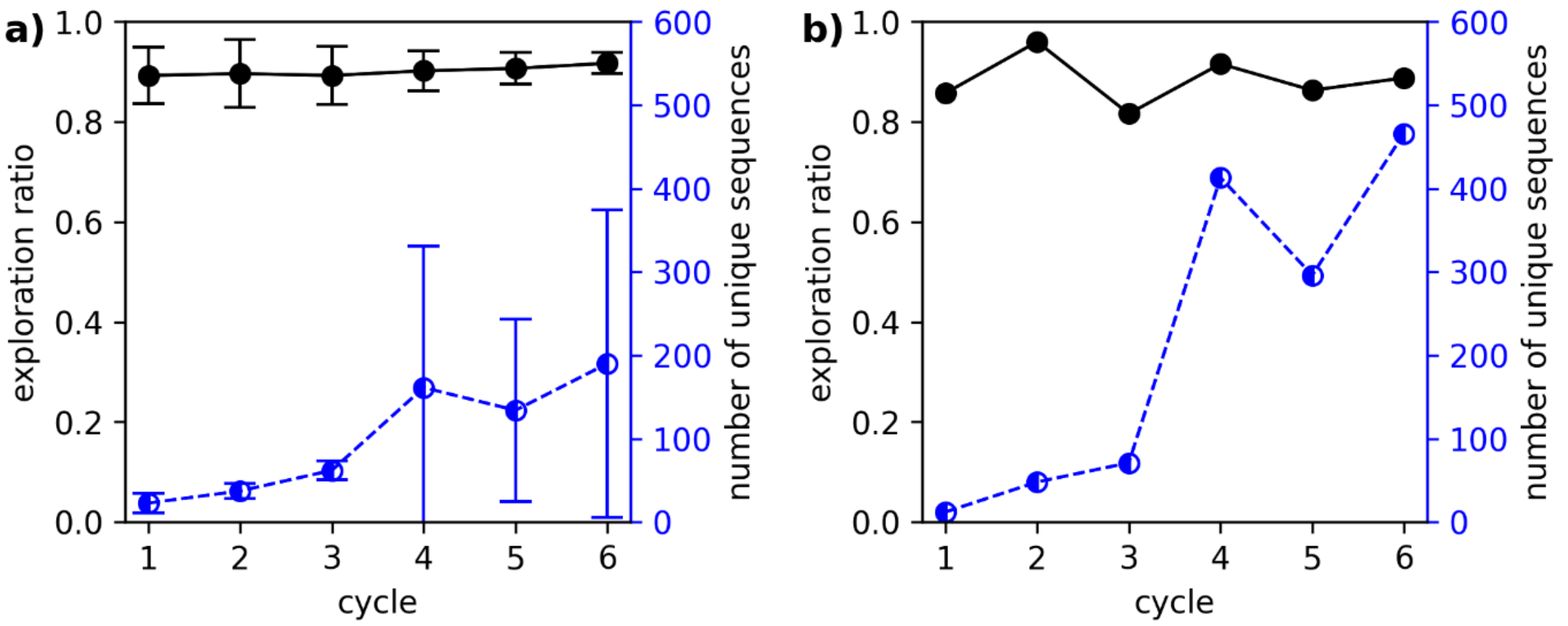

Figure S36. Number of unique peptides (blue) and exploration ratio (black) of peptide ensembles generated by agitating solid amino acids (equimolar amounts of G, A, V, H, P) with solid CDI in ball mill. Average values for replicates a) and single trajectory b) used for bigram matrix generations (see bellow).

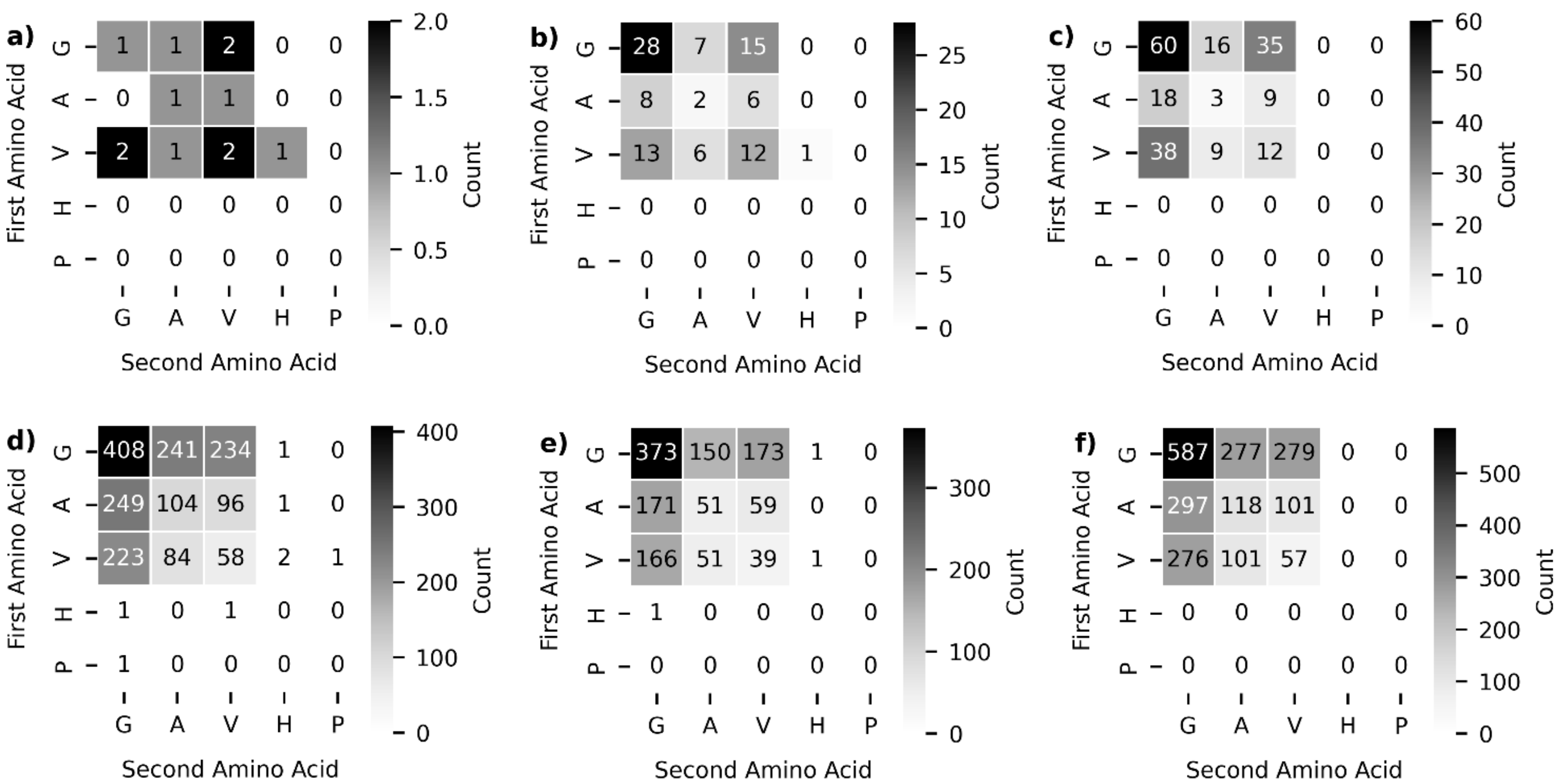

Figure S37. Bigram matrix of 2 amino acid sequence occurrence in observed peptides generated by agitating solid amino acids (equimolar amounts of G, A, V, H, P) with solid CDI in ball mill throughout cycles from a) to h).

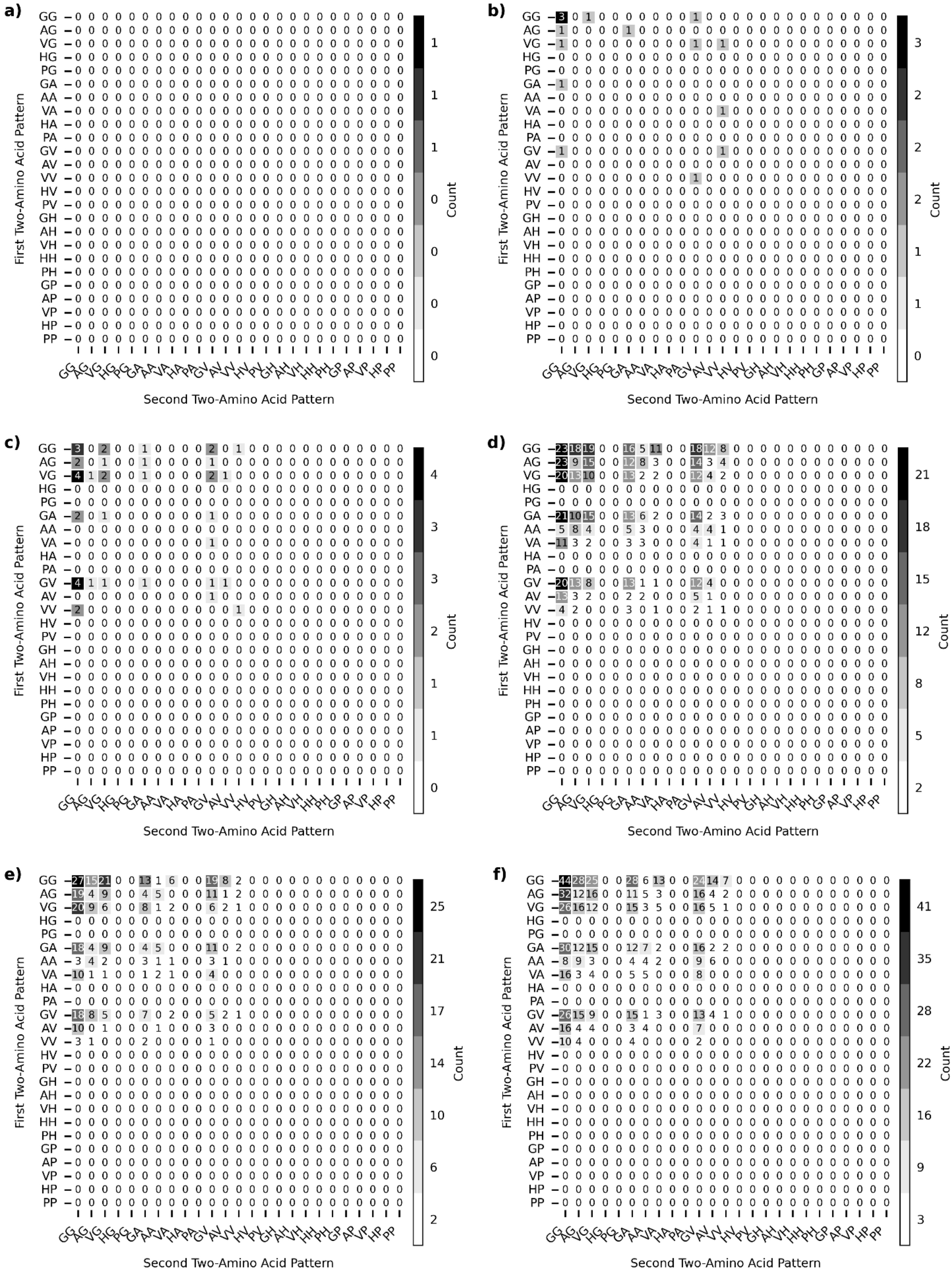

Figure S38. Bigram matrix of 4 amino acid sequence occurrence in observed peptides generated by agitating solid amino acids (equimolar amounts of G, A, V, H, P) with solid CDI in ball mill throughout cycles from a) to h).

3.4 Catalysis with proteases

Protease-catalyzed peptide formation experiments were conducted using mechanochemical ball milling of amino acid methyl esters with enzymatic catalysts to investigate directed exploration in peptide sequence space. Two distinct protease classes were examined: cysteine proteases (papain and bromelain) requiring L-cysteine cofactor and Na_2CO_3 base, and serine proteases (trypsin and chymotrypsin) with their characteristic substrate specificities. Each enzyme system was tested through multiple milling cycles to monitor progressive peptide formation and sequence selectivity patterns. The protease experiments serve as positive controls for directed exploration, where known enzymatic substrate preferences should result in significantly lower exploration ratios compared to undirected coupling methods.

3.4.1 Cystein proteases

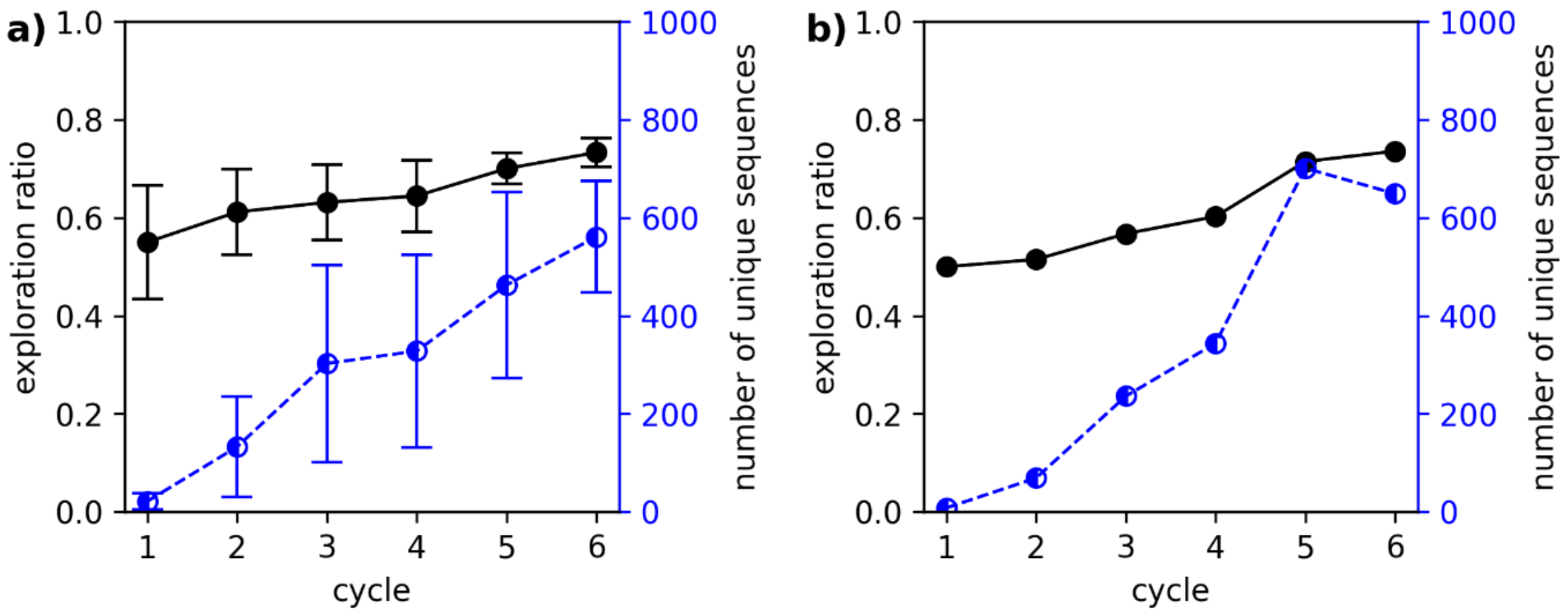

Figure S39. Number of unique peptides (blue) and exploration ratio (black) of peptide ensembles generated by agitating solid amino acid methyl esters (equimolar amounts of G, A, V, H, P) with cysteine proteases papain or bromelain in ball mill. Average values for replicates a) and single trajectory b) used for bigram matrix generations (see bellow).

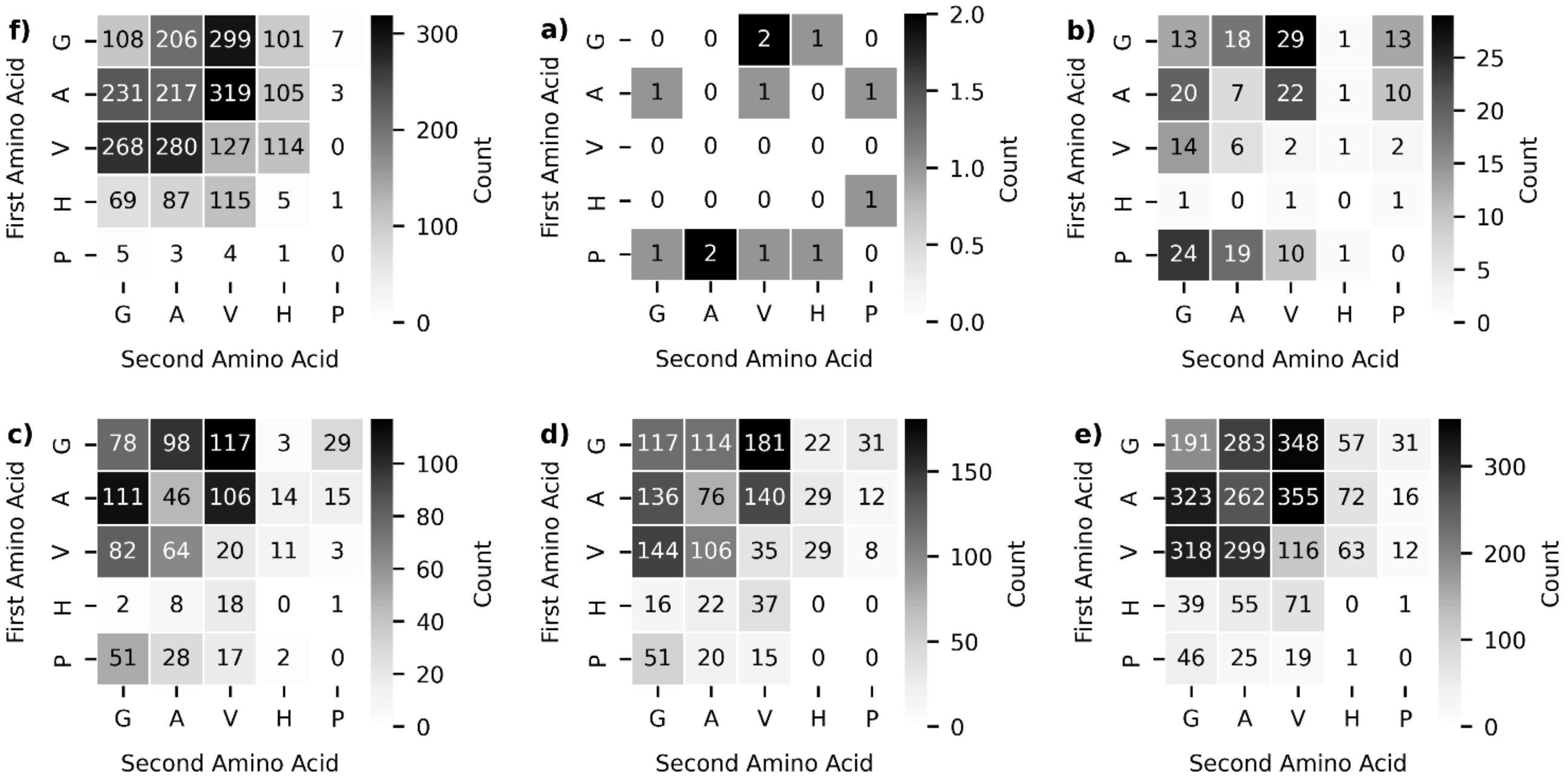

Figure S40. Bigram matrix of 2 amino acid sequence occurrence in observed peptides generated by agitating solid amino acid methyl esters (equimolar amounts of G, A, V, H, P) with cysteine proteases papain or bromelain in ball mill throughout cycles from a) to h).

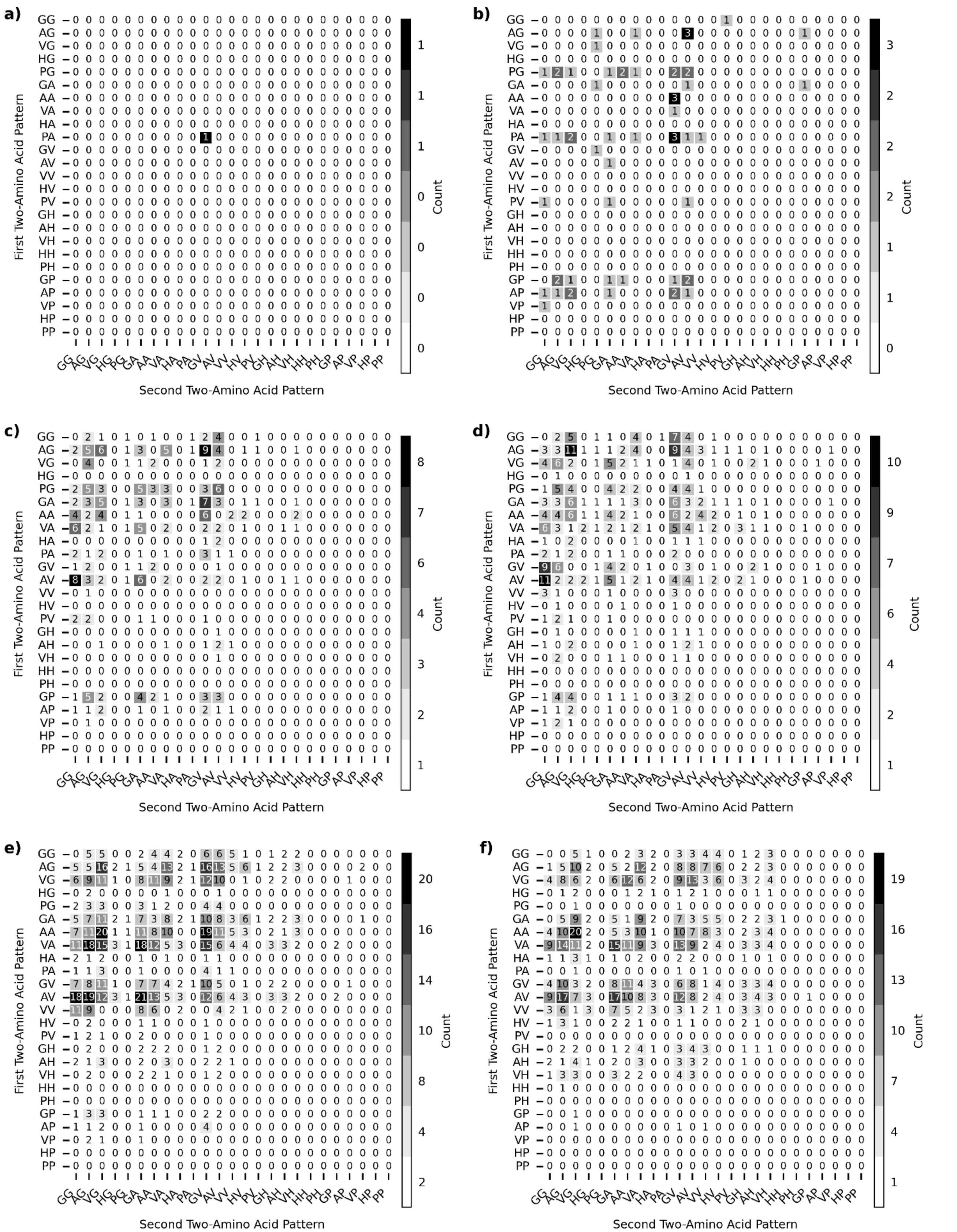

Figure S41. Bigram matrix of 4 amino acid sequence occurrence in observed peptides generated by agitating solid amino acid methyl esters (equimolar amounts of G, A, V, H, P) with cysteine proteases papain or bromelain in ball mill throughout cycles from a) to h).

3.4.2 Serine proteases

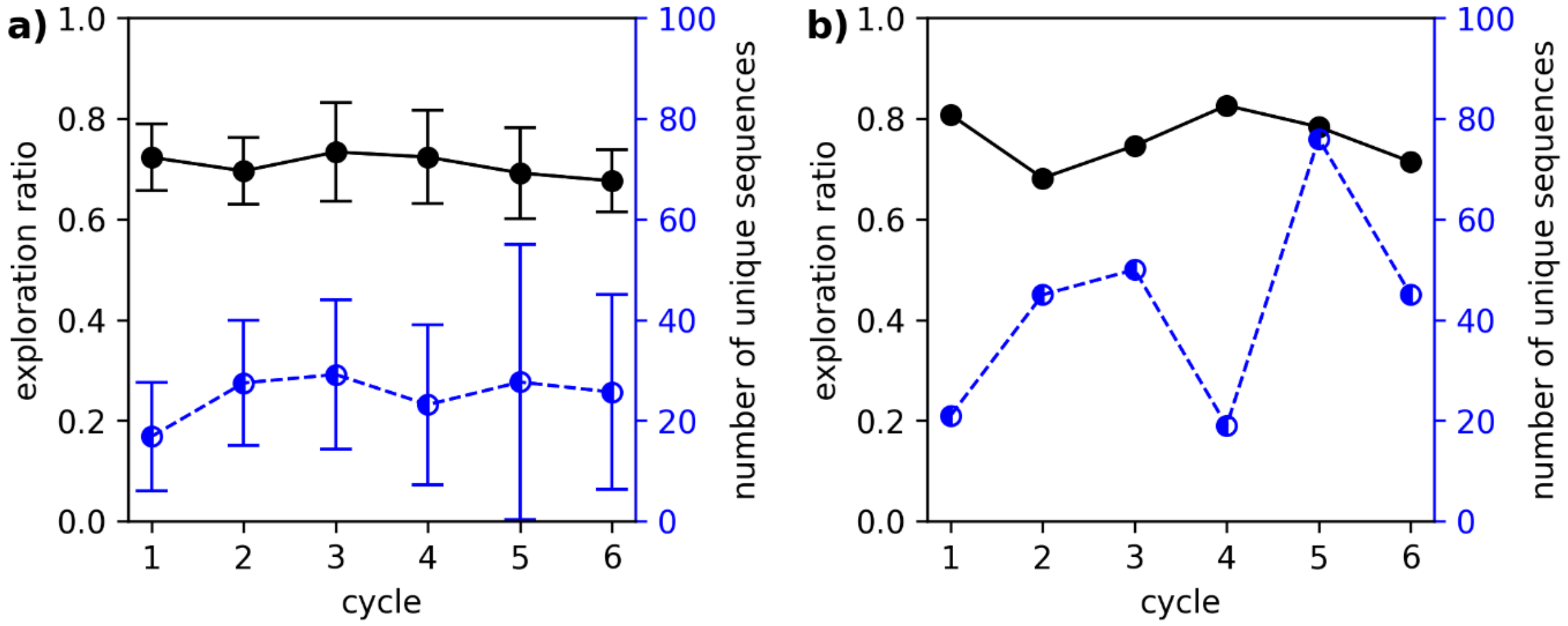

Figure S42. Number of unique peptides (blue) and exploration ratio (black) of peptide ensembles generated by agitating solid amino acid methyl esters (equimolar amounts of G, A, V, H, P) with serine proteases trypsin or chymotrypsin in ball mill. Average values for replicates a) and single trajectory b) used for bigram matrix generations (see bellow).

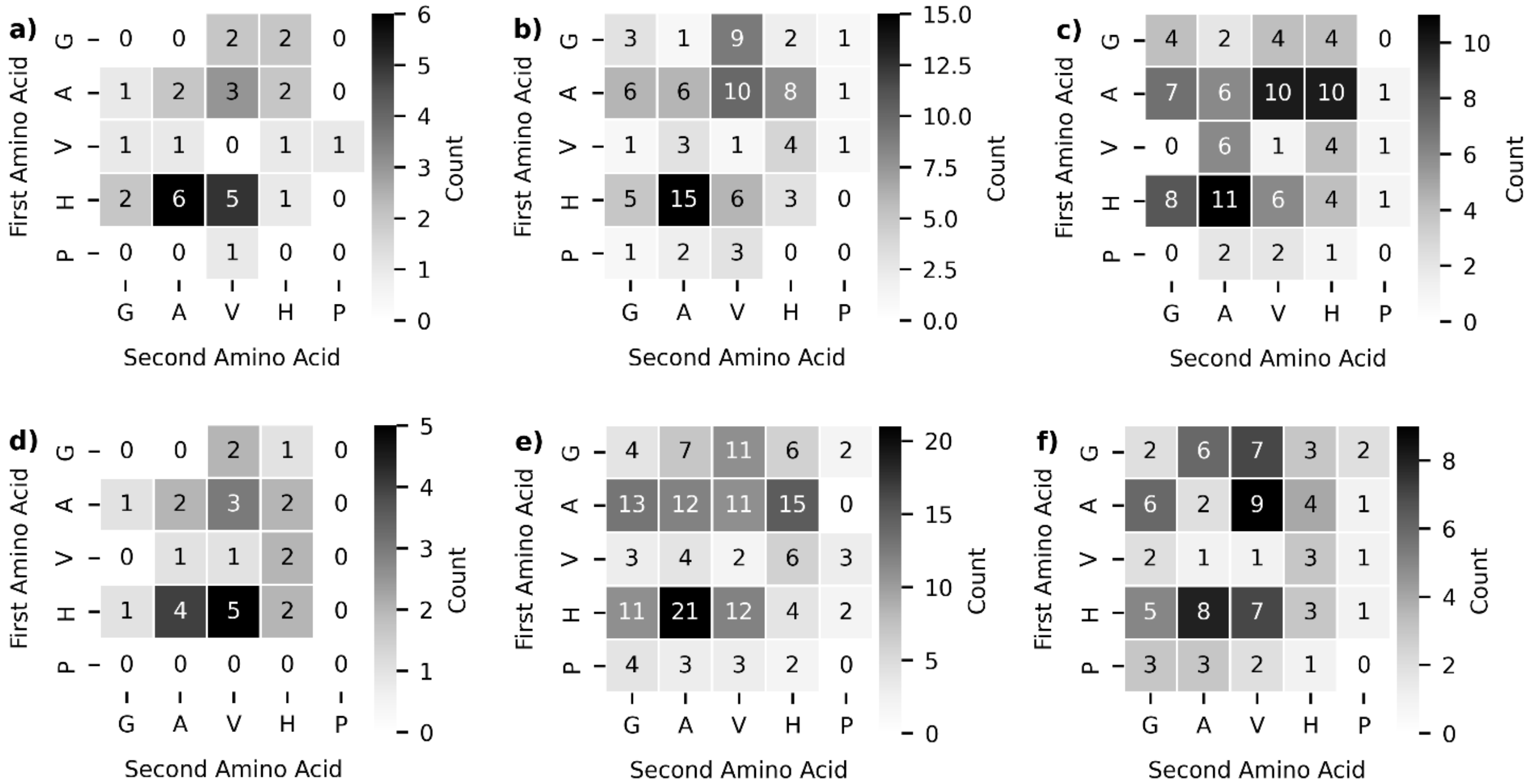

Figure S43. Bigram matrix of 2 amino acid sequence occurrence in observed peptides generated by agitating solid amino acid methyl esters (equimolar amounts of G, A, V, H, P) with serine proteases trypsin or chymotrypsin in ball mill throughout cycles from a) to h).

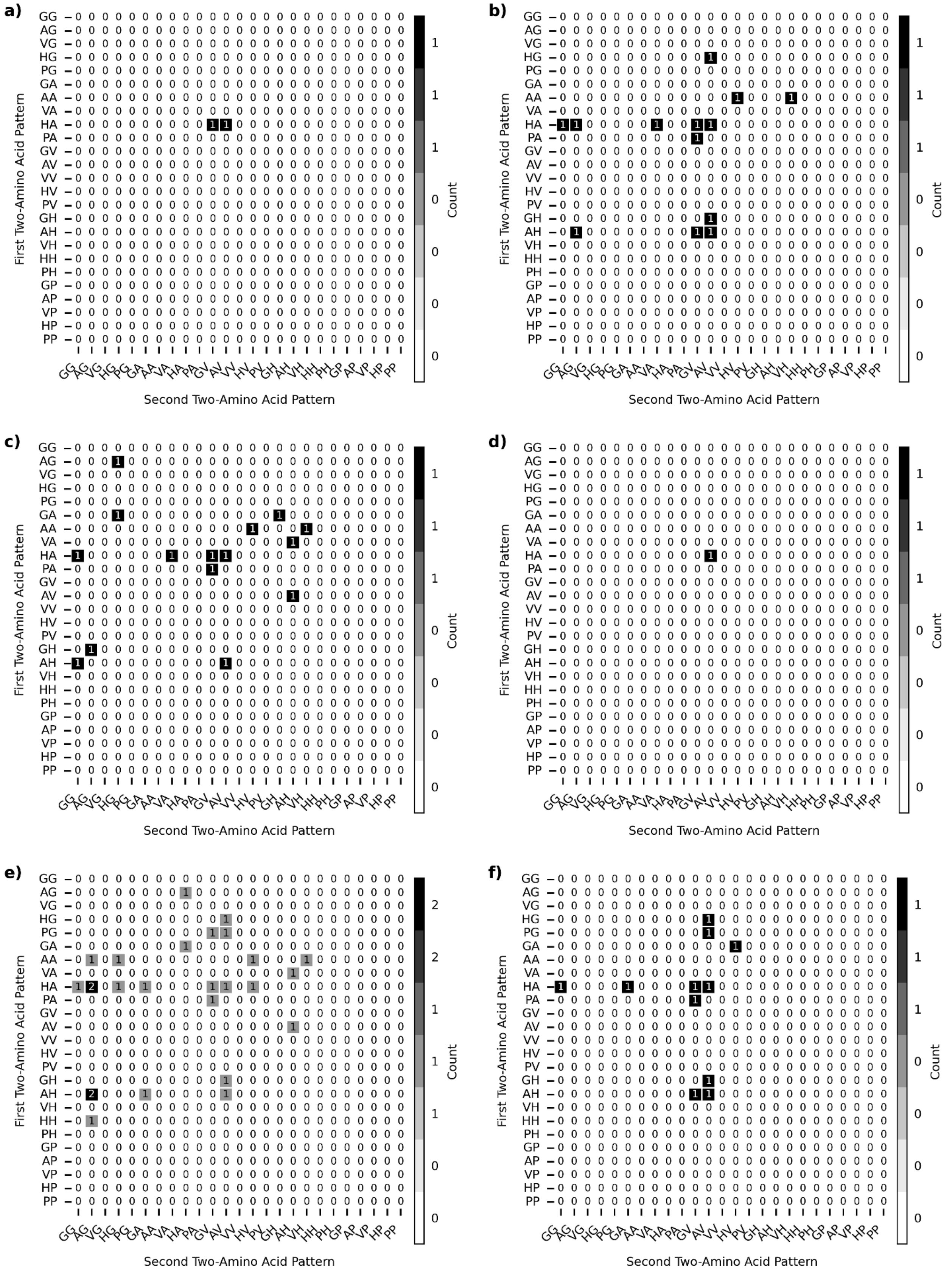

Figure S44. Bigram matrix of 4 amino acid sequence occurrence in observed peptides generated by agitating solid amino acid methyl esters (equimolar amounts of G, A, V, H, P) with serine proteases trypsin or chymotrypsin in ball mill throughout cycles from a) to h).

3.5 CDI in solution of uneven ratios

To investigate whether stoichiometric imbalances in amino acid starting materials could introduce bias into the peptide formation process and affect the exploration ratio, we systematically examined CDI-mediated coupling reactions using uneven ratios of the five amino acids (G, A, V, H, P). This experimental design was motivated by the need to understand how substrate availability might influence the apparent randomness of peptide assembly and whether exploration ratios would remain consistently high under conditions that deviate from equimolar starting compositions. Three distinct ratio combinations were selected from a comprehensive screen of 60 randomized experiments (**Table S1**): [31:17:18:33:1], [32:16:15:31:6], and [23:39:4:21:13], where the numbers correspond to the relative proportions of G, A, V, H, P respectively. These specific ratios were chosen based on their distinctive characteristics observed in the initial screening. The selection encompasses scenarios ranging from moderate imbalance to extreme depletion of specific amino acids (such as proline at 1% in the first ratio or valine at 4% in the third ratio). The experimental protocol followed the same CDI-mediated coupling procedure established for equimolar conditions, with stock solutions prepared to achieve the desired ratios and reactions conducted over multiple cycles to monitor the temporal evolution of peptide diversity and assembly characteristics. The results from these uneven ratio experiments serve as benchmark for validating that high exploration ratios observed in equimolar CDI reactions reflect genuine undirected exploration rather than artifacts of balanced substrate availability and reactivity.

3.5.1 [G, A, V, H, P] of ratio [31:17:18:33:1]

The tested ratio is of [G, A, V, H, P] was [31:17:18:33:1]. There was a clear steady increase in the number of unique objects. The exploration ratio was high, in the range of 0.8–0.9.

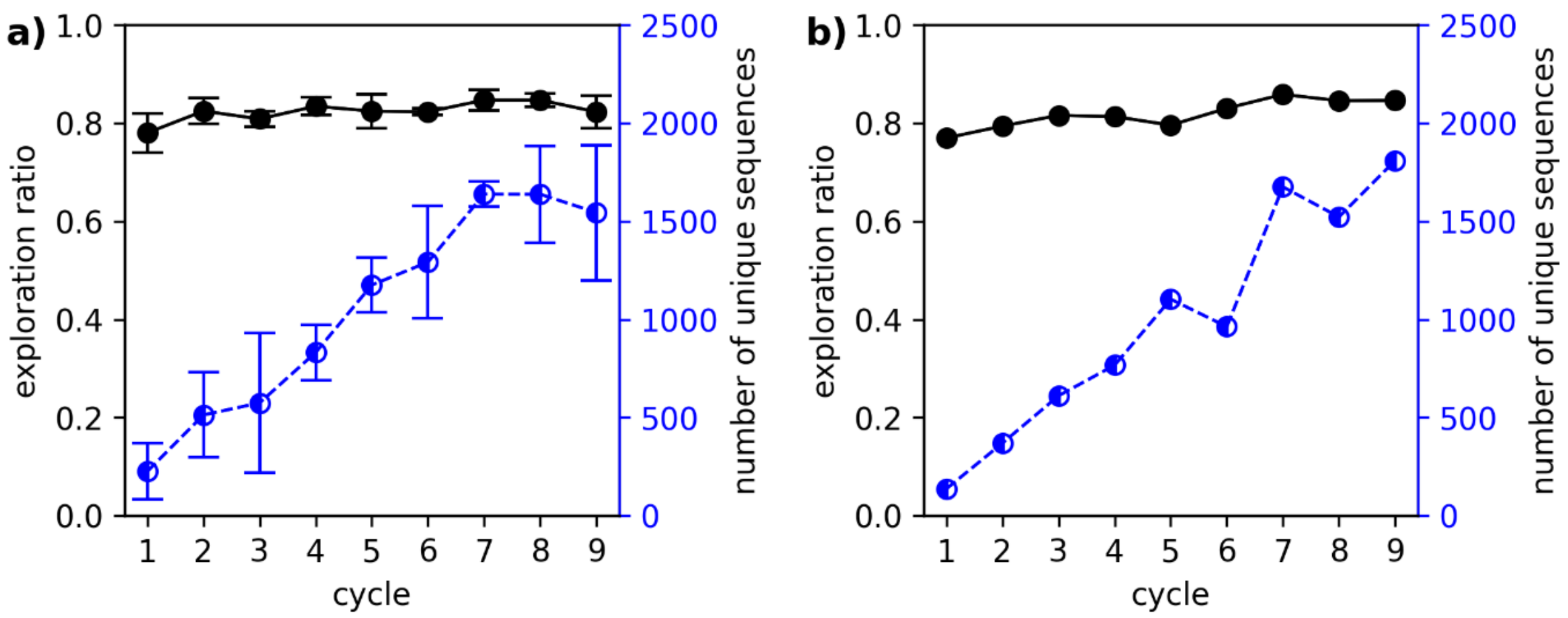

Figure S45. Number of unique peptides (blue) and exploration ratio (black) of peptide ensembles generated by addition of CDI solution to aqueous solutions of amino acids [G, A, V, H, P] with uneven ratios of [31:17:18:33:1] throughout cycles. Average values for replicates a) and single trajectory b) used for bigram matrix generations (see bellow).

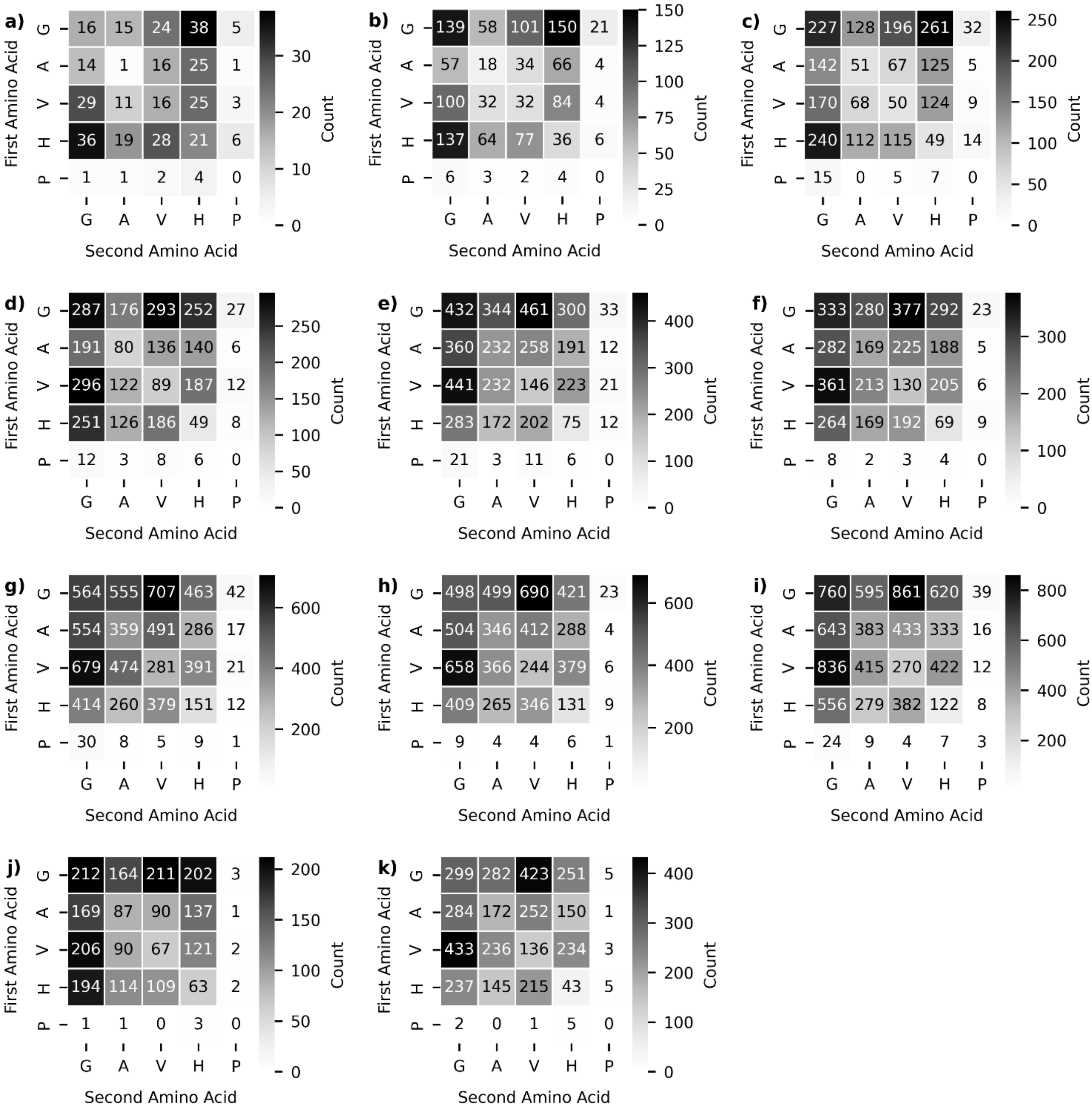

Figure S46. Bigram matrix of 2 amino acid sequence occurrence in observed peptides generated by addition of CDI solution to aqueous solutions of amino acids [G, A, V, H, P] with uneven ratios of [31:17:18:33:1] throughout cycles from a) to k).

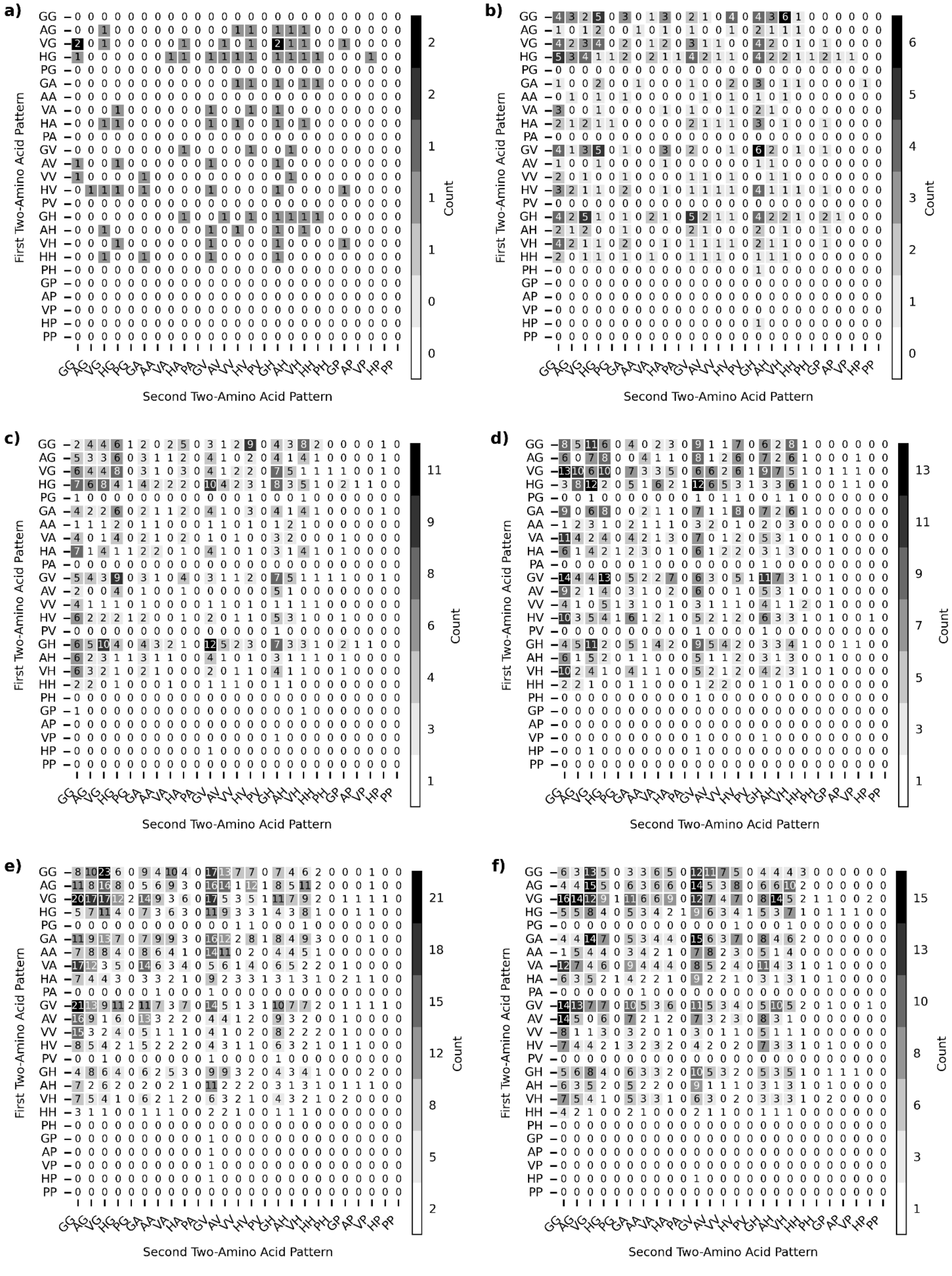
a)
b)
c)
d)
e)
f)
First Two-Amino Acid Pattern
Second Two-Amino Acid Pattern
Count
GG AG VG HG PG GA AA VA HA PA GV AV VV HV PV GH AH VH HH PH GP AP VP HP PP

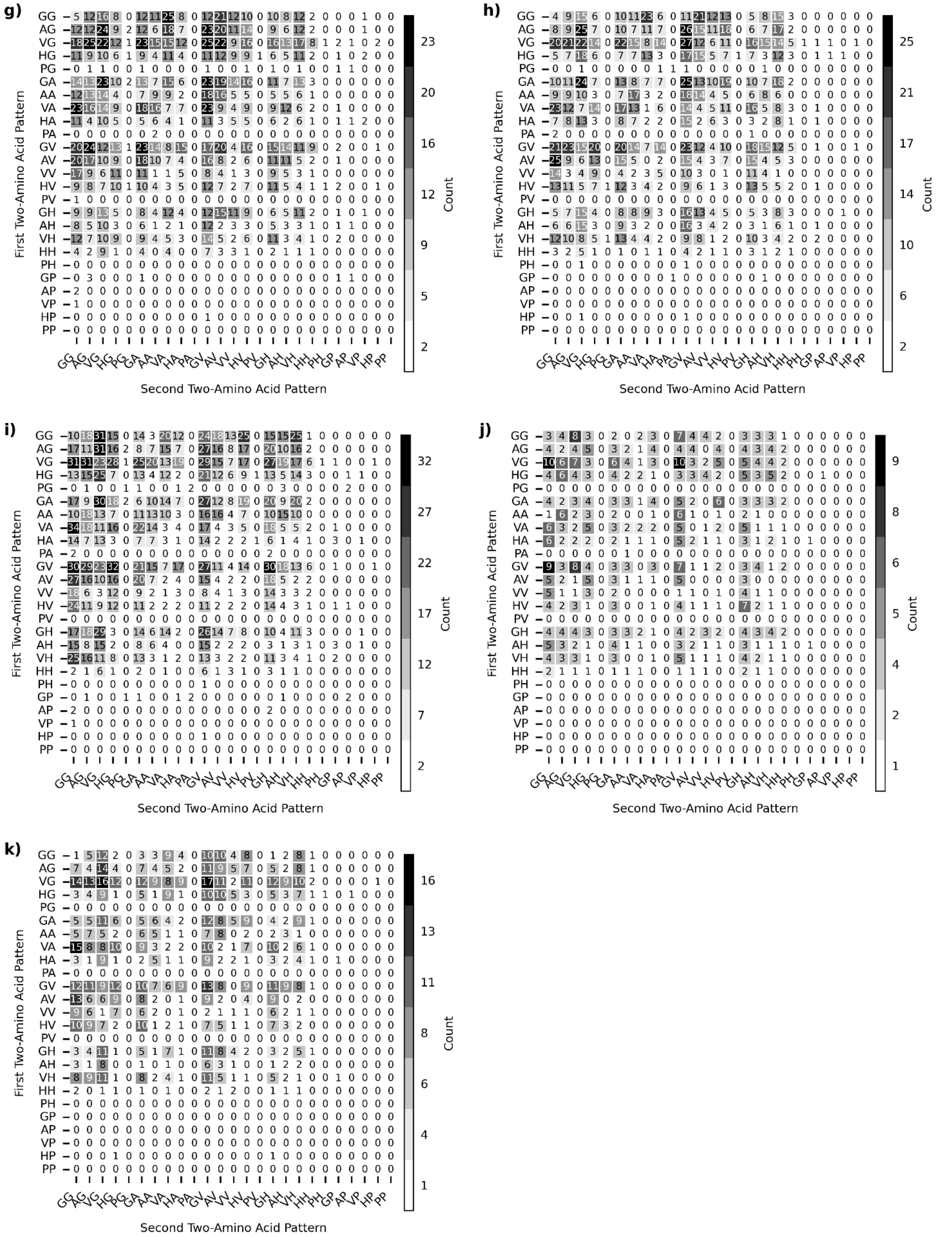

Figure S47. Bigram matrix of 4 amino acid sequence occurrence in observed peptides generated by addition of CDI solution to aqueous solutions of amino acids [G, A, V, H, P] with uneven ratios of [31:17:18:33:1] throughout cycles from a) to k).

3.5.2 [G, A, V, H, P] of ratio [32:16:15:31:6]

The tested ratio is of [G, A, V, H, P] was [32:16:15:31:6]. There was a clear steady increase in the number of unique objects. The exploration ratio was high, in the range of 0.8–0.9.

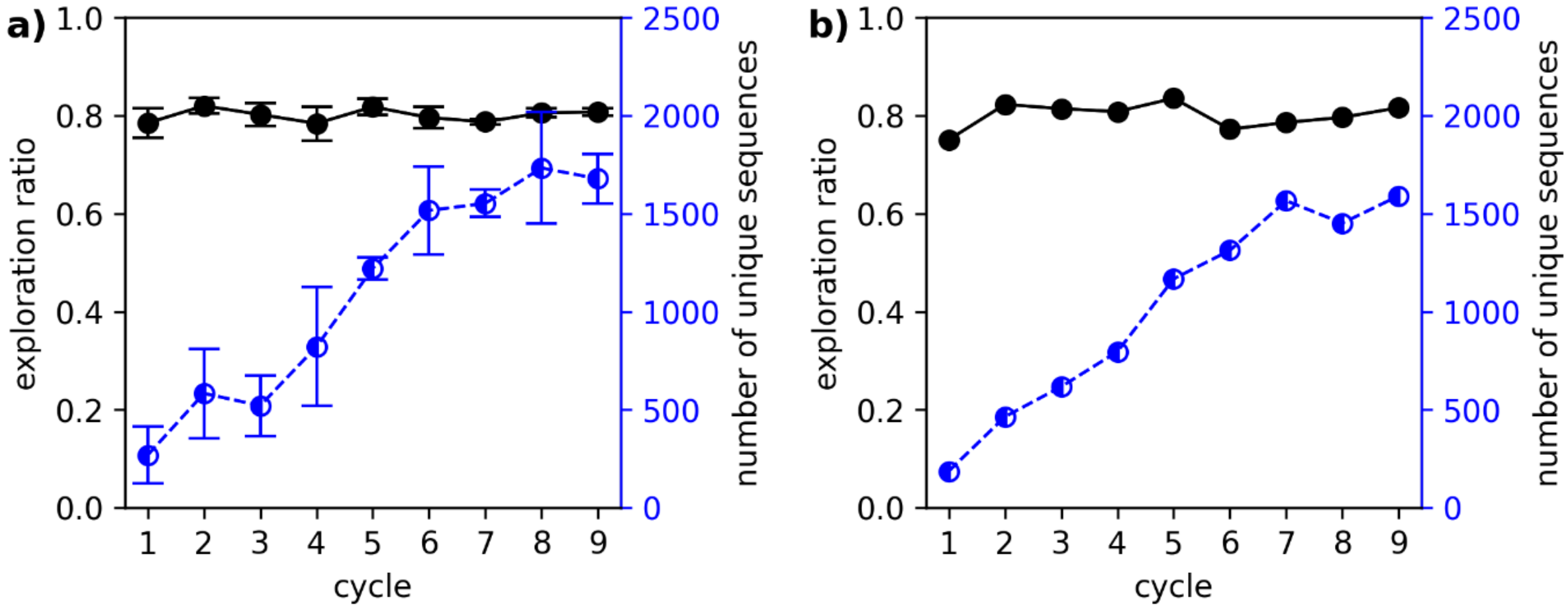

Figure S48. Number of unique peptides (blue) and exploration ratio (black) of peptide ensembles generated by addition of CDI solution to aqueous solutions of amino acids [G, A, V, H, P] with uneven ratios of [32:16:15:31:6] throughout cycles. Average values for replicates a) and single trajectory b) used for bigram matrix generations (see bellow).

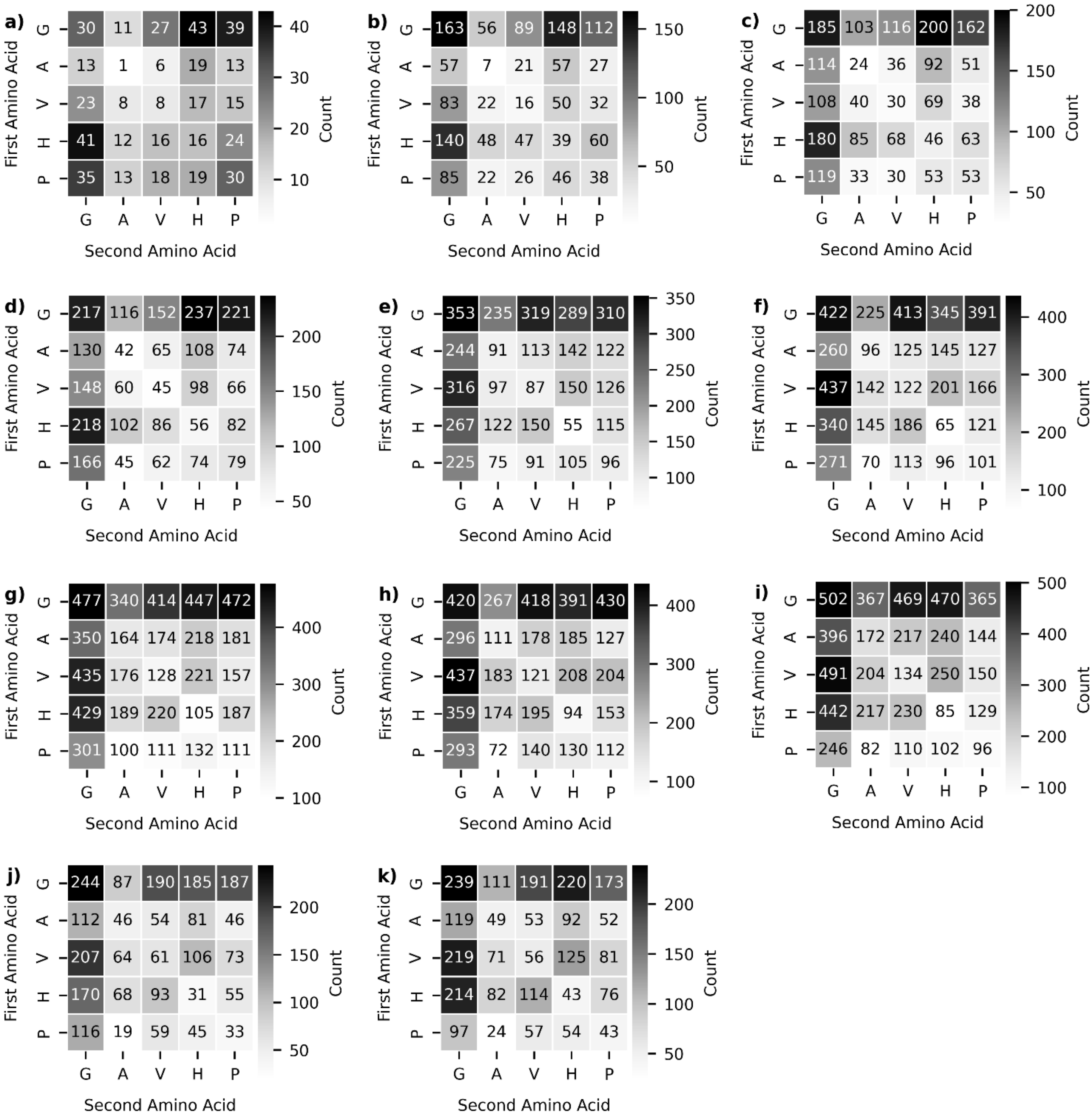

Figure S49. Bigram matrix of 2 amino acid sequence occurrence in observed peptides generated by addition of CDI solution to aqueous solutions of amino acids [G, A, V, H, P] with uneven ratios of [32:16:15:31:6] throughout cycles from a) to k).

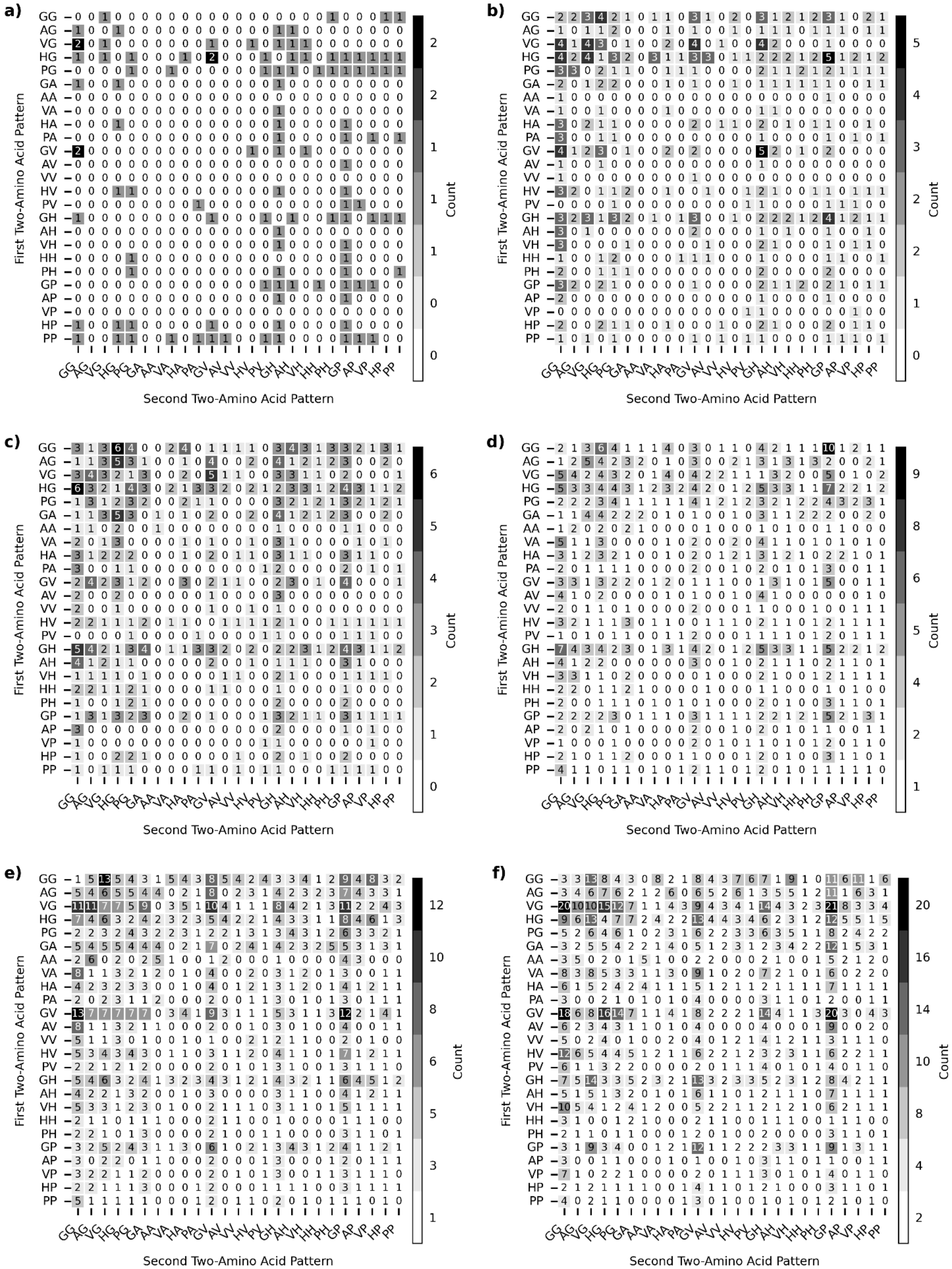
a)
b)
c)
d)
e)
f)
First Two-Amino Acid Pattern
Second Two-Amino Acid Pattern
Count
GG AG VG HG PG GA AA VA HA PA GV AV VV HV PV GH AH VH HH PH GP AP VP HP PP

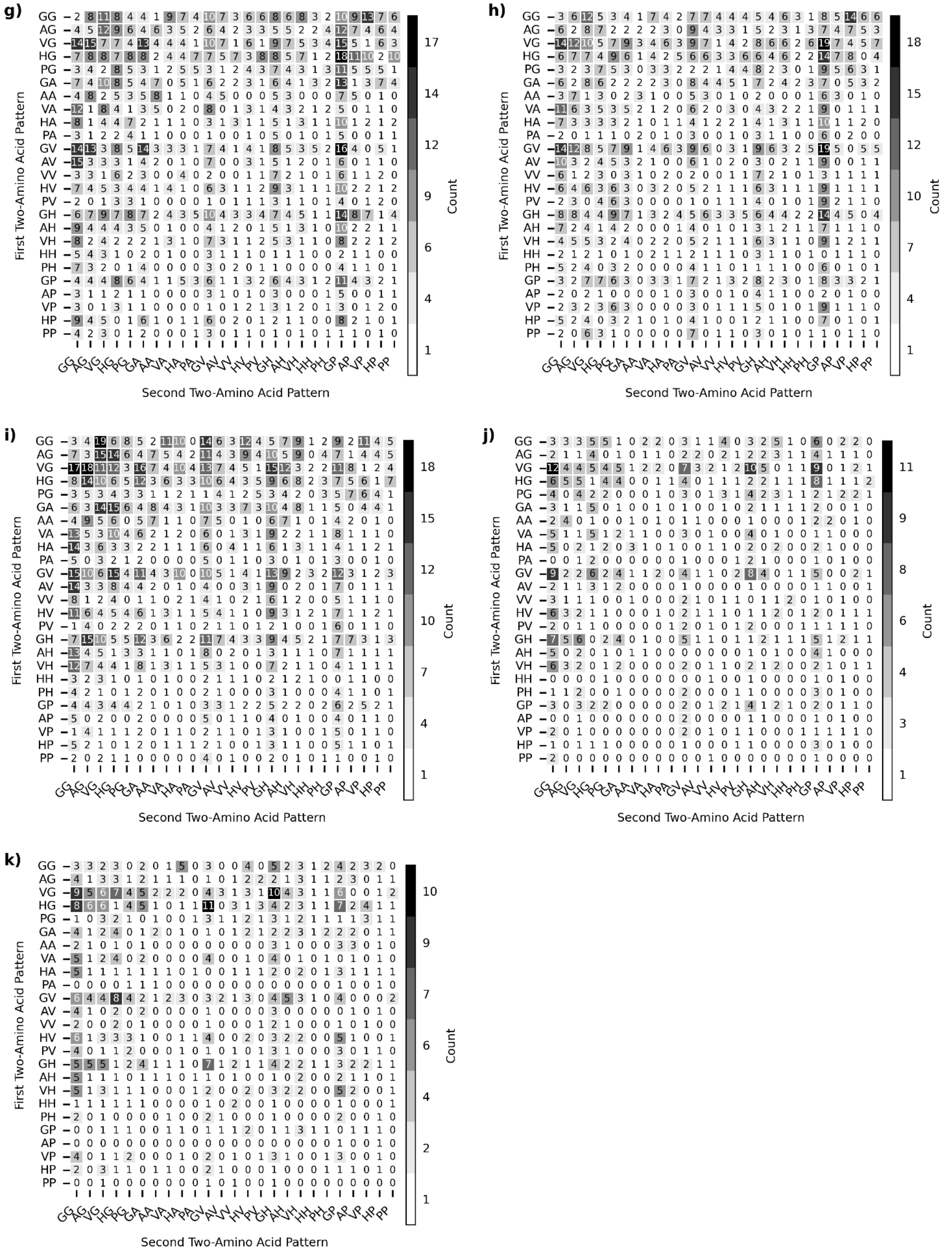

Figure S50. Bigram matrix of 4 amino acid sequence occurrence in observed peptides generated by addition of CDI solution to aqueous solutions of amino acids [G, A, V, H, P] with uneven ratios of [32:16:15:31:6] throughout cycles from a) to k).

3.5.3 [G, A, V, H, P] of ratio [23:39:4:21:13]

The tested ratio is of [G, A, V, H, P] was [23:39:4:21:13]. There was a clear steady increase in the number of unique objects. The exploration ratio was high, in the range of 0.8–0.9.

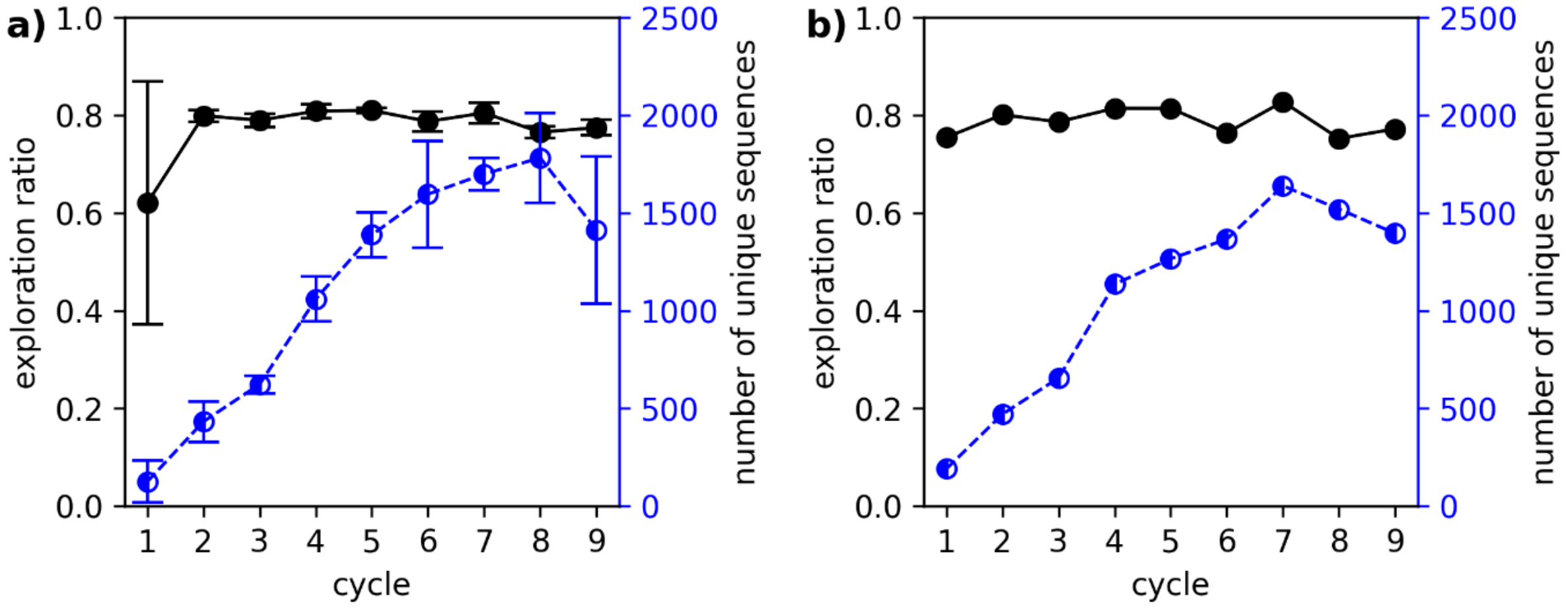

Figure S51. Number of unique peptides (blue) and exploration ratio (black) of peptide ensembles generated by addition of CDI solution to aqueous solutions of amino acids [G, A, V, H, P] with uneven ratios of [23:39:4:21:13] throughout cycles. Average values for replicates a) and single trajectory b) used for bigram matrix generations (see bellow).

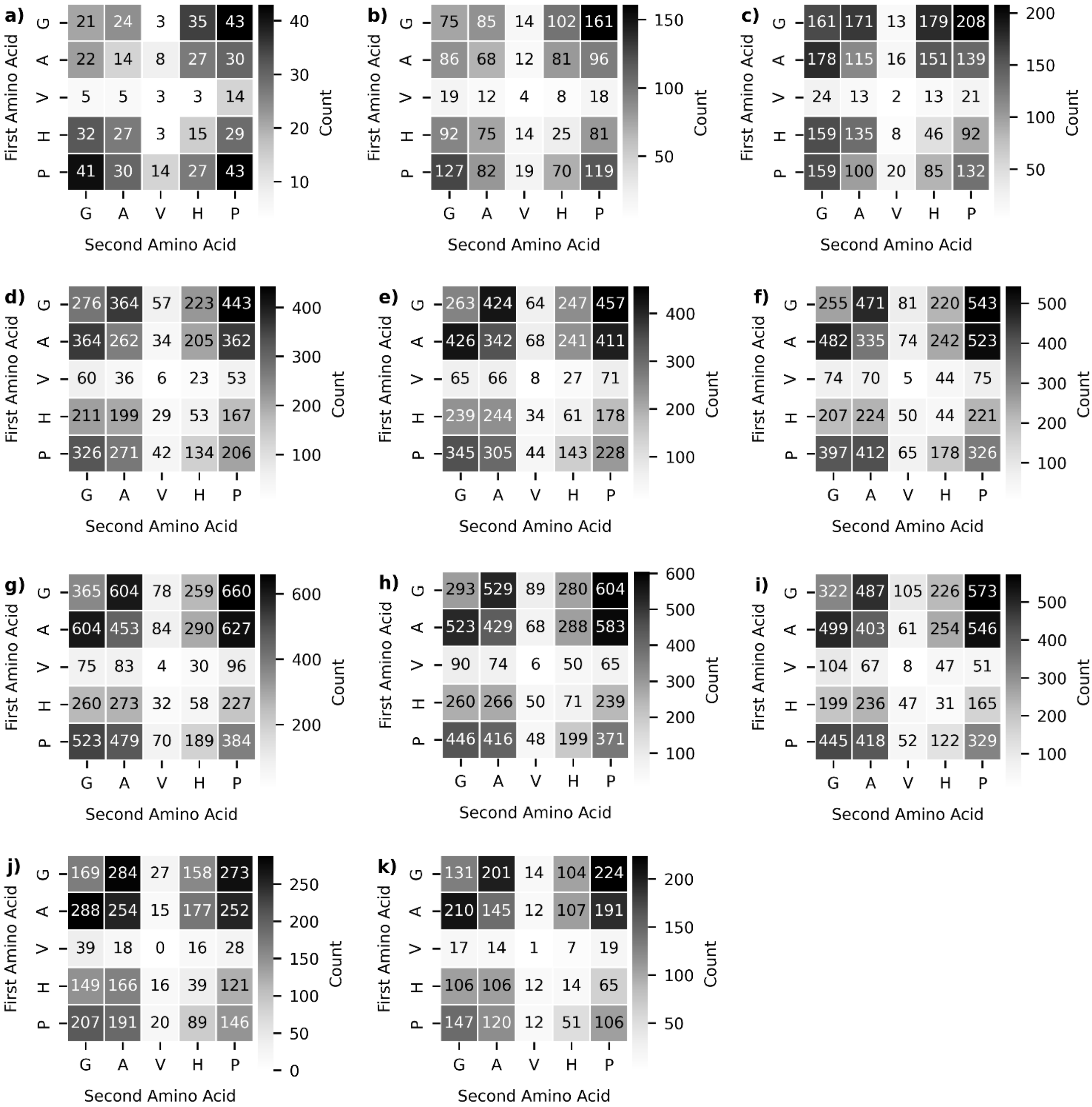

Figure S52. Bigram matrix of 2 amino acid sequence occurrence in observed peptides generated by addition of CDI solution to aqueous solutions of amino acids [G, A, V, H, P] with uneven ratios of [23:39:4:21:13] throughout cycles from a) to k).

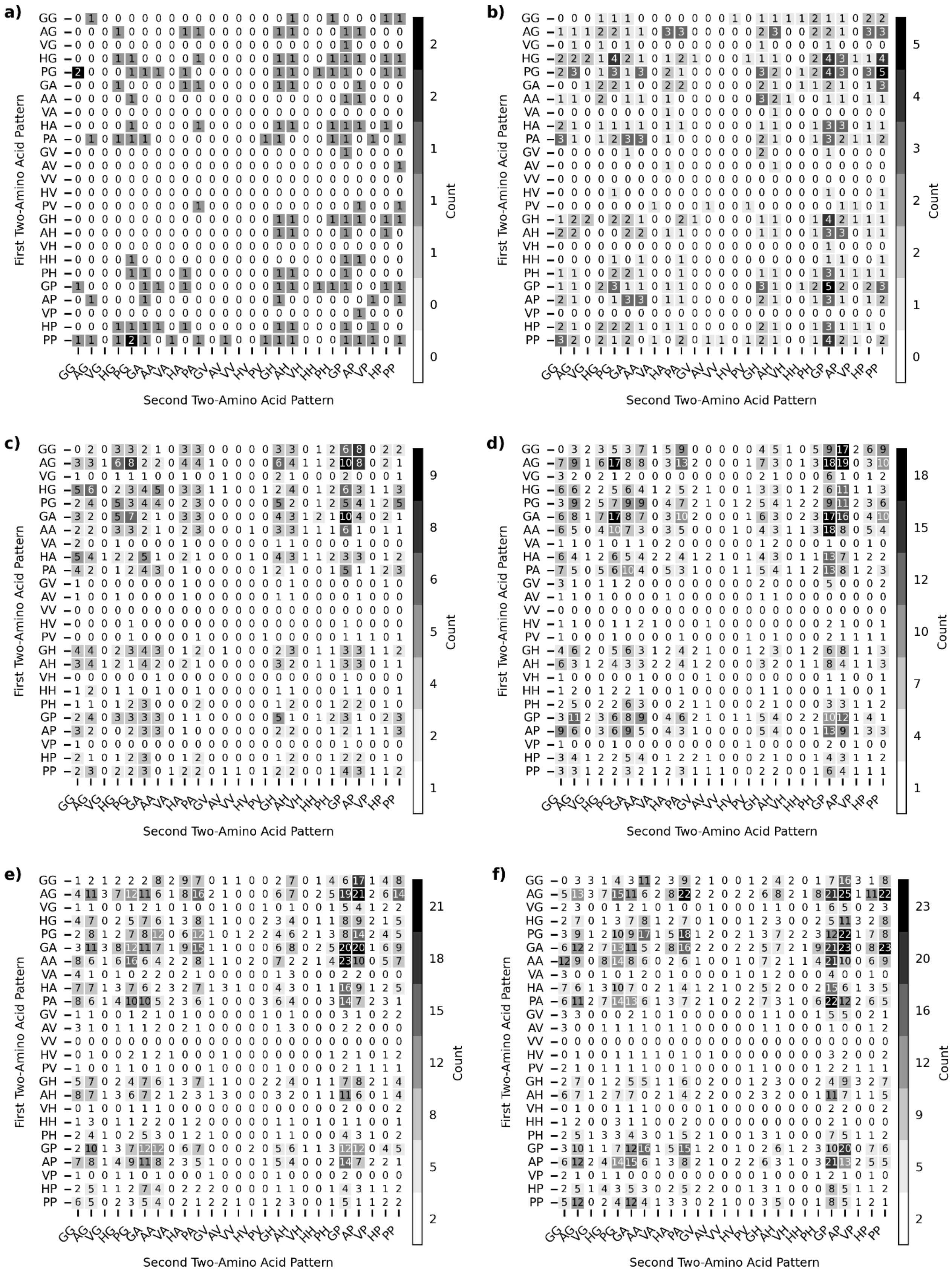
a)
b)
c)
d)
e)
f)
First Two-Amino Acid Pattern
Second Two-Amino Acid Pattern
Count

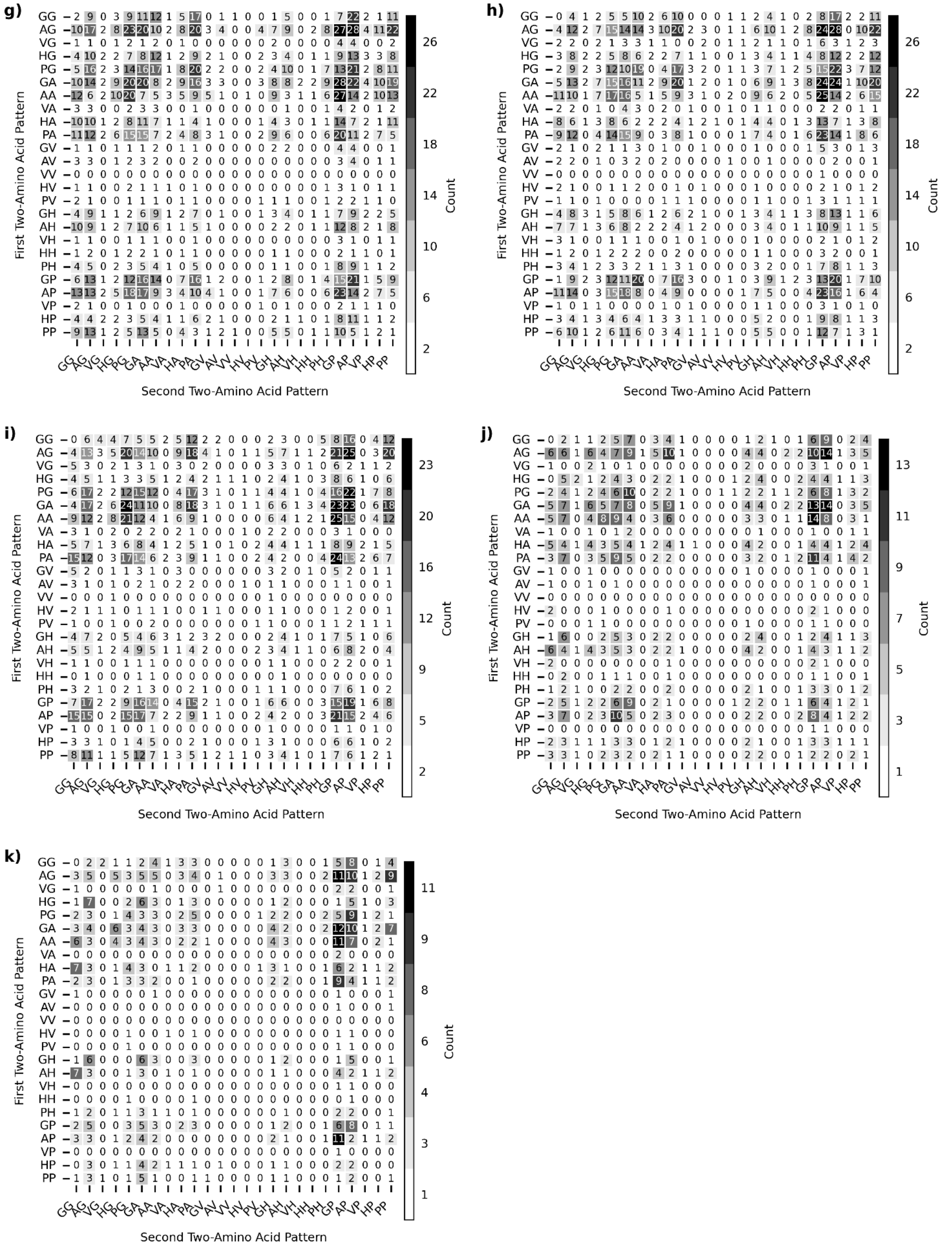

Figure S53. Bigram matrix of 4 amino acid sequence occurrence in observed peptides generated by addition of CDI solution to aqueous solutions of amino acids [G, A, V, H, P] with uneven ratios of [23:39:4:21:13] throughout cycles from a) to k).

3.5.4 Exploring various ratios of amino acids

To address the possible bias of the amino acid reactivity and natural pH buffering, we have sample 60 random ratios of G, A, V, H, P. Total 10 mmol of amino acids was added in a reactor in 1.5 mL water and 0.8 mL of 0.5 M CDI in acetonitrile (10 mmol) was added. Reaction was agitated at 40 °C for 1h and analysed on LC-MS/MS. The overall results are summarised in **Table S1**:

exp. no	G	A	V	H	P	complexity	diversity	max MA	er
1	0.346	0.372	0.369	0.057	0.156	203792	72554	9	0.79
2	0.000	0.056	0.574	0.317	0.353	19725	7749	8	0.91
3	0.195	0.397	0.020	0.295	0.393	155226	54981	9	0.80
4	0.103	0.352	0.345	0.348	0.151	96656	34797	8	0.77
5	0.092	0.319	0.349	0.378	0.162	72968	26207	8	0.77
6	0.037	0.241	0.225	0.479	0.317	61038	23584	8	0.85
7	0.465	0.298	0.254	0.146	0.137	293669	101150	10	0.77
8	0.260	0.294	0.097	0.445	0.205	251042	87779	9	0.77
9	0.529	0.202	0.159	0.063	0.347	280909	99622	10	0.82
10	0.179	0.128	0.306	0.302	0.385	110099	40488	9	0.81
11	0.299	0.411	0.193	0.304	0.093	267691	94636	9	0.78
12	0.593	0.247	0.077	0.119	0.264	297298	98748	10	0.78
13	0.603	0.077	0.320	0.155	0.146	204937	65824	10	0.73
14	0.210	0.337	0.264	0.144	0.344	190902	70724	9	0.82
15	0.171	0.098	0.493	0.013	0.524	94339	33495	9	0.83
16	0.304	0.506	0.051	0.273	0.165	346286	122109	10	0.80
17	0.061	0.326	0.264	0.342	0.307	76073	28780	8	0.83
18	0.099	0.300	0.302	0.312	0.287	90130	33257	8	0.80
19	0.145	0.302	0.376	0.121	0.356	127753	49641	8	0.86
20	0.405	0.226	0.236	0.425	0.008	503847	193601	10	0.89
21	0.041	0.297	0.392	0.041	0.529	74029	27782	9	0.88
22	0.061	0.297	0.328	0.564	0.050	50871	19000	8	0.81
23	0.355	0.327	0.189	0.286	0.144	306819	108450	9	0.78
24	0.330	0.159	0.406	0.354	0.051	140325	51519	9	0.81
25	0.403	0.010	0.207	0.250	0.430	133055	42603	10	0.75
26	0.302	0.546	0.207	0.098	0.147	262688	91377	9	0.78
27	0.437	0.151	0.165	0.125	0.422	176977	63304	9	0.82
28	0.290	0.090	0.331	0.217	0.372	138878	49962	9	0.80
29	0.271	0.077	0.097	0.399	0.456	92705	32384	9	0.79
30	0.291	0.088	0.379	0.026	0.516	133177	46193	10	0.79
31	0.109	0.380	0.400	0.132	0.279	93126	34999	8	0.82
32	0.012	0.423	0.081	0.341	0.444	45885	15813	8	0.80
33	0.025	0.052	0.419	0.438	0.365	28661	10680	8	0.85
34	0.519	0.156	0.012	0.612	0.000	20876	7017	9	0.82
35	0.136	0.396	0.275	0.077	0.415	53320	21234	7	0.88
36	0.425	0.107	0.126	0.324	0.317	5527	2195	6	0.86
37	0.200	0.270	0.323	0.445	0.062	41028	16921	7	0.88
38	0.431	0.130	0.258	0.268	0.212	21265	8219	7	0.84
39	0.419	0.202	0.194	0.402	0.082	268368	186871	10	0.79
40	0.108	0.505	0.522	0.128	0.037	42527	16021	8	0.83
41	0.410	0.329	0.174	0.257	0.129	107935	39996	8	0.82
42	0.063	0.218	0.542	0.427	0.049	16276	6594	6	0.86
43	0.127	0.289	0.381	0.117	0.385	54507	21258	7	0.85
44	0.350	0.118	0.086	0.153	0.593	13315	4715	7	0.79
45	0.183	0.055	0.364	0.344	0.354	50346	18889	8	0.82
46	0.222	0.541	0.264	0.078	0.195	159369	60537	8	0.84
47	0.346	0.552	0.030	0.014	0.358	68984	24800	9	0.84
48	0.151	0.423	0.286	0.419	0.021	8930	3699	6	0.86
49	0.352	0.141	0.374	0.302	0.130	72488	27118	8	0.82
50	0.517	0.032	0.008	0.603	0.140	21366	8158	8	0.91
51	0.224	0.295	0.297	0.185	0.299	170769	62246	8	0.80
52	0.239	0.308	0.137	0.039	0.577	140511	51651	9	0.84
53	0.166	0.360	0.351	0.140	0.283	118179	44107	8	0.82
54	0.312	0.275	0.305	0.145	0.262	137402	49332	8	0.79
55	0.288	0.439	0.040	0.163	0.371	79128	29007	8	0.83
56	0.423	0.284	0.062	0.309	0.221	41422	15893	8	0.85
57	0.395	0.258	0.077	0.228	0.341	67600	25053	8	0.83
58	0.255	0.115	0.316	0.383	0.230	42907	16663	7	0.83
59	0.203	0.202	0.436	0.264	0.196	53368	20367	7	0.82
60	0.189	0.000	0.435	0.452	0.224	45757	16894	8	0.81

Table S1. Analysis of 60 randomized experiments using varying ratios of amino acids (G, A, V, H, P) with CDI coupling agent. While diversity (unique products) showed variation between samples, the exploration ratio consistently remained between 0.8-0.9.

4 Experimental Details – Analytical Techniques (HPLC-MS/MS)

4.1 Chromatography

The samples were suspended in water and filtered through a 0.45 μm nylon syringe filter prior to MS analysis. The mobile phase was composed of water (A) and acetonitrile (B) with 0.1 % of formic acids (in both solvents). The eluent flow was set to 0.6 mL/min and gradient of solvent set to starting with 2% of B for 3 minutes and gradually increasing to 5% over 10 minutes and further to 98% over 10 minutes. The samples were run through an UltiMate 3000 Thermo UHPLC system. The column used to analyse the samples was an Agilent poroshell C18 2.7 μm, 4.6 x 150 mm column with a flow rate of 0.6 mL/min. The chromatographic separation was then infused into the Thermo Orbitrap Fusion Lumos Tribrid MS. The acquisition was run in positive mode.

4.2 MS/MS method

Instrumentation and Analytical Setup: High-resolution mass spectrometric analysis was performed using an Orbitrap-based mass spectrometer equipped with heated electrospray ionization (H-ESI) source. The instrument was operated in positive ionization mode with an ion transfer tube temperature maintained at 300 °C and vaporizer temperature of 125 °C. Internal mass calibration was performed using EASY-IC technology to ensure mass accuracy throughout the analysis. **Chromatographic Conditions:** Liquid chromatography separation was employed with an expected chromatographic peak width of 6 seconds. The total method duration was 24 minutes, allowing sufficient separation of the analytes prior to mass spectrometric detection. **Ion Source Parameters:** The H-ESI source was operated with static spray voltage settings, with positive ion voltage set at 3750 V. Gas parameters were maintained at static mode with sheath gas, auxiliary gas, and sweep gas flow rates of 5, 4.3, and 2 arbitrary units, respectively. These parameters were optimized to ensure efficient ionization. **MS Acquisition Parameters:** MS data were acquired using the Orbitrap detector at a resolution of 120,000. The scan range was set to m/z 100-1500 to capture a wide range of small molecules. Quadrupole isolation was enabled with an RF lens setting of 55%. AGC target was set to "Standard" with automatic maximum injection time to optimize ion population in the Orbitrap. **Data-Dependent Acquisition Strategy:** A sophisticated data-dependent acquisition strategy was implemented for comprehensive structural characterization. Following each MS1 scan, up to 20 data-dependent MS^2 scans were triggered for the most abundant precursor ions using intensity threshold-based selection (2.0×10^4). Precursor ions were isolated using a quadrupole with a narrow isolation window of 0.4 m/z to ensure selectivity. **Fragmentation Parameters:** Fragment ions were generated using higher-energy collisional dissociation (HCD) with stepped collision energies of 25%, 35%, 45%, 55%, and 65% to maximize structural information from a single acquisition. MS^2 spectra were recorded with the Orbitrap at a resolution of 60,000 in centroid mode with a custom maximum injection time of 118 ms. **Dynamic**

Exclusion Settings: To maximize coverage of lower abundance species, dynamic exclusion was enabled with an exclusion duration of 25 seconds after 2 observations within a 15-second window. A narrow mass tolerance of ±2 ppm was applied for exclusion criteria, and isotopes of selected precursors were automatically excluded to avoid redundant MS^2 acquisitions.

4.3 MS/MS Processing

Raw MS/MS data were converted into a mzML format using MSConvert software.[6] This was further parsed into prefiltered JSON format containing structured information about parent peeks, and the MS2, with the associated intensities using using software MzML Ripper.[7] This data structure was processed using OLIGOSS, with modified library of oligomers to include methyl esters on C terminus in the case of polymerisation of amino acid methyl esters. Such implementation had an extra criterion for the case of methyl esters, an extra building block of CH_3 unit was included. Hits other that presence of one methyl group at the C terminus were removed as part of post-processing. Ratio of identified fragments with possible fragments for the sequence, assuming b/y fragmentation, was used as a confidence of the sequence assignment. Threshold for assigning the presence of the peptide sequence was chosen as 70%.

4.4 Copy number sensitivity analysis

In the calculation of ensemble assembly A from experimental data, we operate with simple approximation that all observed objects are of same copy number. This is based on known feature of mass spectrometry that intensity of observed species is highly dependent on ESI ionisation efficiency. Raw MS1 intensities can differ by orders of magnitude between peptides of different lengths and compositions, making them unreliable as relative abundance proxies. We therefore assign unit copy number to every sequence detected above the confidence threshold (>=70% fragment coverage), equivalent to a presence/absence measurement. This removes a systematic instrument-derived bias towards easily-ionisable (typically short) sequences.

To assess whether this choice qualitatively affects the conclusions, we performed a bounded sensitivity analysis on all experiments. We consider two extreme copy-number scenarios that together bracket the maximum directional shift under any monotonic, bounded copy-number distribution. In Scenario A (simple-biased), species with lower assembly index are assigned higher relative copy numbers, following a gradient of 0.5x to 5x across molecular assembly (MA) bins (so the least complex sequences carry 10-fold more copies than the most complex). In Scenario B (complex-biased), the gradient is reversed (5x to 0.5x). The figures below show first the copy-number distributions and their effect on ensemble assembly A for two representative experiments, and then the effect on the A vs. diversity phase-space plot across all experiments.

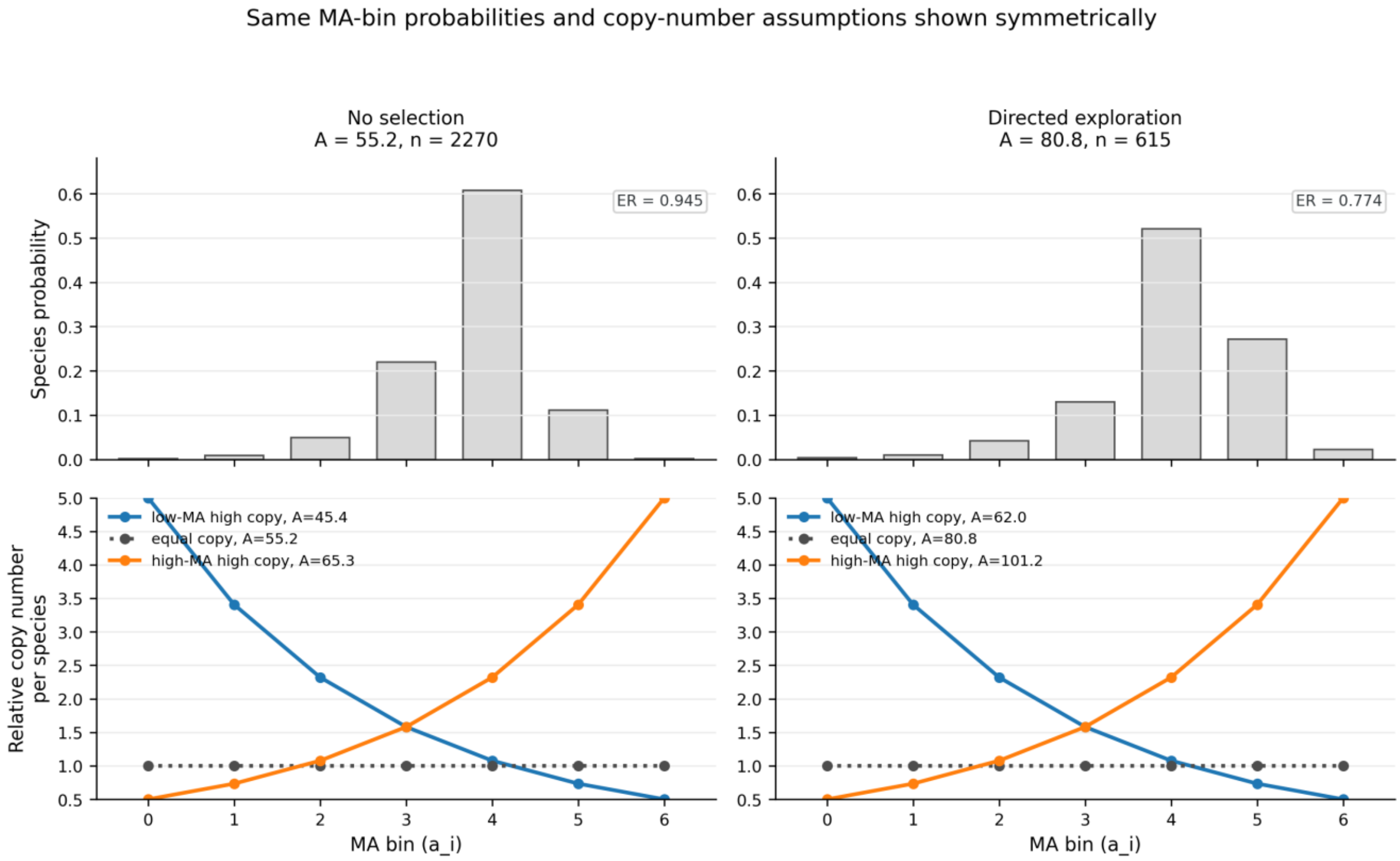

Figure S54. Copy-number scenarios and their effect on ensemble assembly A. Three copy-number assumptions were applied to two representative experiments (undirected: ER = 0.945, n = 2270; directed: ER = 0.774, n = 615). Blue: low-MA species are more abundant (0.5× to 5× gradient across MA bins). Grey: equal copy numbers, the assumption used by default in the manuscript. Orange: high-MA species are more abundant (5× to 0.5× gradient). Top panels show species probability distributions across MA bins; bottom panels show the resulting relative copy numbers per species, with ensemble assembly A for each case. The directed/undirected classification is preserved under all three assumptions.

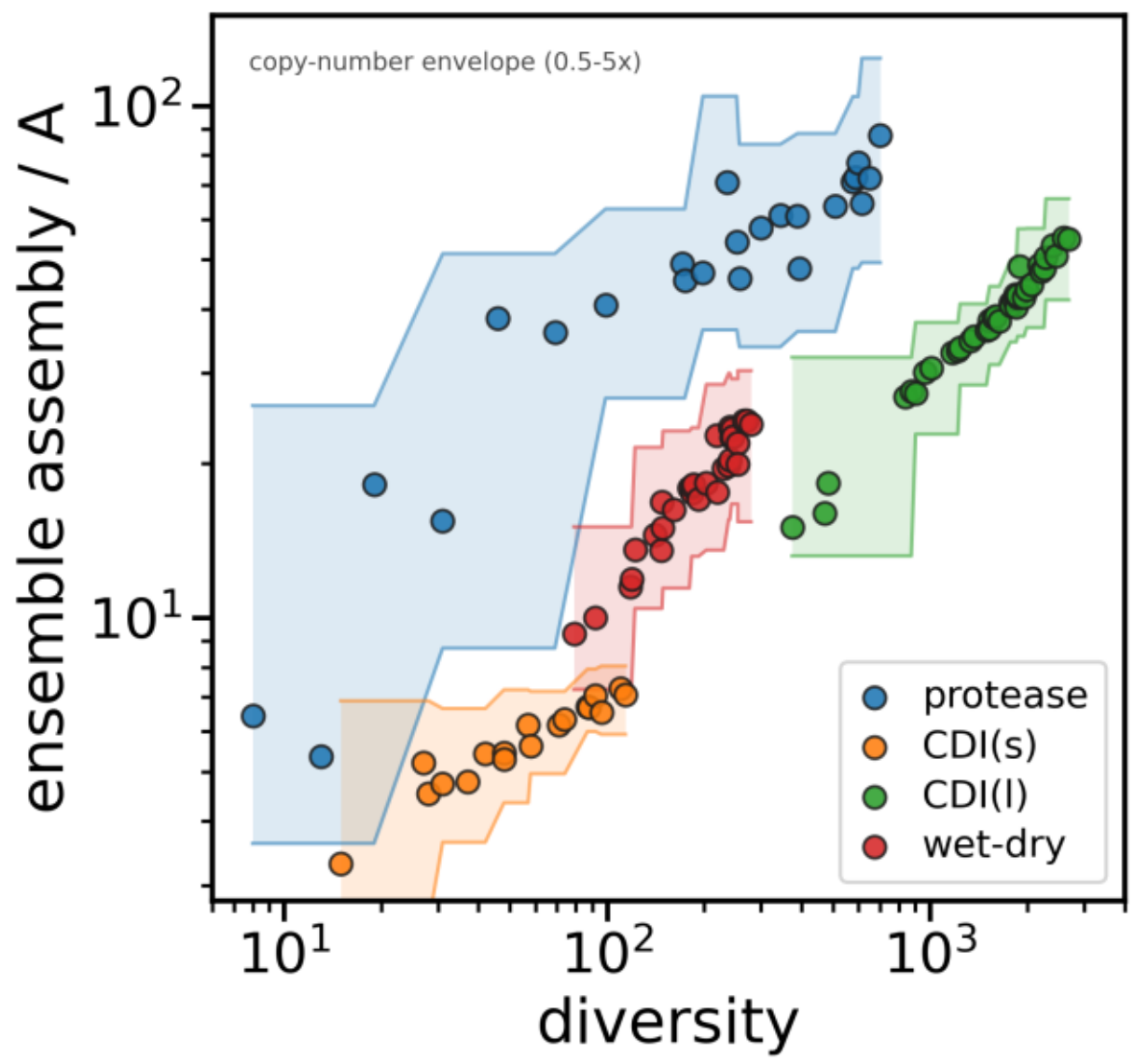

Figure S55. Copy-number sensitivity envelope for the manuscripts Figure 5h phase-space plot. Points show ensemble assembly A vs. diversity using equal copy numbers. Shaded regions span the A values under the two extreme copy-number scenarios: when low assembly-index species more abundant (0.5× to 5× gradient) and when high assembly-index species are more abundant (5× to 0.5× gradient). All four reaction families (protease, CDI(s), CDI(l), wet-dry) remain clearly separated under both assumptions.

5 References

(1) Sharma, A.; Czégel, D.; Lachmann, M.; Kempes, C. P.; Walker, S. I.; Cronin, L. Assembly Theory Explains and Quantifies Selection and Evolution. *Nature* **2023**, *622*, 321–328. https://doi.org/10.1038/s41586-023-06600-9.

(2) Jirasek, M.; Sharma, A.; Bame, J. R.; Mehr, S. H. M.; Bell, N.; Marshall, S. M.; Mathis, C.; MacLeod, A.; Cooper, G. J. T.; Swart, M.; Mollfulleda, R.; Cronin, L. Investigating and Quantifying Molecular Complexity Using Assembly Theory and Spectroscopy. *ACS Cent. Sci.* **2024**, *10* (5), 1054–1064. https://doi.org/10.1021/acscentsci.4c00120.

(3) Ardila-Fierro, K. J.; Crawford, D. E.; Körner, A.; James, S. L.; Bolm, C.; Hernández, J. G. Papain-Catalysed Mechanochemical Synthesis of Oligopeptides by Milling and Twin-Screw Extrusion: Application in the Juliá–Colonna Enantioselective Epoxidation. *Green Chem.* **2018**, *20* (6), 1262–1269. https://doi.org/10.1039/C7GC03205F.

(4) Terada, K.; Kurita, T.; Gimenez-Dejoz, J.; Masunaga, H.; Tsuchiya, K.; Numata, K. Papain-Catalyzed, Sequence-Dependent Polymerization Yields Polypeptides Containing Periodic Histidine Residues. *Macromolecules* **2022**, *55* (16), 6992–7002. https://doi.org/10.1021/acs.macromol.2c01036.

(5) Tsuchiya, K.; Numata, K. Chemoenzymatic Synthesis of Polypeptides for Use as Functional and Structural Materials. *Macromolecular Bioscience* **2017**, *17* (11), 1700177. https://doi.org/10.1002/mabi.201700177.

(6) Chambers, M. C.; Maclean, B.; Burke, R.; Amodei, D.; Ruderman, D. L.; Neumann, S.; Gatto, L.; Fischer, B.; Pratt, B.; Egertson, J.; Hoff, K.; Kessner, D.; Tasman, N.; Shulman, N.; Frewen, B.; Baker, T. A.; Brusniak, M.-Y.; Paulse, C.; Creasy, D.; Flashner, L.; Kani, K.; Moulding, C.; Seymour, S. L.; Nuwaysir, L. M.; Lefebvre, B.; Kuhlmann, F.; Roark, J.; Rainer, P.; Detlev, S.; Hemenway, T.; Huhmer, A.; Langridge, J.; Connolly, B.; Chadick, T.; Holly, K.; Eckels, J.; Deutsch, E. W.; Moritz, R. L.; Katz, J. E.; Agus, D. B.; MacCoss, M.; Tabb, D. L.; Mallick, P. A Cross-Platform Toolkit for Mass Spectrometry and Proteomics. *Nat Biotechnol* **2012**, *30* (10), 918–920. https://doi.org/10.1038/nbt.2377.

(7). https://github.com/croningp/mzmlripper.
